# Community and stakeholder engagement in national priority setting and participatory research for HIV, Tuberculosis, and Malaria programs in Nepal

**DOI:** 10.1186/s40900-026-00907-3

**Published:** 2026-05-22

**Authors:** Sandesh Neupane, Rupa Shiwakoti, Laxman Aryal, Masauso Nzima, Ivana Lohar, Achut Sitaula, Ujjwal Karmacharya, Krishna Paudel, Anuj Bhattachan, Sudha Devkota, Rajan Kumar Bhattarai, Bipin Adhikari

**Affiliations:** 1 Country Coordinating Mechanism (CCM) Nepal, Ministry of Health and Population, Kathmandu, Nepal; 2 Ministry of Health and Population, Government of Nepal, Kathmandu, Nepal; 3 Joint United Nations Programme on HIV/AIDS (UNAIDS), Kathmandu, Nepal; 4 United States Agency for International Development (USAID), Kathmandu, Nepal; 5 Civil Society Representative to the Country Coordinating Mechanism (CCM), Kathmandu, Nepal; 6 Epidemiology and Disease Control Division, Ministry of Health and Population, Government of Nepal, Kathmandu, Nepal; 7 National Tuberculosis Control Centre, Ministry of Health and Population, Government of Nepal, Kathmandu, Nepal; 8 National Centre for AIDS and STD Control, Ministry of Health and Population, Government of Nepal, Kathmandu, Nepal; 9 Save the Children International (Global Fund), Kathmandu, Nepal; 10 Mahidol-Oxford Tropical Medicine Research Unit, Faculty of Tropical Medicine, Mahidol University, Bangkok, Thailand; 11 Centre for Tropical Medicine and Global Health, Nuffield Department of Medicine, University of Oxford, Oxford, UK

**Keywords:** Community and stakeholder engagement approach to develop priorities for national HIV, TB and malaria programs in Nepal

## Abstract

**Background:**

Following Nepal’s transition to a federal governance system under the 2015 Constitution and the COVID-19 pandemic, the Country Coordinating Mechanism (CCM) led a participatory process to identify national priorities for HIV, Tuberculosis (TB), and Malaria programs. The primary objective of this study was to analyze, in collaboration with community partners, the community and stakeholder engagement processes involved in priority setting.

**Methods:**

Between January and June 2020, a qualitative study was conducted, including a series of community group discussions across all seven provinces and 72 municipalities. This qualitative documentation process involved over 2,000 participants across 700 sessions across all three tiers of governance. These sessions included key and vulnerable people affected by TB and HIV, as well as at-risk populations for malaria. In addition, 100 key informant interviews were held with government officials, technical experts, and civil society leaders. The analysis used both deductive and inductive methods, with involvement and input from the community stakeholders.

**Results:**

The engagement process offered a space for broad and inclusive participation, with most stakeholders involved in priority setting representing key or vulnerable populations. Participants reported that recommendations, such as establishing HIV testing points for migrants at border areas, were later included in national strategic documents. Many of the system gaps identified during multi-stakeholder discussions, including drug stock outs, stigma in care, and human rights and gender related barriers were reflected in national planning processes, indicating perceived influence of the engagement process. Participants also described practical recommendations, including integration of multi-disease diagnostic initiatives (for Tuberculosis and HIV for instance, using single platforms such as GeneXpert) and strengthening community-level surveillance systems, which were considered during strategic discussions. These priorities were reflected in strategic plans and referenced by the partners (e.g. Global Fund, USAID/PEPFAR). The government further pledged 20% domestic co-financing for the programs, contributing to a sense of ownership within Nepal’s federal system. Participants further reported that the process supported transparency, trust and a sense of ownership.

**Conclusions:**

CSE helped ensure that national health priorities were grounded in community realities, broadly inclusive and strategically aligned with institutional frameworks. Engaging community members as active partners in data collection, interpretation, and validation reflected the participatory nature of the entire research process. This approach may offer insights for other low- and middle-income countries.

**Supplementary Information:**

The online version contains supplementary material available at 10.1186/s40900-026-00907-3.

## Plain language summary

Nepal’s recent shift to a new federal system and the challenges of the COVID-19 pandemic created an urgent need for more inclusive decision-making in health. The Country Coordinating Mechanism (CCM), which oversees Global Fund-supported health programs, led a participatory process to set national priorities for HIV, tuberculosis (TB), and malaria programs for 2021–2024. Community and stakeholder involvement was a key aspect of the process, including participation in data analysis, interpretation, and the preparation of this report. Between January and June 2020, more than 2,000 people took part in over 700 local consultation meetings across all seven provinces. These meetings included people living with HIV, TB survivors, sex workers, transgender people, people who inject drugs, migrants, prisoners, and female community health volunteers. Around 100 interviews were also held with government officials, experts, and civil society leaders to capture diverse viewpoints. This engagement process provided an opportunity for communities to contribute directly to national health planning. About 85% of participants were from key or vulnerable populations. Many of their ideas were later adopted in national strategies, such as establishing HIV testing sites for migrants at border areas, addressing stigma in TB care, and improving drug supply systems. Communities also recommended integrating TB and HIV testing using shared diagnostic machines like GeneXpert. Even during the pandemic, virtual consultations increased participation by about 40%, and the government committed 20% domestic co-financing for these programs, indicating increased ownership. This experience suggests that inclusive community and stakeholder engagement can support the development of more practical, equitable, and locally grounded health policies, offering valuable lessons for other countries with limited resources or transitioning governance systems.

## Background

Community and stakeholder engagement (CSE) is the collaborative process of working with groups of people to address issues that affect them and to achieve mutual outcomes [1–5]. CSE across the policy cycle, from problem identification to evaluation, can play an important role in understanding the perspectives of government, community networks, civil society organizations (CSOs), and development partners [6–8]. It can also help promote equity in policymaking when there are competing priorities [9–11]. CSE has been described as a valuable element in the design and implementation of research and public health interventions [1, 3, 4, 12]. Depending on the context, purpose, resources, and distribution of decision-making power, engagement may take different forms, from informing and consulting to involving, collaborating, and empowering stakeholders [13]. In recent years, increasing attention has been given to research priority setting that aligns with communities’ identified health concerns and research needs, notwithstanding the constraints that may limit their effective participation in the process [14–18]. This spectrum from consultation to collaboration, co-production and community-led research provides a useful lens to situate the approach used in this study.

In global health discussions, CSE is often viewed as contributing to efforts toward Universal Health Coverage (UHC). The World Health Organization (WHO) describes it as a relationship-building process that can enable stakeholders to address health challenges collectively [19]. Frameworks such as Arnstein’s ladder of citizen participation and co-production models provide conceptual foundations to assess levels of inclusion, power-sharing, and responsiveness in public policy design [20]. In this study, Arnstein’s framework is used as a conceptual lens to interpret the degree of participation and power-sharing within the engagement process. Recent guidance including the WHO handbook on social participation and the 2024 World Health Assembly resolution, suggests that embedding engagement within national health systems policy making processes can support equity, resilience, and people-centered governance [21, 22]. The Global Fund Strategy 2023–2028 also highlights the role of communities in HIV, TB, and malaria responses, with funding applications expected to involve dialogue with civil society, key populations, and people affected by the diseases [23]. The Global Fund’s community engagement strategic initiative further encourages participatory processes during grant design and implementation [24].

Nepal, a lower-middle-income country in South Asia, has some experience with bottom-up policy formulation. In practice, however consultations have tended to occur mainly from provincial up to federal level, with less frequent involvement of local government or directly affected communities [25]. The 2015 constitution of Nepal recognized health as a fundamental right and introduced a federal governance structure with decentralized service delivery. Following the 2017 elections, responsibilities for health planning, service delivery, and community engagement were shared across federal, provincial and local levels although the coordination between three tiers continues to face challenges [26–28].

Across the globe, CSE and its impact on health systems and programmatic implementation are increasingly reported in relation to specific priority diseases: HIV, TB and malaria [23]. WHO has highlighted the role of community engagement in supporting the optimization of community health worker programs in these areas [29]. In its findings from a qualitative study on community engagement in Malawi, WHO suggested that effective engagement may require early involvement, regular feedback, and active community participation [29], and that empowering service recipients can help strengthen community-based interventions for malaria elimination [2]. Studies from Brazil and Nigeria described how approaches that incorporate human rights, civil society participation, and social mobilization were associated with reductions in AIDS-related incidence and mortality [30]. In Nepal, a mixed-methods study in 2010 indicated that civil society involvement in delivering HIV and malaria interventions for high-risk groups was considered valuable [12].

Although previous literature [2, 30, 31] has outlined potential benefits of CSE, it also indicates that approaches are influenced by the social, cultural and political context of the state. In Nepal, many health policies have been developed largely by experts, with limited (or minimum) consultation at community level [32, 33]. The use of a nationwide, multi-tiered CSE process for shaping strategic priorities in HIV, TB and Malaria programs represents a relatively new approach for the country. At the time of this study, there had been no systematic assessment of such engagement in national programs, particularly in relation to its possible contributions to policies, strategies, and implementation across federal, provincial and local levels. Conducting the process during the COVID-19 pandemic further provides insights that are relevant for future planning including during adverse circumstances.

In Nepal, the Country Coordinating Mechanism (CCM) serves as a national multi-stakeholder platform responsible for coordinating Global Fund supported programs and facilitating an inclusive country dialogue process. Established in 2002, the CCM brings together representatives from government, civil society, key populations, development partners, and the private sector to promote transparency, participation, and country ownership in health program planning and implementation. In the context of this study, the CCM played a central role in convening stakeholders across federal, provincial, and local levels and supporting the broader community and stakeholder engagement process.

The main objective of this study was to collaboratively explore, together with the community partners, the community and stakeholder engagement processes in national priority setting for TB, HIV and malaria.

## Materials and methods

### Study design and methodological orientation

This study adopted a qualitative case study design, following the Guidance for Reporting Involvement of Patients and the Public (GRIPP2) checklist [34] to ensure comprehensive and transparent reporting of qualitative methods and stakeholder involvement in the research process (Supplementary File 1). Following GRIPP2 reporting guidance, we documented how affected communities and public contributors were engaged as active research partners throughout the study. Their involvement included all stages of the research process, from co-design of tools to data generation, analysis, and validation.

The case study approach allowed for in-depth exploration of the Community and Stakeholder Engagement (CSE) processes that informed Nepal’s National Strategic Plans (NSPs) for HIV, (Supplementary File 2), TB (Supplementary File 3), and malaria (Supplementary File 4) for the 2021–2024 Global Fund grant cycle (Supplementary Files 2–4). The sampling strategy is described separately in Supplementary File 5 (National Consultation Process Stakeholder Engagement and Sampling Strategy).

The research was grounded in a constructivist epistemological approach, recognizing that knowledge is co-produced through dialogue among communities, technical experts, and policymakers, thereby building an epistemic foundation rooted in community perspectives [35].

This study is best understood as a participatory qualitative case study of a large-scale community and stakeholder engagement (CSE) process conducted for national priority setting. While the engagement incorporated elements of collaboration and co-production, such as involvement of community representatives in data collection, interpretation, and validation, it did not constitute fully community-led research, as decision-making authority remained embedded within formal institutional structures. Compared to traditional consultation approaches, the process supported deeper participation through iterative feedback and shared interpretation, while remaining distinct from community-based participatory research or action research, which typically involve greater community control over research design and implementation.

The methodological framework was further informed by the Global Fund’s modular structure, principles of participatory governance, and key contextual factors including Nepal’s transition to federal governance and the COVID-19 pandemic [23, 36]. Engagement activities included a mix of group discussions, consultations and workshops conducted at local, provincial and national levels. A hybrid model combining in-person and virtual modalities was used to maintain engagement during the pandemic, supporting inclusive participation and continuity of consultations despite pandemic related restrictions. Community representatives contributed to the development of discussion guides and, where feasible, supported facilitation and documentation of sessions, including co-facilitation of local sessions and documentation of participant inputs as part of the research process. The CCM secretariat supported the coordination and facilitation of stakeholder engagement activities throughout the study.

### Participatory engagement process and analytical approach

This study involved a nationwide participatory engagement process conducted to inform priority setting for HIV, tuberculosis and malaria programs. This process included community group discussions, key informant interviews, consultation meetings, workshops and document reviews conducted across federal, provincial and local levels.

While these activities generated data for national priority setting, the primary objective of this study was to analyze the community and stakeholder engagement process itself. Data used for this analysis were derived from a purposively selected subset of qualitative data generated during these activities. Specifically, data for process evaluation included: (i) selected transcripts from community group discussions (CGDs) and key informant interviews (KIIs), (ii) observation notes from multi-stakeholder workshops, and (iii) consultation and validation records documenting participation dynamics and stakeholder interactions.

Data for priority setting and process evaluation were collected concurrently; however, only information-rich data relevant to understanding the engagement process (e.g., inclusiveness, participation dynamics, transparency and perceived influence) were included in the analytical dataset. A subset of 30 transcripts was selected for in-depth thematic analysis, while additional data sources (observation notes, consultation records, and document reviews) were used for supplementation and validation.

### Research team and reflexivity

The research team consisted of three members: two senior officials from the CCM Secretariat (SN and RS) with public health expertise, who were directly involved in planning and facilitating the CSE process, and one independent researcher affiliated with national and international academic institutions (BA). This external researcher brought experience in qualitative research, community engagement, public health policy, and health systems governance.

Given the dual role of CCM-affiliated members as both facilitators and researchers, the team applied continuous reflexivity to minimize potential bias. This included regular team discussion, included weekly debrief sessions to reflect on positionality, interpretation, assumptions, and emerging insights; reflexive journaling by each analyst; and validation of findings through multi-stakeholder consultations. These practices supported credibility and balance in data interpretation while leveraging the contextual expertise of CCM members. 

In addition, community co-researchers, also co-authors in this study (civil society representatives: AS, and UK) also contributed to reviewing coding summaries and interpretating findings, helping ensure alignment with lived experiences.

### Study setting and context

The study was conducted between January and June 2020 across all seven provinces and 72 local municipalities of Nepal. This period coincided with the development of National Strategic Plans (NSPs), preparation of the Global Fund Grant Cycle 6 application, Nepal’s transition to a federal governance system and the onset of the COVID-19 pandemic, with a national lockdown imposed on 24 March 2020 [37, 38]. The engagement process was implemented in a phased manner across federal, provincial, and local levels. National-level consultations were conducted in January 2020, followed by provincial-level consultations, key informant interviews, and group discussions in February. In March, consultations were extended to municipal and rural municipality levels.

Following the lockdown, engagement activities transitioned to virtual platforms (e.g., Zoom, Google Meet and WhatsApp) to ensure continuity. From April to June 2020, consultations, validation discussions and prioritization exercises were conducted virtually at provincial and federal levels. Across all phases, community discussions, focus group discussions and key informant interviews were conducted iteratively to support continuous engagement and triangulation of perspectives.

### Sampling and participant recruitment

A purposive sampling strategy was used to ensure representation across key stakeholder groups, with particular emphasis on historically marginalized populations (Supplementary File 5: National Consultation Process Stakeholder Engagement and Sampling Strategy). This was complemented by snowball sampling to reach additional grassroots and hard-to-reach participants through civil society and community networks.

Participants were grouped into five categories (Table 1). (1) community members and key populations (e.g., people living with HIV, TB-affected individual, migrants, sex workers, MSM, transgender people, people who inject drug, prisoners and community in malaria endemic areas, including female community health volunteers); (2) government officials at federal, provincial and local levels; (3) development partners; (4) healthcare providers, researchers and academics; and (5) civil society leaders and human rights representatives.

**Table 1:** Summary of stakeholder participation and analytical representation

Stakeholder group	Participation in engagement process	Representation in analytical dataset
Community & key populations	Majority (~85%)	Included in selected transcripts
Government (federal, provincial, local)	High participation	Included
Civil society/NGOs	High participation	Included
Development partners	Moderate participation	Included
Researchers/academia/providers	Moderate participation	Included

Participants were selected based on their direct involvement in or influence over HIV, TB, and Malaria programs. Selection was guided by structured stakeholder mapping conducted by the CCM secretariat in collaboration with government and partner networks. Invitations were issued through formal channels, including official letters to government entities and coordinated outreach through civil society platforms. Selection was purposive, aiming to ensure balanced representation across stakeholder groups and governance levels. To minimize selection bias and avoid overrepresentation of more vocal or institutionally influential actors, deliberate efforts were made to prioritize marginalized and key populations. Snowball sampling was used selectively to include participants not captured through formal networks.

While this approach enabled broad participation, the process relied partly on existing institutional and community networks. As a result, individuals outside these networks or with limited digital access during COVID-19 may have been underrepresented.

Given the nationwide scope of engagement, diverse perspectives were captured across stakeholder groups, with recurring themes consistently identified. The scale of engagement exceeded conventional expectations of data saturation [39].

### Stakeholder sensitization process

**Fig. 1:**
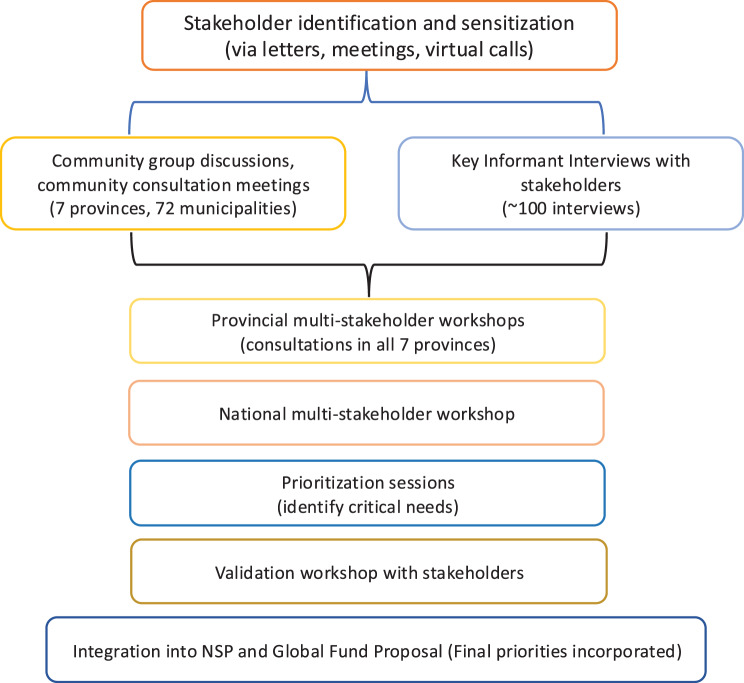
Stakeholder engagement process for HIV, TB, and malaria strategy development in Nepal (2020)

Prior to initiating community group discussions, consultation meetings and multi-stakeholder engagements, stakeholders were systematically sensitized to support meaningful participation in the engagement process (Fig. 1). Sensitization was conducted through a combination of virtual meetings, formal letters (for government agencies), and email communications (for civil society, key populations, and development partners).

These activities introduced stakeholders to the objectives, timeline, and methodology of the engagement process, and clarified how their inputs would contribute to the National Strategic Plans (NSPs) and the Global Fund application. In the context of COVID-19, virtual platforms such as Zoom and Google Meet were used to engage stakeholders across federal, provincial, and local levels (Supplementary File 6). Stakeholders were introduced to the objectives, timeline, and methodology of the engagement process prior to consultations and were invited through a combination of formal government communication (official letters) and structured outreach through civil society and partner networks, ensuring transparent and systematic inclusion.

### Data collection procedures

The study employed a multi-method qualitative approach with triangulation across multiple data sources. In line with the principles of public involvement in research, community representatives contributed as co-facilitators and documenters.

Data were collected using five complementary methods. First, a total of 700 community group discussions were conducted with 2,062 participants across all seven provinces representing diverse stakeholder groups and geographic locations. These discussions were primarily organized at ward and municipal levels to ensure broad participation from key populations, local health workers and community leaders. Semi-structured discussion guides were used to explore barriers to services, health system gaps, human rights issues and experiences of stigma. Some sessions were conducted as multi-stakeholder discussions depending on the needs and suggestions by the participants. Each discussion lasted between 60 and 90 minutes, was audio recorded with participant consent and supported by detailed notetaking.

Second, a total of 100 key informant interviews were conducted with policymakers, technical experts, civil society leaders, and development partners. These interviews explored governance, policy and strategic issues related to HIV, TB, and malaria programs. Interviews were conducted by research team and trained community co-researchers including civil society representatives affiliated with CCM. Most interviews were conducted virtually using secure platforms and lasted between 45 and 60 minutes.

Third, a total of 20 consultation meetings were conducted, including seven provincial-level workshops (one in each province) and thirteen national-level consultations. These consultations facilitated thematic discussions, prioritization of interventions, and validation of findings bringing together stakeholders from government, civil society, communities, academia, private sector and development partners. All sessions were systematically documented.

Fourth, participant observation was undertaken in 12 large-scale workshops using structured observation guides. These workshops included technical working group meetings, national strategic plan steering committee meetings, prioritization workshops and validation workshops for TB, HIV and malaria. Some workshops were conducted as integrated cross-cutting sessions (e.g., RSSH and supply chain). Observations documented participation dynamics, representation, and stakeholders’ interactions.

Finally, a review of over 30 policy and programmatic documents was undertaken as a part of national priority setting exercise on TB, HIV and malaria. Documents were selected purposively based on their relevance to national strategic planning and Global Fund processes. These included NSPs, disease-specific guidelines, Global Fund proposal materials, and documents related to Nepal’s COVID-19 response. The review mainly covered documents developed or in use between 2019 and 2021. This was not intended to be an exhaustive review but focused on key documents relevant to the priority-setting process. The document review added contextual richness and helped triangulate findings from the community group discussions, KIIs, and workshops. The use of multiple data sources enabled triangulation across community, stakeholders and policy perspectives, strengthening the validity and comprehensiveness of the findings.

### Prioritization, validation, and endorsement process

Findings from community group discussions, consultation meetings, key informant interviews and multi-stakeholder workshops were synthesized and shared with stakeholders through structured review sessions. Prioritization was guided by criteria such as relevance, feasibility, urgency and alignment with national program goals and available resources, following principles of stakeholder analysis [36].

A participatory approach was applied in which stakeholders ranked and refined interventions through facilitated discussions and collaborative exercises. Where differing priorities or disagreements emerged among stakeholders, these were discussed openly and resolved through consensus-based ranking, iterative dialogue and facilitated discussions to ensure balanced participation across stakeholder groups. Validation workshops provided opportunity for participants from community, provincial and national levels to review and confirm the proposed priorities. Final priorities were formally endorsed by stakeholders as part of the strategy development process.

### Data management and analysis

Audio-recorded community group discussions, consultation meetings and key Informant Interviews (KIIs) were transcribed verbatim and translated into English as required.

Given the largest dataset (700 community discussions and 100 KIIS), a purposive, maximum-variation sampling approach was applied to select thirty rich transcripts for in-depth thematic analysis. Selection ensured representation across stakeholder groups, levels of governance (local, provincial, federal) and disease areas while prioritizing information-rich transcripts. The remaining transcripts were systematically reviewed through summary matrices and consultation records to triangulate findings and validate emerging themes across the dataset. A hybrid coding approach was applied combining deductive codes (based on Global Fund’s programmatic modules) and inductive codes derived from the data. Coding and interpretation were conducted collaboratively by three researchers (Supplementary File 7 and 8), with input from community co-researchers to ensure alignment with lived experience. Selected transcripts (*n* = 30) were coded using QSR NVivo qualitative data management software, alongside Microsoft Word, and were later organised into Excel spreadsheets. Inter-coder reliability was ensured through 20% of transcripts (SN, RS and BA). Where coding discrepancies or divergent interpretations arose, these were discussed among the research team and resolved through consensus, with reference to original transcripts. Where differences reflected stakeholder perspectives rather than analytical disagreement, these were retained and interpreted as part of findings. 

### Ethical considerations

**Fig. 2:**
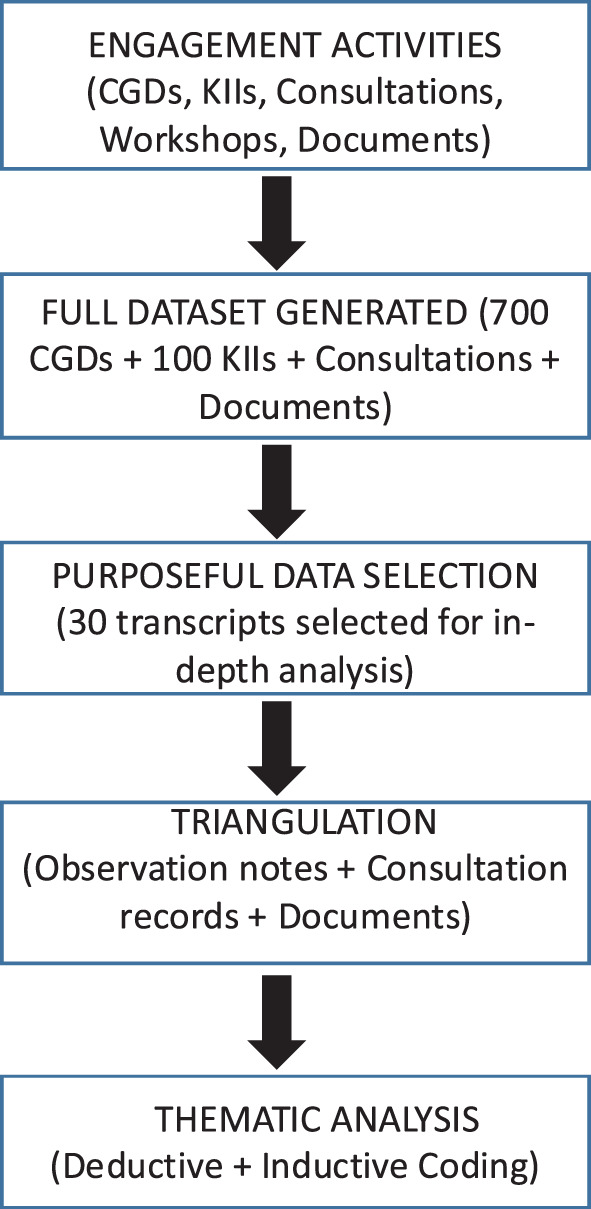
Linkage between priority-setting process and analytical dataset

The study received ethical approval from the Nepal Health Research Council (NHRC Reference # 63/2021). Informed verbal consent was obtained from all participants. For interviews conducted virtually, written informed consent was obtained electronically by sharing consent forms via email or messaging platforms, with participants providing written confirmation or returning signed forms prior to participation. All virtual interviews were audio-recorded only after obtaining verbal consent at the start of each session. Confidentiality and anonymization procedures were strictly followed, and data were stored securely on password-protected systems. A flow diagram (Fig. 2) illustrates the relationship between data collection for priority setting and the subset of data used for analysis of the engagement process.

## Results

The findings from the community and stakeholder engagement (CSE) process are presented under five thematic areas (Table 2).

**Table 2:** Summary of key themes from the engagement process

Theme	Description	Example quote
Broad inclusion of stakeholders	Engagement enabled participation across diverse stakeholders, especially key and vulnerable populations	“For the first time, I felt our experience was shaping the evidence, not just informing it,” shared one co-researcher.
Evidence-based policy and integration of lived experiences	Combining epidemiological data with lived experience improved relevance of priorities	“This has greatly helped bridge the policy and practice gaps, and explored the distinct realities of health services, budget provisions and other critical enablers as well as barriers to address the health equity issues at the communities.”
Transparency and trust	Open dialogue strengthened transparency, trust, and accountability	“The CSE process built transparency and coordination among development partners, which allowed us to align technical support more effectively. It bridged gaps between what community’s experience and what policy intends to deliver.”
Country ownership and accountability	Engagement enhanced domestic ownership and commitment, including financing	“Our Palika [municipality] has committed to allocate budget for HIV and TB medicines. This decision came after hearing directly from affected groups during the consultation. It showed us why local governments must not wait for donors but act with our own resources.”
Reinforcing resilient health system	Stakeholder inputs contributed to broader system strengthening	“For the first time, our local-level feedback on drug stock outs and diagnostic delays informed national supply chain reforms. The CSE process empowered us to speak up and contribute to solutions that were eventually implemented.”

These themes reflect participants’ experiences and perspectives on engagement during national priority setting for HIV, tuberculosis and malaria programs in Nepal.

The results are based on qualitative data from community group discussions, key informant interviews and multi-stakeholder consultations conducted across different levels of the health system. Participants included community members, key and vulnerable populations, government officials, civil society representatives, development partners and service providers.

Findings were triangulated across multiple data sources, including community group discussions, key informant interviews, consultation workshops, participant observations, and document review. Across these sources, similar themes were identified, particularly related to inclusion of marginalized groups, integration of lived experiences, and system-level challenges such as stigma and access to services. While perspectives varied across stakeholder groups, the overall findings were broadly consistent (Table 3).

**Table 3:** Contribution of data sources to key findings

Data source	Contribution to findings
Community group discussions	Community experiences, barriers, lived realities
Key informant interviews	Policy, governance, and system-level perspectives
Consultation workshops	Validation and prioritization of findings
Participant observations	Participation dynamics and stakeholder interactions
Document review	Alignment with policies and strategic documents

Direct quotations are labeled by data source, indicating whether they were drawn from key informant interviews (KII) or community group discussions (CGD). The themes presented below summarize participants’ views on how the engagement process was conducted and how they experienced their involvement. Across themes, participants also described procedural features, including deliberate inclusion of marginalized groups, validation of findings through consultations and consistency of issues identified across multiple data sources. Participants also appreciated clear consent processes and safe spaces for sharing sensitive experiences, particularly in discussions related to stigma and access to services. Participants described the CSE process as going beyond a technical exercise to inform HIV, TB, and malaria strategies. They noted that it provided a platform to incorporate community and stakeholder perspectives into national planning. More than a consultation, it functioned as a platform where evidence, lived experience, and multi-tier governance intersected.

Conducted during Nepal’s transition to federalism and the COVID-19 pandemic, the process remained systematic, with validation and endorsement mechanisms reinforcing accountability. Community priorities were elevated from local dialogues into the National Strategic Plans and Global Fund proposals. The CCM played a pivotal convening role, moving beyond its grant oversight mandate to act as a broker of trust across federal, provincial, and local levels. By doing so, it reinforced the principle of country ownership and showed how national platforms can institutionalize equity and transparency in decision-making. The process demonstrated how participatory engagement can contribute to policy development, stakeholder relationships and health system strengthening. Broad inclusion of stakeholders in policy and research.

Community representative as co-authors reflected that their involvement in the CSE process was transformative, shifting their roles from being consulted participants to becoming partners in both research and CSE processes. They described learning to conduct community-based meetings, data collection process, analysis and report preparation. Several described the process as the first instance in which they were invited to co-design tools, co-facilitate sessions and verify how lived experience was represented in the outputs.

*For the first time, I felt our experience was shaping the evidence, not just informing it,”* shared one co-researcher.

(KII, community co-researcher, disease-specific network)

Another participant who took part in the entire research cycle appreciated how their inputs went through several iterative processes of interpretation, discussion, and eventual inclusion in the report. Having contributed lived experience, particularly seeing how shared challenges were incorporated into the research and policy outputs—was highly valued.

*Working alongside researchers showed us that our stories are data—our lived realities have value in national policy. *

(KII, community co-researcher, disease-specific network)

Community co-researchers also highlighted practical limits: digital access constraints during lockdowns, uneven opportunity to participate across provinces, and the need for dedicated time and modest resources to sustain involvement beyond funding cycles. Nonetheless, they perceived tangible influence on priorities (e.g., stigma reduction in TB care, integrated TB/HIV diagnostics, and community-level surveillance), which increased their trust in the process and sense of joint ownership.

Civil society leaders explained that the entire process was itself a form of capacity building. They learned to interpret budgets, analyze indicators, and identify policy gaps, which strengthened their ability to advocate in future dialogues. Indeed, participation at every stage of the CSE process enabled them to gain valuable capacity-building experience.

*Through this process, our networks learned how to interpret budgets, analyze indicators, and even challenge policy gaps constructively. It was capacity building in action.*

(KII, 32-year-old female PLHIV Network representative)

The CSE process was seen as generating “CSE capital.” This referred to the collective knowledge and capacity that stakeholders built through the process, which apparently equipped them to engage more meaningfully in policymaking and implementation going forward.

Community co-researchers viewed the engagement as an opportunity to experience the capacity building in reality, enhancing their research literacy, legitimizing community knowledge, and reinforcing accountability between communities and policymakers. They recommended institutionalizing structured roles for community partners in future evidence generation, analysis, and priority setting.

Apart from the community representatives participating in research following the CSE, the wide representation from federal, provincial, and local levels were valued by most participants. This inclusive approach reflected Nepal’s health context, national health policies, and disease-specific goals. By engaging diverse actors at different tiers of governance, the process created space for alignment between community priorities and national frameworks. This enhanced the relevance of the national strategic plans and ensured that government and stakeholders were better positioned to contribute to planning, implementation, and monitoring and evaluation of HIV, TB, and Malaria strategies.

The consultations included meaningful input from key and vulnerable populations such as PLHIV, TB survivors, PWID, FSWs, MSM, transgender people, communities in malaria-affected areas, and other vulnerable populations. Stakeholders emphasized that their lived experiences and recommendations were visibly incorporated into the final strategic documents. Approximately 85% of those who participated in priority-setting activities were from key or vulnerable populations, emphasizing on the inclusiveness of the process.

Participants also noted that targeted stakeholder mapping and outreach helped reduce overrepresentation of more vocal or institutional actors, although some challenges remained for individuals with limited digital access during COVID-19. Many participants described how valued they felt when their recommendations were included in official policy. The experience of reviewing a final strategy that reflected community voices gave them confidence that their engagement had been meaningful, more than symbolic as was the case in the past.

*During the process of development of the National Strategic Plan for HIV, TB and malaria - a series of consultations were held. And when I saw the final version of the strategic plan, I was happy to see that the central priorities suggested by the key and vulnerable populations as well as the affected communities during the consultation were given high importance in the strategic plans.*

(KII, 43-year-old male Policymaker, MoHP)

Participants from malaria-prone areas and TB-vulnerable communities described that, their local concerns were acknowledged. They felt that the process gave visibility to rural villages, informal settlements, and minority communities that had historically been overlooked in national planning.

*In our malaria-affected village, health posts often run out of bed nets and medicines. This time, when we spoke about it in the consultation, we later saw those issues appear in the national strategy. It gave us confidence that even voices from remote areas matter.*

(CGD participant, 42-year-old male, malaria-prone district, Kanchanpur)

The findings suggest that stakeholders, particularly people living with diseases and other vulnerable groups, felt meaningfully involved and that their perspectives were taken seriously. Planning forums allowed community representatives to share needs, experiences, and programmatic recommendations directly with government officials. These inputs were ultimately integrated into national strategies.

For many participants, this was the first time their voices shaped decision-making in such a direct way. They described the process as more responsive than in the past, with government and implementing partners following up on points raised during the consultations.

*This country dialogue process provided an important opportunity to have direct interface (government–CSOs–key populations) by raising voices of communities, lobbying and advocacy to service providers for responding to the needs and priorities of HIV, TB and Malaria services across the country.*

(CGD participant, 37-year-old female PWID representative)

Unlike previous forums that mainly engaged government or civil society, this process deliberately brought in private sector actors. This broadened the perspectives available to planners, particularly around diagnostics and service access.

*The consultations created space for non-traditional partners like the private health sector to engage meaningfully. Our insights on diagnostics and treatment access were welcomed, which was rare in prior planning cycles.*

(KII, 58-year-old male, private diagnostic network representative)

The CSE also integrated equity and gender responsiveness through careful stakeholder mapping and targeted outreach. Women, LGBTQIA+ individuals, persons with disabilities, *dalit* communities, and representatives from remote regions were actively identified and mobilized through civil society networks. Representation quotas and tailored invitations ensured that marginalized voices were not only articulated but also carried influence in the final strategies.

Frontline workers such as female community health volunteers (FCHVs) highlighted that their participation was not symbolic. Their knowledge from years of working in local communities was taken on board and translated into action points.

*As a FCHV, I had never imagined my concerns about TB screening in slum areas would be included in national plans. The consultation gave us space to share what we see every day, and it felt like our work was valued.*

(CGD participant, 36-year-old FCHV, Kathmandu valley)

Participants also reported that issues such as stigma, gender barriers and access challenges were reflected in the national strategies, demonstrating policy influence.

*We felt genuinely represented, not just invited. Our issues were part of the final recommendations, especially around female-led ART delivery and community safety for transgender individuals.*

(KII, 34-year-old female, civil society leader)

The inclusive approach was applied in both CSE for policy formulation and its later process of research procedure. Participants further noted that their inputs were tracked and validated through iterative consultations and feedback process, with strengthened confidence in how their prospective were interpreted and incorporated.

### Evidence-based policy and integration of lived experiences

Respondents consistently highlighted that evidence was central to shaping the national strategies. Policymakers drew on epidemiological surveillance data, annual reports, programmatic reviews, and evaluations. Yet participants also stressed that these technical inputs were strengthened when combined with lived experiences and grassroots insights. By integrating data with the realities of affected communities and service providers, the process made national priorities more relevant and actionable. Participants also noted that the use of multiple data sources- including community discussions, interviews, and consultations allowed cross-verification of issues, strengthening confidence in the consistency and credibility of identified priorities.

Stakeholders noted that this integration bridged the traditional gap between policy design and service delivery realities. They emphasized that the process helped uncover enablers and barriers that had often been overlooked in top-down planning.

*This has greatly helped bridge the policy and practice gaps, and explored the distinct realities of health services, budget provisions and other critical enablers as well as barriers to address the health equity issues at the communities.*

(KII, 46-year-old male Policymaker, Gandaki Province)

Academics reflected that the participatory approach enabled real-time feedback and dialogue around epidemiological data. They saw this as a new way of working where planners did not just review the data but actively co-developed solutions grounded in both evidence and community realities.

*The participatory process enabled real-time feedback on epidemiological data and created demand for its application in local-level planning. For the first time, we saw planners co-developing solutions grounded in both data and local realities.*

(KII, 43-year-old male Researcher, Tribhuvan University)

(KII, 43-year-old male researcher, Tribhuvan University)

Stakeholders from religious and marginalized settlements, such as Muslim communities and monastic  areas, shared local realities that complemented epidemiological data. Their testimonies grounded the statistics with lived experience of service barriers. Participants emphasized that these discussions were conducted in environments that encouraged open and safe sharing of sensitive experiences, particularly related to stigma and access barriers.

*In our Muslim settlement, many women hesitate to visit TB clinics due to stigma. Sharing this openly in front of policymakers was powerful, because it helped them see why numbers alone cannot explain the real challenges.*

(CGD participant, 40-year-old female, Madesh Province)

Respondents also pointed out that referencing global and regional research alongside national data helped ensure interventions were evidence-informed but still sensitive to local priorities. Policymakers said that the process helped build their capacity to think more holistically. They developed a better grasp of disease burden, service delivery gaps, resource allocation, and priority actions needed to achieve national targets.

*Stakeholders’ capacity has greatly increased in analysis of the disease burdens, critical service delivery gaps and coverage, resource needs and priority actions to achieve the national targets and objectives. This is now a guiding reference to develop annual plans for cost-effective health interventions in order to end the epidemics.*

(KII, 47-year-old male Policymaker, National Planning Commission)

### Transparency and trust

Many respondents emphasized that the CSE process improved transparency in policymaking. Open dialogues created opportunities for participants to access and understand information about disease burden, resource allocations, and gaps in interventions. These exchanges helped participants feel included in decision-making and fostered a sense of shared accountability.

Participants also highlighted that iterative consultations and feedback mechanisms allowed them to review and confirm how their inputs were interpreted, reinforcing the transparency of the process. Government representatives explained that the CSE forums made roles and expectations clearer, while also encouraging candid discussion and collaboration. They felt this strengthened trust among stakeholders and reinforced a culture of openness.

*Through the CSE approach, the dialogues between community and policy makers have become much more visible in terms of their roles, contributions, and potential partnerships for creating synergies and improved outcomes. It has further enhanced information sharing and nurtured a culture of open discussion and argument that helps ensure clarity and transparency in policy and strategic decision-making.*

(KII, 55-year-old female Policymaker, DoHS)

Civil society participants highlighted that the process allowed them to identify gaps in policy and programs in a more participatory and transparent manner. This stood in contrast to earlier experiences where such processes had felt more closed or one-sided.

*The overall processes of consultations helped to ensure a participatory decision-making process in identifying policy and programmatic gaps in a more transparent manner.*

(CGD participant, 38-year-old male, TB and HIV survivor)

Development partners also saw the CSE process as a way of improving alignment. They felt that by sharing information and coordinating more openly, technical support could be better matched to community needs and policy priorities.

*The CSE process built transparency and coordination among development partners, which allowed us to align technical support more effectively. It bridged gaps between what community’s experience and what policy intends to deliver.*

(KII, 50-year-old female Technical Officer, WHO Nepal)

Local leaders, such as mayors and rural municipality chairpersons, emphasized that the CSE created a new kind of accountability. They valued being part of open forums where they could both commit to action and be challenged by communities.

*For the first time as an elective representative, I had to sit in front of community members, civil society, and national officials to explain what our municipality is doing for HIV, malaria and TB. It created a sense of joint responsibility and also motivated us to do better.*

*(KII, 51-year-old male, rural municipality chairperson),*

These reflections demonstrate that transparency was not only about making information available, it also meant clarifying roles, enabling joint problem-solving, and building trust across government, civil society, communities, and development partners. Participants further noted that the inclusive structure of discussions helped ensure that diverse perspectives, including those of marginalized groups were represented, reducing the dominance of more powerful stakeholders.

### Country ownership and accountability

Respondents widely felt that the CSE process strengthened Nepal’s sense of ownership and mutual accountability in shaping national disease strategies. By convening stakeholders from federal, provincial, and local levels, the process clarified responsibilities, reduced fragmentation, and improved coordination across institutions. Participants noted that this went beyond technical planning; it demonstrated that national actors could collectively steer priorities and mobilize resources.

Participants also emphasized that the inclusive nature of these discussions- bringing together community members, civil society, and government actors- helped ensure that decision-making reflected a broader range of perspectives, reducing the influence of more dominant stakeholders. A major theme in this area was financing. For many, the CSE created an unprecedented space to discuss domestic contributions and resource mobilization openly. Stakeholders explained that these conversations helped reduce reliance on external donors and strengthened national stewardship of HIV, TB, and malaria responses.

The open and participatory nature of these discussions also allowed stakeholders to observe and verify commitments made during consultations, reinforcing transparency and accountability.

*The CSE approach provided an opportunity to discuss and reprioritize resources for alignment among partners to address the immediate critical health care needs of key and vulnerable populations.*

(KII48-year-old male, Principal Recipient representative)

Development partners pointed out that these transparent discussions, especially when communities were present, created new forms of accountability. They observed that national commitments were no longer abstract promises, but public pledges shared in front of the very groups most affected by the diseases.

*By ensuring that domestic financing and co-financing commitments were openly discussed with communities, the process advanced both transparency and accountability. We saw stronger local stewardship emerge.*

(KII, 50-year-old female, USAID Nepal)

Municipal leaders noted that being part of the dialogue gave them clarity about their financing role. They also pledged to allocate local funds for disease programs, which reinforced the principle of shared responsibility.

*Our Palika [municipality] has committed to allocate budget for HIV and TB medicines. This decision came after hearing directly from affected groups during the consultation. It showed us why local governments must not wait for donors but act with our own resources.*

(KII, 45-year-old female municipality vice chair)

Civil society groups explained that the technical knowledge they gained through the CSE, such as on budget analysis and indicator monitoring, positioned them to hold decision-makers more accountable. Policymakers also admitted that being confronted with community perspectives in open forums made them feel a greater sense of responsibility for their commitments. Participants further noted that this process strengthened accountability by linking community feedback directly with policy and financing decisions, enhancing confidence in the integrity of commitments made.

### Reinforcing resilient health system

Respondents emphasized that the CSE process was not limited to identifying priorities for HIV, TB, and malaria programs. It also acted as a platform to strengthen health system resilience more broadly. Stakeholders pointed out that through discussions on procurement, supply management, laboratory services, human resources, and health information systems, community voices were able to directly influence systemic reforms.

Participants also noted that recurring issues identified across community discussions, interviews, and consultations reinforced confidence that priorities were consistently validated across multiple sources. Participants reported that their feedback on recurring challenges such as drug stock outs and diagnostic delays shaped supply chain reforms. This was the first time many felt their observations at the local level had been directly linked to national solutions.

*For the first time, our local-level feedback on drug stock outs and diagnostic delays informed national supply chain reforms. The CSE process empowered us to speak up and contribute to solutions that were eventually implemented.*

(CGD participant,48-year-old female, civil society leader)

Frontline volunteers also noted that they could report challenges in real time, even during the pandemic, and saw them addressed.

*I reported how pregnant women in my ward struggled because medicines were often out of stock. Later I saw this problem being discussed at national level. It was the first time I felt our frontline experience could change the system.*

(CGD participant, 39-year-old female FCHV, Karnali province)

Participants highlighted the systematic design of the process itself. By moving from problem identification to prioritization, validation, and endorsement, the CSE ensured that inputs were not lost along the way. This structure enhanced legitimacy and promoted stakeholders’ confidence that their contributions had real weight. Participants further emphasized that this structured process helped reduce selective interpretation of inputs and ensured that diverse perspectives were systematically considered.

*The strength of the Nepal [CSE] process was how systematically they moved from problem identification to prioritization and validation. As partners, we were invited not just to review, but to help weigh trade-offs realistically.*

(KII 49-year-olds male, Implementing partner, USAID PEPFAR)

The importance of adaptability was particularly evident during the COVID-19 pandemic. Despite lockdowns, consultations quickly shifted online, enabling continuation of participation from a wider range of stakeholders and turning the CSE into an even more inclusive platform. Participants also acknowledged that while virtual platforms improved reach, some limitations remained for individuals with restricted digital access. Technical partners emphasized that this flexibility transformed the platform from consultation into joint planning, allowing technical support to be more responsive to real-time system gaps raised by communities and health workers. Academics highlighted that the process created a rare but direct knowledge-to-policy loop. Findings from operational research, such as those on human resource shortages and laboratory delays, were integrated directly into national strategies instead of being left on the margins.

*This process allowed evidence from our operational research on human resource shortages and lab delays to be integrated directly into national Procurement and supply Chain management (PSM) strategies. That kind of knowledge-to-policy feedback loop was rare before.*

(KII, 46-year-old female Researcher, NHRC)

Private sector actors observed that their contributions were formally recognized in maintaining service continuity during the pandemic. They valued being consulted on procurement and testing coverage, areas where they had long played an informal but important role.

*Being invited to discussions on procurement and diagnostic coverage allowed us to offer ideas for decentralized testing. We appreciated the openness and transparency, something we hadn’t seen in earlier planning cycles.*

(KII, 50-year-old male, private diagnostics provider)

Global health partners described Nepal’s CSE processes as a useful example of inclusive engagement. Participants noted that the continuation of nationwide engagement may have contributed to maintaining responsiveness. By including community voices, sustaining engagement during crises, and linking research with policy and practice, the process was perceived to support more adaptive and people-centered approaches to health governance in Nepal. While perspectives were broadly aligned across stakeholder groups in recognizing the value of inclusive engagement and trust-building, some differences were also noted across levels of governance. Participants at community and municipal levels tended to emphasize representation, local service access, and immediate programmatic needs, whereas national-level stakeholders more often referred to coordination, feasibility, and alignment with broader strategies. Development partners also highlighted alignment and efficiency. These differences appear to reflect the varying roles and priorities across governance levels.

## Discussions

Community and Stakeholder Engagement (CSE) in Nepal’s HIV, TB, and malaria programs extended beyond a conventional technical consultation process and provided a structural platform for engagement across federal, provincial and local levels during a period of federal transition and COVID-19 pandemic. The CSE processes, along with stakeholder involvement in the research cycle, demonstrated how inclusive dialogue among communities, civil society, and policy makers could contribute to shaping national strategies. Unlike previous processes, the scale of engagement was unprecedented, involving over 700 community discussion sessions and 100 key informant interviews across all seven provinces.

Our study echoes a similar level of large-scale community and public participatory research in Austria, where more than 400 citizens participated in physical activity promotion conversations [40]. The breadth of participation also aligns with global calls from WHO, the Global Fund, and UHC frameworks, to embed social participation and people-centered approaches into health policy-making [19, 21, 23]. The discussion that follows reflects on five major themes that emerged from the process: inclusion, evidence integration, transparency, ownership and health system resilience. These themes resonate with the RISE study’s observations that CCMs are strongest when they balance engagement, equipping and empowerment of all stakeholders [41]. Nepal’s practices such as validation workshops, priority-tracking, research involvement and open financing discussions, address some of the gaps identified in RISE, though sustaining participation across the grant cycle remains a challenge. Reflecting on Arnstein’s ladder of citizen participation [20], the findings may suggest that the CSE process in Nepal extended beyond consultation towards elements of involvement and partnership. Participants described opportunities to contribute to dialogue, validation, and interpretation of findings, suggesting elements of shared knowledge generation with the process. At the same time, decision-making authority appeared to remain largely within formal institutional structures, suggesting that higher levels of citizen control may not have been fully realized. These observations highlight both the progress made in participatory approaches and the structural factors that continue to shape power-sharing in national health policy processes. The approach observed in this study can be positioned between consultation and co-production, with elements of participatory governance but without full transfer of decision-making authority to communities.

Given the involvement of some researchers in the CCM and engagement process, potential bias was recognized. This was mitigated through reflexive practices, inclusion of independent analytical perspectives, and validation of findings with diverse stakeholders. Findings from this study demonstrate how community voices can be integral to both the policy formulation process and research outputs, broadly aligning with the idea that such processes enhance epistemic inclusivity in policy and research evidence generation, as often discussed in global health [9, 35, 42]. The CSE brought together a wide spectrum of voices including people living with HIV, TB survivors, female community health volunteers, key populations, and communities from malaria-endemic districts. Participants noted that their perspectives visibly materialized in the final strategic plans. Comparable studies in Brazil and Nigeria confirm that engagement of marginalized populations strengthens policy relevance and equity in HIV and TB responses [30]. WHO similarly underscores inclusivity as a cornerstone of people-centered governance [19]. For Nepal, the key implication is that intentional outreach and inclusive representation of marginalized groups are essential to addressing perceived inferiority rooted in hierarchical and power imbalances [42]. Deliberate efforts to foster such inclusivity can enhance equity in participation across the CSE process and in the production of subsequent research outputs [35].

Building on this inclusivity, the process also revealed that national strategies became more actionable when epidemiological data were combined with lived experiences of affected communities and frontline workers. This integration helped bridge policy–practice gaps, such as understanding drug stock outs or service access barriers for migrants. Similar lessons are emphasized in UHC frameworks, which highlight that health data becomes meaningful when interpreted through community realities [24]. WHO further emphasizes lived experience as valid and critical resource for evidence for health decision-making [21]. For Nepal, the key implication is to institutionalize mechanisms that systematically integrate surveillance data with grassroots perspectives, recognizing that proximity to ground realities enhances the generation of community-responsive evidence [43]. When inclusivity and evidence are combined, the outcome is more credible, generating context-sensitive policies that both communities and decision-makers can trust [44]. This also echoes with the principle of how inclusivity and governance function as vital components for a successful health system [45].

This integration of perspectives also fed into a culture of openness. Another finding was that open CSE forums fostered transparency and potentially strengthened trust among communities, government, and partners [46, 47]. Participants gained access to information on budgets, supply chain gaps, and priorities, while policymakers described greater clarity of roles, interest and accountability [36]. Similar participatory approaches in Malawi and Ghana have been associated with stronger alignment between state and community actors [29, 48]. Nepal could establish regular CSE platforms as formal accountability mechanisms. In doing so, the combination of inclusivity, evidence, and transparency can reinforce each other, creating a vicious cycle that strengthens relationships between stakeholders [46].

Local governments pledged resources for TB and HIV services after hearing community voices, signaling a shift from donor dependency to shared national responsibility. This reflects the Global Fund’s emphasis on domestic resource mobilization and accountability as critical to sustainability [23]. Comparative experiences from LMICs show that community involvement in financial discussions enhances transparency and accountability [49].

CSE process also identified and built through the feedback process on procurement bottlenecks, human resource shortages, laboratory delays, and stock outs which later were translated into national policies. Evidence from Cambodia, South Africa, and India have also echoed on how participatory approaches enhance health system resilience by creating feedback loops between communities and policymakers [2, 50, 51].

Nepal’s CSE process illustrates how participatory governance can improve both the content of national strategies and the legitimacy of policymaking. By embedding inclusivity [52], integrating evidence with lived realities, fostering transparency [53], reinforcing ownership [54], and enhancing resilience [55], CSE generated lessons relevant for global actors, for e.g. funding bodies and national health systems. This aligns with the broader principle that integrating governance and inclusivity can strengthen institutional practices and lead to outcomes that promote equity [56].

Alongside the CSE processes, including community representatives and stakeholders across the research cycle proved highly instructive for Nepal, revealing how seemingly minor issues—often overlooked in conventional research—were highlighted during data interpretation and output generation [57].

**Fig. 3:**
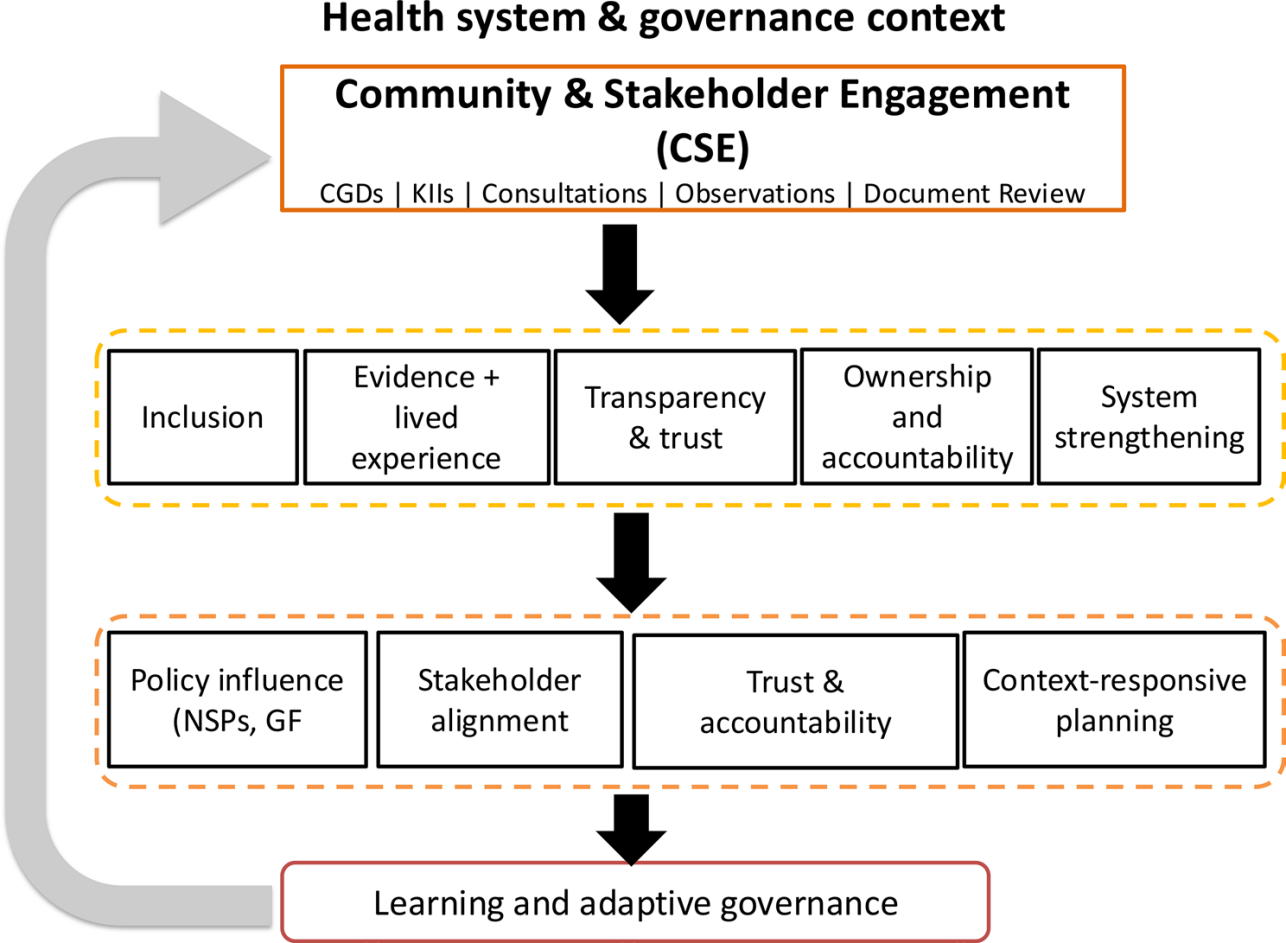
Conceptual framework of CSE for national priority setting

These findings indicate that structured community and stakeholder engagement could be considered within routine national planning processes, such as the development and revision of National Strategic Plans and Global Fund grant cycles. Incorporating similar approaches in future planning processes may help maintain continuity of stakeholder input and support alignment between community priorities and program design. Experiences from this process also provide practical insights that could inform how such approaches are adapted within existing institutional mechanisms. Conceptual framework derived from the study findings illustrate the relationships between engagement processes, key domains, and observed outcomes (Fig. 3).

### Strengths and limitations

This study analyzed a national policy-making process coordinated through CCM, thus covers a breadth of data through series of consultative processes, discussions and their integration into the grant application and policy documents. While breadth of data collection offered nationwide perspectives, the depth may have been compromised as these engagement processes were initiated largely through the government entities. Despite efforts to ensure inclusive and systematic sampling, there remains a possibility of selection bias due to reliance on existing institutional and community networks. In doing so, the process may also have suffered from some form of selection biases. The breadth of data collected in this study could not be included; thus a fair proportion was included to ensure the data management, analysis and reporting which may have inadvertently excluded the representation. Nonetheless, inclusion of documents for this study was based on the rich and detailed engagement reports. As a part of the national level CSE process, authors were largely at the policy levels or were involved in managing the entire operation, which may have used the broader and deductive lens in the results and interpretations.

## Conclusions

The CSE process in Nepal provided critical learning for future health policy planning and demonstrated the value of meaningful community involvement in research. Engaging community members as active partners in data collection, interpretation, and validation underscored the participatory nature of the entire research cycle, ensuring that priorities were grounded in community realities, broadly inclusive, and strategically aligned with institutional frameworks. The process also showed that genuine participation is possible even during crises, provided that digital access, hybrid consultation formats, and early engagement are prioritized. Moreover, the inclusion of geographically remote communities, private sector partners, and academic voices added depth and diversity to strategic dialogue. These reflections offer a model for embedding inclusive, community-engaged planning into Nepal’s health governance beyond Global Fund cycles and into routine national and subnational processes—offering transferable insights for other low- and middle-income countries.

## Electronic supplementary material

Below is the link to the electronic supplementary material.

Supplementary material 1

Supplementary material 2

Supplementary material 3

Supplementary material 4

Supplementary material 5

Supplementary material 6

Supplementary material 7

Supplementary material 8

## Data Availability

Data cannot be shared publicly because of the risk that respondents could be identified. Following the ethical guidelines of Nepal Health Research Council on data sharing policy and written informed consent with participants where we ensured the anonymity and confidentiality of the information provided to us, data cannot be shared publicly. Even when sharing the data after removing the socio-demographics of the participants, particular quotes, and the dates of study they took part in are likely to reveal their identification. However, data are available on reasonable request to Nepal Health Research Council (E-mail: nhrc@nhrc.gov.np).

## Abbreviations

AIDS: Acquired Immune Deficiency Syndrome
ART: Antiretroviral Therapy
CSE: Community and Stakeholder Engagement
CGD: Community Group Discussions
CCM: Country Coordinating Mechanism
COVID-19: Coronavirus Disease 2019
CSO: Civil Society Organization
DoHS: Department of Health Services
EDCD: Epidemiology and Disease Control Division
FCHV: Female Community Health Volunteer
FGD: Focus Group Discussion
FSW: Female Sex Worker
GoN: Government of Nepal
HIV: Human Immunodeficiency Virus
KII: Key Informant Interview
LLIN: Long-Lasting Insecticidal Net
LMIC: Low- and Middle-Income Country
MoHP: Ministry of Health and Population
MSM: Men Who Have Sex with Men
NCASC: National Centre for AIDS and STD Control
NHRC: Nepal Health Research Council
NSP: National Strategic Plan
NTCC: National Tuberculosis Control Center
NPHL: National Public Health Laboratory
PEPFAR: President’s Emergency Plan for AIDS Relief
PLHIV: People Living with HIV
PR: Principal Recipient
PSM: Procurement and Supply Management
PWID: People Who Inject Drugs
SR: Sub-Recipient
TB: Tuberculosis
TG: Transgender
UHC: Universal Health Coverage
UNAIDS: Joint United Nations Programme on HIV/AIDS
UNDP: United Nations Development Programme
USAID: United States Agency for International Development
WHO: World Health Organization

## References

[1] Adhikari B, Pell C, Cheah PY. Community engagement and ethical global health research. Global Bioethics. 2020;31(1):1–12. Epub 2020/02/01. 10.1080/11287462.2019.1703504. 32002019 10.1080/11287462.2019.1703504 PMC6968663

[2] Baltzell K, Harvard K, Hanley M, Gosling R, Chen I. What is community engagement and how can it drive malaria elimination? Case studies and stakeholder interviews. Malar J. 2019;18(1):245. 10.1186/s12936-019-2878-8. 31315631 10.1186/s12936-019-2878-8 PMC6637529

[3] Adhikari B, Pell C, Phommasone K, Soundala X, Kommarasy P, Pongvongsa T, et al. Elements of effective community engagement: lessons from a targeted malaria elimination study in Lao PDR (laos). Globalizat Health Action. 2017;10(1):1366136. Epub 2017/09/16. doi: 10.1080/16549716.2017.1366136. 10.1080/16549716.2017.1366136 PMC5645700 28914184

[4] Adhikari B, James N, Newby G, von Seidlein L, White NJ, Day NPJ, et al. Community engagement and population coverage in mass anti-malarial administrations: a systematic literature review. Malar J. 2016;15(1):523. Epub 2016/11/04. doi: 10.1186/s12936-016-1593-y. 27806717 10.1186/s12936-016-1593-y PMC5093999

[5] Tindana PO, Singh JA, Tracy CS, Upshur REG, Daar AS, Singer PA, et al. Grand challenges in global health: community engagement in research in developing countries. PLoS Med. 2007;4(9):e273. 10.1371/journal.pmed.0040273. 10.1371/journal.pmed.0040273 PMC1989740 17850178

[6] Lavery JV. Building an evidence base for stakeholder engagement. Science. 2018;361(6402):554–56. 10.1126/science.aat8429. 30093590 10.1126/science.aat8429 PMC7745122

[7] Musesengwa R, Chimbari MJ, Mukaratirwa S. A framework for community and stakeholder engagement: experiences from a multicenter study in Southern Africa. J Empirical ResearchHum Res Ethics. 2018;13(4):323–32. 10.1177/1556264618769002. 10.1177/1556264618769002 29701110

[8] Helbig N, Dawes S, Dzhusupova Z, Klievink B, Mkude CG. Stakeholder engagement in policy development: observations and lessons from international experience. In: Policy practice and digital science: integrating complex systems, social simulation and public administration in policy research. Springer; 2015. p. 177–204. 10.1007/978-3-319-12784-2_9.

[9] Hyder A, Syed S, Puvanachandra P, Bloom G, Sundaram S, Mahmood S, et al. Stakeholder analysis for health research: case studies from low- and middle-income countries. Public Health. 2010;124(3):159–66. Epub 20100312. doi: 10.1016/j.puhe.2009.12.006. 20227095 10.1016/j.puhe.2009.12.006

[10] Lemke AA, Harris-Wai JN. Stakeholder engagement in policy development: challenges and opportunities for human genomics. Genet Med. 2015;17(12):949–57. Epub 20150312. doi: 10.1038/gim.2015.8. 25764215 10.1038/gim.2015.8 PMC4567945

[11] Gilson L, Erasmus E, Borghi J, Macha J, Kamuzora P, Mtei G. Using stakeholder analysis to support moves towards universal coverage: lessons from the SHIELD project. Health Policy Plan. 2012;27(suppl 1):i64–76. 10.1093/heapol/czs007. 10.1093/heapol/czs007 22388502

[12] Tragard A, Shrestha IB. System-wide effects of Global fund investments in Nepal. Health Policy Plan. 2010;25(Suppl. 1):i58–62. 10.1093/heapol/czq061. 10.1093/heapol/czq061 20966112

[13] IAP2. IAP2’s public participation toolbox. (12th July 2025). 2018; Available online at: https://cdn.ymaws.com/www.iap2.org/resource/resmgr/pillars/Spectrum_8.5x11_Print.pdf.

[14] Kapiriri L. Stakeholder involvement in health research priority setting in low income countries: the case of Zambia. Research Involv Engagem. 2018;4(1):41. 10.1186/s40900-018-0121-3. 10.1186/s40900-018-0121-3 PMC6234591 30460042

[15] Ean M, Tripura R, Sothea P, Savoeun U, Peto TJ, Bunthynn S, et al. A youth advisory group on health and health research in rural Cambodia. Global Bioethics. 2024;35(1):2361968. 10.1080/11287462.2024.2361968. 38859929 10.1080/11287462.2024.2361968 PMC11164040

[16] Khirikoekkong N, S-A A, Osterrieder A, Lwin KM, Cheah PY. Embedding voices of under-served communities in health research priority setting in Thailand. Wellcome Open Res. 2025;9:627. 10.12688/wellcomeopenres.22940.2. 40051681 10.12688/wellcomeopenres.22940.2 PMC11883205

[17] Grill C. Involving stakeholders in research priority setting: a scoping review. Research Involv Engagem. 2021;7(1):75. 10.1186/s40900-021-00318-6. 10.1186/s40900-021-00318-6 PMC8555197 34715932

[18] Burke NN, Stewart D, Tierney T, Worrall A, Smith M, Elliott J, et al. Sharing space at the research table: exploring public and patient involvement in a methodology priority setting partnership. Research Involv Engagem. 2023;9(1):29. 10.1186/s40900-023-00438-1. 10.1186/s40900-023-00438-1 PMC10152423 37131232

[19] WHO. Technical consultation on determining non-inferiority of vector control products within an established class: report of a virtual meeting. 2021, August-2 September, 31.

[20] Story WT, Pritchard S, Hejna E, Olivas E, Sarriot E. The role of integrated community case management projects in strengthening health systems: case study analysis in Ethiopia, Malawi and Mozambique. Health Policy Plan. 2021;36(6):900–12. 10.1093/heapol/czaa177. 33930137 10.1093/heapol/czaa177

[21] WHO. Social participation for universal health coverage. World Health Organization. 2023. 11 May 2026. Available online at: https://bit.ly/42ZYHXE.

[22] WHO. Status of collection of assessed contributions, including member States in arrears in the payment of their contributions to an extent that would justify invoking article 7 of the constitution. 2021. 11 May 2026. Available online at https://bit.ly/4dgzooZ.

[23] The Global Fund. Fighting pandemics and building a healthier and more equitable World.

[24] The Global Fund. Community engagement strategic initiative. Available online at 2023. 2nd August 2025. https://bit.ly/3R3WnfF.

[25] Limbu PP. Development policy process in Nepal: a critical analysis. Int Res J Mgt Sci. 2019;4:65–82. 10.3126/irjms.v4i0.27886.

[26] Ministry of Health and Population. Nepal safe motherhood and newborn health road map 2030. MOHP: ministry of health and population. 2019. 11 May 2026, Nepal. Available online at https://bit.ly/4whHiap.

[27] Mishra SR, Ghimire K, Khanal V, Aryal D, Shrestha B, Khanal P, et al. Transforming health in Nepal: a historical and contemporary review on disease burden, health system challenges, and innovations. Health Res Policy Sys. 2025;23(1):61. Epub 20250520. doi: 10.1186/s12961-025-01321-z. 10.1186/s12961-025-01321-z PMC12090584 40394610

[28] Adhikari B, Mishra SR, Schwarz R. Transforming Nepal’s primary health care delivery system in global health era: addressing historical and current implementation challenges. Global Health. 2022;18(1):8. Epub 2022/02/02. doi: 10.1186/s12992-022-00798-5. 35101073 10.1186/s12992-022-00798-5 PMC8802254

[29] WHO. Health policy and system support to optimize community health worker programmes for HIV, TB and malaria services: an evidence guide. Health policy and system support to optimize community health worker programmes for HIV, TB and malaria services: an evidence guide2020. 11 May 2026. Available online at https://bit.ly/4eB1gXm.

[30] Schoppmeyer ES. Gender sexuality and human rights: a comparative analysis of the role of civil society organizations in HIV/AIDS responses in Brazil and Nigeria. 2021. 11 May 2026. Available online at https://bit.ly/48VuGvI.

[31] Citro B, Soltan V, Malar J, Katlholo T, Smyth C, Sari AH, et al. Building the evidence for a rights-based, people-centered, gender-transformative tuberculosis response: an analysis of the stop TB partnership community, rights, and gender tuberculosis assessment. Health Hum Rights. 2021;23(2):253. 34966240 PMC8694305

[32] Upadhaya N, Jordans MJD, Pokhrel R, Gurung D, Adhikari RP, Petersen I, et al. Current situations and future directions for mental health system governance in Nepal: findings from a qualitative study. Int J Ment Health Syst. 2017;11(1):37. 10.1186/s13033-017-0145-3. 28603549 10.1186/s13033-017-0145-3 PMC5465682

[33] Dhungana N, Khadka C, Bhatta B, Regmi S. Barriers in local climate change adaption planning in Nepal. JL Pol’y Globalization. 2017;62:20.

[34] Staniszewska S, Brett J, Simera I, Seers K, Mockford C, Goodlad S, et al. GRIPP2 reporting checklists: tools to improve reporting of patient and public involvement in research. bmj. 2017. p. 358. 10.1136/bmj.j3453. 10.1136/bmj.j3453 PMC5539518 28768629

[35] Mishra SR, Joshi B, Poudyal Y, Adhikari B. Epistemic indebtedness: do we owe to epistemic enterprises? J Glob Health Econ Policy. 2022;2:2022012. 10.7189/001c.36869.

[36] Adhikari S, Rijal KR, Parker DM, Ghimire P, Cheah PY, Adhikari B. Stakeholder analysis for ‘one health’ approach to tackle antimicrobial resistance. BMJ Glob Health. 2025;10(10). Epub 20251021. doi: e019236. 10.1136/bmjgh-2025-019236. 10.1136/bmjgh-2025-019236 PMC12548578 41125295

[37] Singh DR, Sunuwar DR, Shah SK, Karki K, Sah LK, Adhikari B, et al. Impact of COVID-19 on health services utilization in Province-2 of Nepal: a qualitative study among community members and stakeholders. BMC Health Serv Res. 2021;21(1):174. Epub 2021/02/26. 10.1186/s12913-021-06176-y. 33627115 10.1186/s12913-021-06176-y PMC7903406

[38] Singh DR, Sunuwar DR, Adhikari B, Szabo S, Padmadas SS. The perils of COVID-19 in Nepal: implications for population health and nutritional status. J Global Health. 2020;10(1):010378. Epub 2020/06/26. doi: 10.7189/jogh.10.010378. 10.7189/jogh.10.010378 PMC7307805 32582440

[39] Saunders B, Sim J, Kingstone T, Baker S, Waterfield J, Bartlam B, et al. Saturation in qualitative research: exploring its conceptualization and operationalization. Qual Quant. 2018;52(4):1893–907. Epub 2018/06/26. 10.1007/s11135-017-0574-8. 29937585 10.1007/s11135-017-0574-8 PMC5993836

[40] Kulnik ST, Salbrechter S, Radwanovsky S, Garstenauer U, Ebner M, Fuhrmeister T, et al. Ganz Salzburg bewegen [All of Salzburg moving]: a large-scale public involvement project with underserved groups, for the co-design of local and contextualized physical activity promotion concepts. Research Involv Engagem. 2025;11(1):92. 10.1186/s40900-025-00766-4. 10.1186/s40900-025-00766-4 PMC12323020 40764598

[41] Kickbusch I, Szabo MMC. A new governance space for health. Globalizat Health Action. 2014;7(1):23507. 10.3402/gha.v7.23507. 10.3402/gha.v7.23507 PMC3925805 24560259

[42] Adhikari B, Amaratunga C, Mukumbang FC, Mishra SR. Why should we be concerned by internalised racism in global health? BMJ Glob Health. 2025;10(6). Epub 20250616. e016740. 10.1136/bmjgh-2024-016740. 10.1136/bmjgh-2024-016740 PMC12182036 40527526

[43] Adhikari B, Mishra SR. Engaging pose and gaze in global health research. The Lancet Infect Dis. 2025;25(2):154. Epub 20250110. 10.1016/S1473-3099(25)00012-X. 39805307 10.1016/S1473-3099(25)00012-X

[44] Kaisler RE, Missbach B. Co-creating a patient and public involvement and engagement ‘how to’guide for researchers. Research Involv Engagem. 2020;6(1):32. 10.1186/s40900-020-00208-3. 10.1186/s40900-020-00208-3 PMC7301967 32566249

[45] Acemoglu D, Robinson JA. Why nations fail: the origins of power, prosperity, and poverty. 9th August 2025. Available online at https://bit.ly/4li9cMQ.

[46] Vincent R, Adhikari B, Duddy C, Richardson E, Wong G, Lavery J, et al. ‘Working relationships’ across difference-a realist review of community engagement with malaria research. Wellcome open research. 2022, 7. 10.12688/wellcomeopenres.17192.1 PMC10444998 37621950

[47] Adhikari B, Vincent R, Wong G, Duddy C, Richardson E, Lavery JV, et al. A realist review of community engagement with health research. Wellcome Open Res. 2019;4:87. Epub 2019/08/27. 10.12688/wellcomeopenres.15298.1. 31289754 10.12688/wellcomeopenres.15298.1 PMC6611131

[48] Kronfol N. Access and barriers to health care delivery in Arab countries: a review. Easter Mediterr Health J. 2012;18(12):1239–46. 10.26719/2012.18.12.1239. 10.26719/2012.18.12.1239 23301399

[49] UNAIDS. Community-led aids responses final report based on the recommendations of the multistakeholder task team. 31 August 2025. 2022. Available online at https://bit.ly/46e5hw6.

[50] Nashwan AJ, Shaban MM, Kamugisha JB. Bridging the gap: how investing in advanced practice nurses could transform emergency care in Africa. Int Nurs Rev. 2024;71(2):285–90. 10.1111/inr.12966. 38613148 10.1111/inr.12966

[51] Adhikari B, Bayo M, Peto TJ, Callery JJ, Tripura R, Dysoley L, et al. Comparing the roles of community health workers for malaria control and elimination in Cambodia and Tanzania. BMJ Glob Health. 2023;8(12). Epub 20231209. e013593. 10.1136/bmjgh-2023-013593. 10.1136/bmjgh-2023-013593 PMC10729139 38070880

[52] de Brún T, O’Reilly - de Brún M, van Weel-Baumgarten E, Burns N, Dowrick C, Lionis C, et al. Using participatory learning & action (PLA) research techniques for inter-stakeholder dialogue in primary healthcare: an analysis of stakeholders’ experiences. Research Involv Engagem. 2017;3(1):28. 10.1186/s40900-017-0077-8. 10.1186/s40900-017-0077-8 PMC5718138 29225922

[53] Han H-R, Xu A, Mendez KJW, Okoye S, Cudjoe J, Bahouth M, et al. Exploring community engaged research experiences and preferences: a multi-level qualitative investigation. Research Involv Engagem. 2021;7(1):19. 10.1186/s40900-021-00261-6. 10.1186/s40900-021-00261-6 PMC8008581 33785074

[54] Boateng MA, Agyei-Baffour E, Angel S, Asare O, Prempeh B, Enemark U. Co-creation and prototyping of an intervention focusing on health literacy in management of malaria at community-level in Ghana. Research Involv Engagem. 2021;7(1):55. 10.1186/s40900-021-00302-0. 10.1186/s40900-021-00302-0 PMC8340491 34353378

[55] Roche P, Shimmin C, Hickes S, Khan M, Sherzoi O, Wicklund E, et al. Valuing all voices: refining a trauma-informed, intersectional and critical reflexive framework for patient engagement in health research using a qualitative descriptive approach. Research Involv Engagem. 2020;6(1):42. 10.1186/s40900-020-00217-2. 10.1186/s40900-020-00217-2 PMC7370500 32699647

[56] Acemoglu D, Robinson JA. Why nations fail: the origins of power, prosperity and poverty: profile London; 2012. ASEAN Econ Bull. 2012;29(2):168. 10.1355/ae29-2j.

[57] Smits D-W, Van Meeteren K, Klem M, Alsem M, Ketelaar M. Designing a tool to support patient and public involvement in research projects: the involvement matrix. Research Involv Engagem. 2020;6(1):30. 10.1186/s40900-020-00188-4. 10.1186/s40900-020-00188-4 PMC7296703 32550002

