# Supplementary material for: Community and stakeholder engagement in national priority setting and participatory research for HIV, Tuberculosis, and Malaria programs in Nepal
Source: Res Involv Engagem. 2026 May 22;12:69. doi: 10.1186/s40900-026-00907-3 (PMC13198034; doi:10.1186/s40900-026-00907-3)
Supplement: Supplementary file 2 — Supplementary material 2 [file 40900_2026_907_MOESM2_ESM.docx]

# **Guidelines for the Country Dialogue Process for Developing and National Strategic Plan (NSP) for HIV (2021-2026) and for Priority Setting for Global Fund Grant Cycle 6 and USAID/PEPFAR HIV Funding in Nepal**

# **Background**

Country dialogue is an open and inclusive conversation with people responding to and affected by HIV, TB and Malaria. While the Country Coordinating Mechanism (CCM) itself includes representatives of a wide variety of stakeholders, the purpose of the country dialogue is to go beyond its membership and reach out to all those involved in the prevention, care and treatment of diseases, including those key populations (KPs) affected by the diseases at the grassroots level.

**The purpose of the country dialogue is to serve as a national platform for developing and revising the National Strategic Plan (NSP) for HIV program as well as for identifying and agreeing upon priority areas for resource mobilization under the Global Fund Grant Cycle 6 (GC6) and USAID/PEPFAR HIV funding.** This participatory process ensures that priorities are developed based on reliable data, community inputs, and strategic, harmonized, and consensual guidance of the national stakeholders in connection with the three diseases.

**Country dialogue is an ongoing process beginning before the development of new national strategic plans and continuing through the funding request and implementation phases. The Global Fund encourages countries to develop funding applications that are aligned with national strategies, avoiding fragmentation and ensuring country ownership.**

**The CCM will develop the funding request and strategic priorities based on the National Strategic Plans. The most effective funding requests are those developed with the input of people responding to and affected by the diseases. In line with recent CCM Nepal meetings convened to guide the development of the forthcoming funding cycle, the CCM has made a provision for a “Task Team” (comprising HIV stakeholders) to lead the process. This “Task Team” consists of members from different sectors to ensure inclusivity and meaningful representation from the people who are affected by the diseases or at risk.**

**This process now provides an opportunity for all relevant stakeholders at national, provincial, and local levels to contribute or provide feedback in the development and revision of the National Strategic Plans and priority-setting for resource mobilization under the Global Fund and USAID/PEPFAR mechanisms. The “Task Team” will also develop Terms of Reference (ToR) for national and international consultants, a comprehensive plan for conducting dialogues, and a resource and budget plan. This guideline provides all the details of the country dialogue process at national, subnational, and local levels and outlines all the activities with indicative timelines and responsibilities.**

# **Community Consultation Plan**

**To ensure that the voices of communities and local governments become the core of the NSP revision and funding prioritization process, a community consultation plan will be scheduled. CCM Nepal, in discussion with the HIV, TB and Malaria Task Teams, will roll out community and local-level consultations using participatory methodologies.**

**Community and local-level consultations will ensure greater and meaningful representation from the respective communities; people infected and affected by the diseases, civil society organizations (CSOs), local governments, and other relevant stakeholders. This process will be supported by the HIV, TB and Malaria Task Teams as per need and context. It will also create a platform to share national strategies with key populations, local governments, and communities, and to receive feedback to guide funding and implementation priorities.**

# **Consultation within the key infected and affected groups/communities:**

**The HIV Task Team will jointly develop a consultation plan in discussion and involvement with CCM members representing relevant constituencies and community networks.**

**Community consultations will be organized by the networks themselves with technical assistance from the HIV, TB and Malaria Task Teams. Consultations will be conducted among the following networks and groups to gather voices from national, subnational, and local levels:**

1. **People living with HIV (men and women),**
2. **Migrants,**
3. **TB survivors and affected families,**
4. **PWID (men and women),**
5. **Female Sex Workers,**
6. **TG/MSM/MSW,**
7. **Populations in prisons/closed settings,**
8. **CSOs/NGOs, and**
9. **Right to Health and Women’s Groups.**

**In addition, consultations will be expanded to include local governments (municipalities/rural municipalities) and local health institutions in selected districts, particularly those with higher disease burden, to ensure the integration of local perspectives in national priority-setting.**

**The HIV, Task Team will develop the community consultation plan with a detailed agenda in consultation with respective networks and stakeholders within the agreed timeframe for the forthcoming funding cycle. Similarly, UNAIDS, WHO, USAID/PEPFAR and other technical partners will provide facilitation and coordination support.**

**Representation from the respective communities, networks, and beneficiaries from the key populations and people affected by the diseases must be ensured, with meaningful participation from women, youth, and vulnerable groups.**

**Recommendations of the community and local-level consultations will be taken to the provincial consultations, country dialogue, and/or directly incorporated into the NSP revision and funding application process as appropriate. Reports of all consultations will be shared with the CCM Nepal Secretariat within the designated timeframe, summarizing major recommendations, outcomes, and challenges.**

***(Suggested areas for the community consultations are attached in Annex A.)
(Evidence/Requirements: Invitations, meeting notes, attendance sheets, photographs, or any digital evidence.)***

# **Stakeholders consultation Plan**

**To make the country dialogue process more inclusive HIV Task Team will facilitate and conduct various activities to ensure the stakeholders engagement throughout the strategic planning and funding prioritization process.**

**Analysis on Funding Gap:**

**With the help of key partners and national programs (NCASC) a preliminary funding and programmatic gap analysis will be conducted to inform NSP updates and the next funding cycle. This will be done based on current information and government allocations to implement national strategies.**

**Donor Meeting:**

**Acknowledging the multi-sectoral nature of disease responses, the National Programs and CCM Nepal will organize a donor coordination meeting to share the current investment situation and discuss co-financing opportunities. Current government funding, Global Fund grants, and USAID/PEPFAR support are major contributors to the three programs. This meeting will identify areas where Global Fund and USAID support should be prioritized and aligned.**

**Provincial Consultations with the partners and key stakeholders:**

As part of the country dialogue, the HIV Task Team will conduct **provincial consultations** at seven provincial locations and in selected districts.

All consultations will aim to:

- Generate recommendations to strengthen services within communities, local governments,

and health facilities;

- Prioritize activities and strategies from the NSPs to maximize the impact of Global Fund and USAID investments;
- Align subnational priorities with national NSP frameworks;
- Gather inputs from all levels- **community, local, and provincial** to inform NSP revisions and funding proposals.

The HIV Task Team will work closely with NCASC and other national programs at the central level, and with **Provincial Ministries of Social Development, Provincial Health Directorates, and local health offices** for coordination.

Consultations will also involve CSOs, EDPs, and networks of affected populations. **Respective CCM members will facilitate the inclusiveness and integrity of these consultations.**

Recommendations from the provincial and local consultations will be synthesized and taken to the federal dialogue and relevant forums. **All consultations will be completed within the agreed timeline for the funding cycle**, and reports will be submitted to the CCM Secretariat highlighting key findings, recommendations, and gaps.

**Tentative locations for provincial consultations:**

1. Sudurpaschim Province: Dhangadi
2. Karnali Province: Surkhet
3. Lumbini Province: Butwal
4. Gandaki Province: Pokhara
5. Bagmati Province: Hetauda
6. Koshi Province: Biratnagar
7. Madhesh Province: Janakpur

**(Note: CCM Nepal Secretariat will coordinate invitation letters and communication through the respective national programs and provincial authorities.)**

**Suggested areas for the provincial and local consultations are attached in Annex B.**
**(Evidence/Requirements: Invitations, participant selection documentation, meeting notes, attendance sheets, photos, and conflict of interest management, if applicable.)**

# **Federal Level Country Dialogue**

A Federal-Level Country Dialogue Meeting will be organized with a broad range of participants from federal, provincial, local, and community levels. The purpose is to identify national needs, finalize NSP priorities, strengthen resource mobilization, and ensure synergy across disease programs.

All relevant actors will be engaged, including government, bilateral and multilateral partners, civil society, technical partners, private sector, networks of key populations, communities affected by the diseases, women’s groups, youth-led organizations, and representatives from provincial and local levels.

The meeting will be organized after completion of community, local, and provincial consultations, gap analysis, thematic discussions, and donor meetings.

During the Federal Dialogue, national investment priorities and co-financing commitments will be presented. Based on recommendations from earlier consultations, a consolidated list of priority areas for Global Fund and USAID/PEPFAR investments will be developed.

The Federal Dialogue will also review Global Fund guidance in the following areas:

- Increase coverage of key populations to 90%
- Case finding and case management
- Elimination of vertical transmission (eVT)
- Co-infection management
- Human rights and gender barriers
- Strategic Information (SI)
- Laboratory systems
- Resilient and Sustainable Systems for Health (RSSH)
- Supply chain management

(Suggested areas for discussion are attached in Annex C.)

(Evidence/Requirements: Invitations, participant selection documentation, meeting notes, attendance sheets, photographs, or other digital evidence.)

**Use of Social Media/Hotline/Email**

An official CCM Nepal website, social media platforms, and hotline/email mechanisms will be fully utilized to increase access and engagement among stakeholders. This will help gather feedback from key and general populations at national, provincial, and local levels throughout the NSP revision and funding prioritization process.

Information will be shared via website, social media, and other digital media (TV, radio).
Designated personnel will document recommendations for integration into subsequent consultations, dialogues, and the final writing process.

**NSP/funding application writing process**

The HIV Task Team, with representation from government, CSOs, development partners, and key populations, will work closely with national and international consultants throughout the writing period to ensure quality and consistency.

**Thematic Team Meeting**

The writing team will coordinate with thematic sub-teams (e.g., PSM, Treatment, Prevention, SI, RSSH, Laboratory, Gender, and Human Rights) as required.

**Validation Workshop:**

Before finalization of the NSP and the funding application, a **validation workshop will be organized to review and confirm** that priorities from the community, local, provincial, and federal consultations are adequately reflected. Representatives from all levels will validate and endorse the draft NSP and proposals.

**Endorsement Process**

Following validation, a final endorsement meeting will be held with all stakeholders participating in the country dialogue. The endorsed proposal and NSP revisions will be shared with all CCM members, Principal Recipients, and dialogue participants for transparency.

**Annex A: Topics and procedures for community and local-level consultations**

To capture voices, priorities, and recommendations from communities, key and vulnerable populations, and local governments across diverse epidemiological and geographic contexts to inform the revision of the National Strategic Plan (NSP) for HIV and the preparation of funding requests for Global Fund Grant Cycle 6 and USAID/PEPFAR HIV support.

### **Structure of Community and Local-Level Consultations**

### **Community and Local-Level Consultations** will be conducted in selected districts and municipalities across all provinces. These consultations will combine participatory discussions with structured feedback on service gaps, access barriers, and local priorities.

Each consultation will include:

- **Community-based consultations** led by networks of key and affected populations;
- **Local government consultations** involving municipal health offices, district hospitals, and local CSOs;
- **Joint sessions** where community and local government representatives discuss shared solutions and accountability mechanisms.

### **Consultation Topics**

### **Access and Coverage:**

- Availability of HIV, TB, and malaria services (testing, treatment, prevention, and care).
- Gaps in service reach among key and vulnerable populations.
- Integration of services within primary health care and local health systems.

1. **Barriers to Services:**

- Stigma and discrimination at health facilities.
- Gender, human rights, and social barriers.
- Structural or logistical challenges (distance, cost, and workforce).

1. **Role of Local Governments:**

- Local resource allocation and co-financing commitments.
- Coordination with community-based organizations (CBOs) and health offices.
- Monitoring and reporting through municipal health systems.

1. **Community-led and Key Population Priorities:**

- Strengthening peer-led services and outreach.
- Community monitoring and accountability.
- Recommendations for domestic resource mobilization at municipal level.

1. **Cross-cutting Issues:**

- Digital reporting and data use.
- Multi-disease integration.
- Sustainability of interventions post-external funding.

### **Participant Selection Procedure**

### **Step 1: Identification of Consultation Sites**

### Sites (districts and municipalities) will be selected based on:

### Disease burden (high-prevalence or hard-to-reach areas).

### Representation of both urban and rural settings.

### Presence of active key population networks and CBOs.

### Provincial balance (at least one district from each province).

**Step 2: Selection of Participants**

Each consultation will aim for balanced and inclusive representation:

- Community representatives: PLHIV, TB survivors, malaria-affected families, FSWs, MSM/TG, PWID, migrants, and youth.
- Local governments: Municipal health section officials, mayor/deputy mayor or health focal persons.
- Health facilities: ART center in-charges, DMC focal persons, malaria surveillance officers, and public health inspectors.
- Civil society: Local NGOs/SRs, CBOs, rights-based organizations.
- Development partners (as observers): WHO, UNAIDS, USAID, and provincial health directorates.

**Step 3: Roles and Responsibilities**

- **Local networks of key populations** will coordinate participant mobilization.
- **Municipal health sections** will nominate local government representatives.
- **Task Team focal points** will review participant lists to ensure inclusivity (gender, geography, key population).
- **CCM Secretariat** will approve final lists and issue formal invitations.

**Step 4: Documentation**

- Attendance sheets (with gender, organization, and constituency).
- Participant selection record (showing inclusion of at-risk and marginalized groups).
- Summary consultation reports with photos and key discussion notes.

### **Facilitation and Reporting**

- **Facilitators**: Trained from within local communities or networks; oriented by the Task Teams on objectives, facilitation methods, and documentation standards.
- **Report format**: Each report will summarize major issues, recommendations, and suggested priority interventions under HIV, TB, and malaria.
- **Submission**: Draft NSP/Funding Application will be submitted to the CCM Secretariat/ Entities director within the agreed timeline.

**Annex B: Provincial Consultation Framework and Participant Selection**

To consolidate provincial-level perspectives, validate findings from local and community consultations, and ensure alignment with the NSP priorities and Global Fund/USAID resource frameworks.

### **Structure and Process**

Provincial consultations will be organized in seven provinces, facilitated by the **HIV Task Team** in collaboration with:

- **Provincial Health Directorates**
- **Provincial Ministries of Social Development**
- **District/Health Offices**
- **Local governments and networks**

Each consultation will last **one to two days,** combining:

- Technical review of NSP priorities;
- Group discussions by thematic area
- Reflection of feedback from local and community consultations;
- Drafting of preliminary provincial recommendations.

### **Consultation Topics**

### **Review of Service Coverage and Gaps**

- Service mapping by disease and key population.
- Partner activities and sub-recipient (SR) coverage.
- Human resource and logistical constraints.

1. **Programmatic Priorities and Funding Alignment**

- Interventions with proven impact and cost-effectiveness.
- Sustainability plans and domestic co-financing at the provincial level.
- Priority areas for GF GC6 and USAID/PEPFAR HIV.

1. **Governance and Coordination**

- Integration with provincial health systems and planning cycles.
- Linkages between municipalities, provincial ministries, and national programs.

1. **Cross-Cutting Themes**

- Gender, human rights, and key population empowerment.
- Laboratory systems, surveillance, and data strengthening.
- Multi-disease coordination and digital health tools.

### **Participant Selection Procedure**

### **Step 1: Coordination and Invitation**

### The **CCM Secretariat,** in coordination with provincial authorities, will issue official invitations.

### The **Task Team** will identify the focal points responsible for mobilization in each province.

**Step 2: Composition of Participants**

Each consultation will target approximately **40–60 participants**, including:

- Provincial Health Directorate: Director and program chiefs for TB, HIV, and malaria.
- Provincial Ministry of Social Development: Health division chiefs and officers.
- District/Health Offices: Chief and focal points from high-burden districts.
- Municipal Governments: Representatives from health sections of selected municipalities.
- Civil Society and Networks: Provincial focal persons of PLHIV, TB survivors, malaria volunteers, KP networks.
- Health Facilities: ART, DOTS, and malaria treatment center focal persons.
- Development Partners: Representatives of WHO, UNAIDS, USAID, Save the Children (PR), and key SRs.

**Step 3: Inclusion Criteria**

- Each province must ensure gender balance (minimum 40% female participants).
- At least 25% of participants should represent communities or civil society.
- All key populations present in the province must have representation.

**Step 4: Documentation**

- Participant lists (signed, verified, with gender and constituency details).
- Photographic documentation.
- Provincial consultation report capturing major recommendations and challenges.

### **Facilitation and Reporting**

- **Facilitation Team:** Provincial-level facilitators from national programs, supported by local experts and community representatives.
- **Outputs:**
  - Consolidated provincial recommendations.
  - Priority interventions by disease and thematic area.
  - Provincial-level investment mapping (Nepal Government, GF, USAID, government).
- **Reporting:** Reports will be compiled within two weeks of each consultation and shared with the CCM Secretariat.

**Annex C: Federal-Level Country Dialogue Framework and Procedures**

This is conducted to synthesize all recommendations from community, local, and provincial consultations into a unified national consensus on strategic priorities and funding allocation for HIV, TB, and malaria. The Federal-Level Country Dialogue Meeting will be organized in Kathmandu, facilitated by the CCM Secretariat and the HIV Task Team. The meeting will last two days and will be attended by high-level representatives from federal, provincial, and local governments, development partners, CSOs, and networks of affected populations.

### **Agenda and Discussion Themes**

1. **Presentation of Consultation Findings**
   - Key recommendations from local and provincial dialogues.
   - Funding and implementation gaps identified across the three programs.
2. **Strategic Prioritization**
   - Review of NSP targets and alignment with GF GC6 and USAID/PEPFAR.
   - Endorsement of multi-disease and cross-cutting priorities.
3. **Investment and Resource Mobilization**
   - Government commitments and co-financing.
   - Partner alignment and sustainability planning.
4. **Cross-Cutting Areas**
   - Gender, human rights, and community systems strengthening.
   - Digital health innovations and data systems.
   - Supply chain and RSSH integration.

### **Participant Selection Procedure**

**Step 1: Nomination and Endorsement**

- National program will nominate delegates.
- Provincial health directorates will nominate two representatives per province.
- Key population networks and CSOs will nominate community delegates through their national secretariats.
- Development partners (UNAIDS, WHO, USAID, GF PRs/SRs) will nominate technical and financial representatives.
- CCM members and secretariat staff will be included ex officio.

**Step 2: Verification**

The **CCM Secretariat** will review all nominations to ensure:

- Representation from all seven provinces;
- Gender and constituency balance;
- Inclusion of both program and community perspectives.

**Step 3: Confirmation and Communication**

Formal invitation letters will be issued by the **CCM Secretariat** with agenda and logistical details.

### **Facilitation and Documentation**

- **Facilitation Team:** Led by national consultants with participation from CCM members, PRs, and technical partners.
- **Outputs:**
  - Consensus statement on national strategic priorities.
  - Agreed list of investment priorities for GF GC6 and USAID/PEPFAR.
  - Final validation for integration into NSPs.
- **Documentation:**
  - Attendance lists, photographs, and meeting notes.
  - Synthesis report with recommendations submitted to CCM for endorsement.
