# Supplementary material for: Community and stakeholder engagement in national priority setting and participatory research for HIV, Tuberculosis, and Malaria programs in Nepal
Source: Res Involv Engagem. 2026 May 22;12:69. doi: 10.1186/s40900-026-00907-3 (PMC13198034; doi:10.1186/s40900-026-00907-3)
Supplement: Supplementary file 3 — Supplementary material 3 [file 40900_2026_907_MOESM3_ESM.docx]

# **Guidelines for the Country Dialogue Process for Developing and Revising the National Strategic Plan (NSP) for Tuberculosis (2021–2026) and for Priority-Setting for Global Fund GC6 in Nepal**

# **Background**

Country dialogue is an open, inclusive, and continuous process that centers the voices of people responding to and affected by TB. While the Country Coordinating Mechanism (CCM) includes diverse stakeholders, the country dialogue deliberately goes beyond CCM membership to engage communities, key and vulnerable populations, implementers, and sub-national authorities.

The dialogue serves two purposes:

1. a national platform to develop or **revise the TB NSP (2021–2026),** and
2. a participatory mechanism to **identify and agree TB investment priorities** for **Global Fund Grant Cycle 6 (GC6).**

Priorities must be grounded in epidemiology, routine data, operational evidence, and lived experience harmonized across partners and owned by country stakeholders.

The CCM will steward funding requests aligned with the NSP. To ensure inclusivity and technical rigor, **CCM Nepal** convenes a **TB Task Team** (under the CCM Task Team arrangement) with representation from government (MoHP/DoHS/NTP/NTC), civil society, key populations, PLHIV, survivors, professional bodies, development partners, and private sector. The TB Task Team may form thematic sub-groups, draft ToRs for national/international consultants, and resource a comprehensive consultation and writing plan.

# Community Consultation Plan

To center community and local government voices in NSP revision and funding prioritization, CCM Nepal through the TB Task Team will roll out structured consultations at community and local levels using participatory methods (FGDs, key-informant interviews, community scorecards, rapid barrier/solution mapping).

**Objectives**

- Surface service gaps, access barriers, stigma/human-rights issues, and gender/age disparities.
- Identify high-impact, locally feasible interventions, including **community-led** actions.
- Build consensus with municipalities/rural municipalities on co-financing and accountability.

**Who to consult (illustrative)**

- TB patients (men/women), DR-TB patients, TB survivors & affected families
- PLHIV and other co-morbid groups (e.g., diabetes, under-nutrition, immunosuppression)
- Migrants (internal/cross-border), prison/closed settings, urban poor
- Frontline TB health workers & lab staff; FCHVs; CBDOT providers
- Private sector (pharmacies, private hospitals/clinics, informal providers)
- CSOs/Networks, youth and women’s groups, human-rights actors, media

**Process**

- TB Task Team develops a **Community Consultation Plan** and agenda with networks and sub-national counterparts; identifies high-burden/underserved sites for inclusion.
- Networks lead their own consultations with technical support from TB Task Team; municipal health sections join joint community–LG sessions to agree follow-ups.
- **Outputs:** brief reports (major issues, recommended priorities, suggested indicators) submitted to CCM Secretariat within the agreed window for synthesis upstream.

**Evidence/Requirements**

Invitations, participant selection records (gender/age/constituency), meeting notes, attendance sheets, photos/digital evidence, and any COI management notes.

# Stakeholder Consultation Plan

To ensure whole-of-system inputs:

### **Funding & Programmatic Gap Analysis**

### **NTP/NTC,** with partners, compiles a rapid gap analysis against NSP targets, costed plans, and GoN allocations; highlights under-funded interventions (e.g., DR-TB care, paediatric TB, PPM, digital SI, specimen transport) to guide priority-setting.

### **Donor/Partner Coordination**

### **CCM/NTP convene a coordination meeting with GoN, Global Fund PR/SRs, USAID, WHO and other EDPs to review current and projected investments, co-financing, and alignment opportunities; agree principles to avoid duplication and optimize catalytic levers (PPM, CSS/RSSH, SI).**

# **Provincial & Local Consultations**

The **TB Task Team,** with NTP/NTC, Provincial Health Directorates/MDs, and local health offices, will conduct provincial dialogues (target: all 7 provinces) and selected district/municipal consultations.

**Aims**

- Prioritize high-impact TB interventions in facilities, communities, and local governments.
- Align sub-national recommendations with NSP pillars and GF/USAID focus areas.
- Capture operational considerations: HR, labs, supply chain, digital reporting, PPM, migrant pathways.

**Tentative provincial locations**

Sudurpashchim (Dhangadhi); Karnali (Surkhet); Lumbini (Butwal); Gandaki (Pokhara); Bagmati

(Hetauda/Ktm Valley as needed); Koshi (Biratnagar); Madhesh (Janakpur).

**Outputs**: short provincial memos (priorities, enablers/barriers, co-financing prospects, key indicators) to CCM Secretariat.

**Evidence/Requirements**

Invitations, participant selection documentation, notes, attendance, photos; COI management where relevant.

## **Federal-Level Country Dialogue**

A 2-day federal dialogue in Kathmandu (post community/local/provincial rounds) synthesizes recommendations and agrees key strategies for NSP 2021-2026 and a **consolidated TB investment package** for GF GC6.

**Core agenda**

- **TB case finding & notification** (including paediatric TB; ACF/contact investigation; migrant strategies)
- **DR-TB** (universal DST; shorter, all-oral regimens; aDSM)
- **TB/HIV** collaboration and co-infection care
- **Private sector engagement (PPM)**- notification, referral, treatment support, digital tools
- **Human rights, gender, and stigma** reduction; **community systems strengthening (CSS)**
- **Strategic Information (SI)/HMIS, laboratory systems** (GeneXpert network, EQA, sample transport)
- **Supply chain** and **RSSH** elements that enable TB outcomes

**Outputs**: consensus list of TB priorities, co-financing map, and endorsement for integration into NSP and funding requests.

## **Communications & Feedback Channels**

CCM website and social media + hotline/email to widen access for feedback nationwide. Designated staff capture and route inputs into provincial/federal dialogues and the writing team’s tracker.

## **Writing Process & Thematic Teams**

A **Writing Team** (Govt/NTP/NTC, PR/SRs, CSOs, key populations, EDPs; consultants) coordinates with **thematic sub-teams** (Case-finding & Paediatrics; DR-TB; TB/HIV & comorbidities; PPM/PPM-digital; Infection Prevention & TB Infection; Treatment & aDSM; SI/M&E; Labs; Supply Chain; CSS/Human Rights/Gender; RSSH). The writing calendar aligns to GF/USAID windows; drafts are iterated with Task Team oversight.

## **Validation & Endorsement**

A national **Validation Workshop** confirms that community, local, provincial, and federal inputs are reflected. After final edits, CCM convenes an **Endorsement Meeting** with all dialogue participants. Final NSP revisions and funding requests are shared with CCM members, PRs, and dialogue participants for transparency.

**Annex A: Plan for consultation meeting**

| **SN** | **Activities** | **Province** | | | | | | |  |
| --- | --- | --- | --- | --- | --- | --- | --- | --- | --- |
|  |  | **Koshi** | **Madesh** | **Bagmati** | **Gandaki** | **Lumbini** | **Karnali** | **Sudurpaschim** | **Total** |
| 1 | Meeting with Provincial Authorities | Y | Y |  | Y | Y | Y | Y | 6 |
| 2 | Meeting with DRTB Patients | Y |  |  |  | Y |  |  | 2 |
| 3 | Meeting with Private Health Care Provider | Y |  |  |  |  |  |  | 1 |
| 4 | Meeting with Vulnerable group-Prison/PHLIV | Y |  |  |  |  |  | Y | 2 |
| 5 | Meeting with Health care providers (Public) | Y | Y |  |  |  |  |  | 2 |
| 6 | Meeting with TB patients | Y |  |  |  | Y |  |  | 2 |
| 7 | Meeting with Municipal Offices |  | Y | Y |  | Y |  |  | 3 |
| 8 | FGD with Marginalized Community (Musar or Other ethnic groups) | Y | Y |  |  |  | Y |  | 3 |
| 9 | FGD with Factory Workers |  | Y |  |  |  |  |  | 1 |
| 10 | Meeting with Pharmacy |  | Y |  |  |  |  |  | 1 |
| 11 | Meeting with Elderly People at Devghat |  |  |  | Y |  |  |  | 1 |
| 12 | FGD with Refugee people at Pokhara |  |  |  | Y |  |  |  | 1 |
| 13 | Meeting with CBDOT Providers |  |  |  | Y |  |  |  | 1 |
| 14 | Meeting with HFOMC |  |  |  | Y |  |  |  | 1 |
| 15 | Meeting with FCHV, treatment supporters and TB patients |  |  |  | Y |  | Y (3) | Y (2) | 6 |
| 16 | Meeting with TB Survivors |  |  |  |  |  |  |  | 0 |
| 17 | FGD with PHD and Chief of health division of province |  |  | Y |  |  |  |  | 1 |
| 18 | Meeting with different Ministry |  |  | Y |  |  |  |  | 1 |
| 19 | Meeting with NMA, NEPAS, APHIN, MELAN , NCDA, DDA, TSON, DEAN, NPHA, SOPHIN and private hosiptal and association |  |  | Y |  |  |  |  | 1 |
| 20 | Meetign with NGO, SR, INGO, TB network |  |  | Y |  |  |  |  | 1 |
| 21 | Meeting with major hospital, Bir, Kanti, Teaching, Army, Patan, KIST, KMC, Dhulikhel, Norvic, HAMS, Sumeru, Medicity, Civil, Police Hospital, APF |  |  | Y |  |  |  |  | 1 |
| 22 | Meeting with Youth group |  |  | Y |  |  |  |  | 1 |
| 23 | Meeting with District Health officials and partners/municipality |  |  |  |  | Y | Y | Y | 3 |
| 24 | Meeting with TB SR NAP+N on cross boarder issues and seasonal migrant program |  |  |  |  |  |  | Y | 1 |
|  | **Total Events** |  |  |  |  |  |  |  | **43** |

**Topic for Community Consultation**

Community consultations will be led by facilitator from the TB Task team who is oriented earlier in the GF proposal development process.

- Brief sharing – current strategy to scale-up program coverage in KP and access to treatment, care and support; specific activities identified for communities in the implementation plan
- Brief sharing – GF proposal development process and role of communities, objective of the consultation
- Expectations from the communities
  - What are the current TB services that you know is available in your place and other places closer to you?
  - What should be done for you so that you can get easy access to information about TB prevention, diagnosis services and treatment?
  - Where (institutions) do you want these services for you?
  - What support services do you want besides TB prevention, treatment, care and support services?
  - If you want to prioritize the most important ones form the things you shared, what would be those things?
  - What are the barriers in accessing services?

**Community consultation – Selection of participants**

Task team will call a meeting with all networks and share the guidelines with key questions for the community consultation. Networks will select the facilitators for the community consultation meetings. Thus, selected facilitators will be oriented by task team and sub task team on the contents and expected outcomes from the meeting. Network will identify two place to conduct community consultation. The facilitator of the consultation meeting will identify the local representatives for the network and call for a meeting. Facilitators facilitates and meeting and with the help of a note taker will prepare a report of the consultation meeting.

**Annex B: Federal Level Consultation-**

Task team for the GF proposal shall lead and facilitate the country level dialogue/Federal Level Consultation in coordination with TB Task Team.

- Sharing of compiled report/findings/recommendations from community consultation/Provincial consultation
- Prioritization from IP -treatment vs prevention, GoN contribution/commitment in treatment; recommendations from the community and Provincial consultation
- Selection of KPs to be covered by the global fund grant and proposed service modality to increase coverage in KP

**Selection of the participants for Federal Level Country Dialogue:**

CCM Nepal Members representing from their respective constituencies will coordinate with their networks to select the participants through inclusive and democratic process. For government participants CCM Nepal Chair, Health Coordination Division Chief and NTCC will provide nominations for the participants. CCM Nepal member representing from EDP constituencies will select the participants from EDP’s and International NGO in consultation with NTC. WHO, PR, IOM, SR and other relevant stakeholders will be invited as suggested by TB Task Team.

**Annex C: Thematic Group Consultations**

|  |  |  |  |
| --- | --- | --- | --- |
| **Thematic Group Topic (GF)** | **Objective in NSP** | **Strategic Interventions** | **Activities/Sub-activities** |
|  |  |  |  |
| TB:  Case detection & diagnosis | **Objective 1:** **Achieve an incremental increase of 12% per year from 2021 up to 60% in 2025 in TB case notification compared with the level in 2020 by systematic screening of contacts and high-risk groups** | SI 1- Strengthening and expansion of health facility-based TB case finding by using sensitive screening and diagnostic tools  SI 2- Strengthening and expansion of active case finding of TB in communities  SI 3- Strengthening and expansion of contact investigation  SI 4- Scaling up systematic screening among high risk and vulnerable groups  SI 7- Strengthening TB referral mechanism by innovative approaches to prevent initial lost to follow-up of TB cases  SI8- Strengthening diagnosis and management of childhood TB |  |

|  |  |  |  |
| --- | --- | --- | --- |
| **Thematic Group Topic (GF)** | **Objective in NSP** | **Strategic Interventions** | **Activities/Sub-activities** |
|  |  |  |  |
| TB:  Case detection & diagnosis | **Objective 2:** **To achieve an incremental increase of 18% per year from 2021 up to 90% in 2025 in DR-TB case notification compared with the level in 2020 by universal DST** | SI2 Ensure all DR TB case detected are enrolled on treatment |  |

|  |  |  |  |
| --- | --- | --- | --- |
| **Thematic Group Topic (GF)** | **Objective in NSP** | **Strategic Interventions** | **Activities/Sub-activities** |
|  |  |  |  |
| **PPM** | **Objective 4: To develop and scale-up a comprehensive approach for engagement of private sector in TB care and prevention** | SI2 Advocate for political commitment, action, and investment in PPM and partner with intermediaries and key stakeholders  SI3 Establish a supportive policy and regulatory framework  SI4 Harness the power of digital technologies for reporting and build accountability |  |

|  |  |  |  |
| --- | --- | --- | --- |
| **Thematic Group Topic (GF)** | **Objective in NSP** | **Strategic Interventions** | **Activities/Sub-activities** |
|  |  |  |  |
| **Infection Control** | **Objective 6:: To enroll 80% of eligible childhood contact and 50% of eligible vulnerable population for treatment of TB infection and strengthen implementation of infection control measures** | SI1 Increase coverage of preventive therapy throughout country and among all children contact and vulnerable groups  SI2 Introduction and implementation of new preventive measures  SI3 Strengthen and implement robust infection control measures |  |

|  |  |  |  |
| --- | --- | --- | --- |
| **Thematic Group Topic (GF)** | **Objective in NSP** | **Strategic Interventions** | **Activities/Sub-activities** |
|  |  |  |  |
| **TB and co-morbid** | **Objective 7: Address TB among PLHIV, diabetic, malnourished, smoker and other immuno-compromised through collaborative framework and multisectoral approach** | SI1 Scale up TB/HIV collaborative approaches  SI 2 Reduce the burden of TB in people living with HIV and initiate early antiretroviral therapy  SI 3 Reduce the burden of HIV in patients with presumptive and diagnosed TB  SI 4 Ensure tobacco cessation as an integral part of TB service delivery  SI 6 Detect and manage TB in patients with diabetes  SI 7 Detect and manage diabetes in patients with TB  SI 8 Address TB-under nutrition issues |  |

|  |  |  |  |
| --- | --- | --- | --- |
| **Thematic Group Topic (GF)** | **Objective in NSP** | **Strategic Interventions** | **Activities/Sub-activities** |
|  |  |  |  |
| **HSS** | **Objective 8: To strengthen the health system and improve quality TB services under universal health coverage and ensure no affected family faces catastrophic costs due to TB by 2025** | SI 1 Define quality for TB service and scale up TB service delivery as a part of a basic health care service package within UHC  SI 2 Advocate for political commitment with an increased and sustained resource for TB services  SI 3 Advocate for health system strengthening including adequate human resource (number and skills) in line with the federal context  SI 4 Strengthen capacity of health workers of NTP  SI 5 Re-define partnership at all levels  SI 6 Ensure no affected family faces catastrophic costs due to TB  SI 7 Develop an appropriate strategy for TB response in a disaster setting, ensure implementation in coordination with HEDMO and allocate adequate resources. |  |

|  |  |  |  |
| --- | --- | --- | --- |
| **Thematic Group Topic (GF)** | **Objective in NSP** | **Strategic Interventions** | **Activities/Sub-activities** |
|  |  |  |  |
| **CSS** | **Objective 9: To strengthen and scale up CSS, Civil Society Network engagement and ACSM in TB care and prevention** | SI 1 To create enabling environments and advocacy for TB care and prevention (create vibrant community networks, linkages, partnerships, and coordination)  SI 2 To ensure adequate resources and capacity building for CSS and Civil Society Network for leadership, community activities, and service delivery  SI 3 Planning, monitoring & evaluation  SI 4 Develop and strengthen context specific ACSM activity. |  |

|  |  |  |  |
| --- | --- | --- | --- |
| **Thematic Group Topic (GF)** | **Objective in NSP** | **Strategic Interventions** | **Activities/Sub-activities** |
|  |  |  |  |
| **M & E** | **Objective 10: Scale-up integrated monitoring, evaluation and surveillance system using appropriate digital platform and implement regulatory mechanism** | SI1 Strengthen program review and quarterly cohort analysis at all levels.  SI 2 Ensure quality of TB data in collaboration with IHMIS.  SI 5 Strengthen supportive supervision, monitoring and evaluation at all levels  SI 6 Ensure sufficient resources (human and finance) for supervision, monitoring and evaluation |  |

# **FDG For FCHVs and Mother’s Group.**

## **Objective 1: To achieve an incremental increase of 12% per year from 2021 up to 60% in 2025 in TB case notification compared with the level in 2020 by systematic screening of contacts and high-risk groups**

The objectives is to look to:

Strengthening and expansion of active case finding of TB in communities

- Strengthening and expansion of contact investigation
- Scaling up systematic screening among high risk and vulnerable groups
- To strengthen cross-country collaboration on TB in migrants to ensure proper referral and treatment
- Ensuring effective engagement of community for identification and referral of presumptive TB (To be addressed in CSS section)
- Strengthening TB notification by the private sector using an effective PPM model
- Strengthening TB referral mechanism by innovative approaches to prevent initial lost to follow-up of TB cases
- Strengthening diagnosis and management of childhood TB

### **How can FCHVs, Mother’s Group support in expanding ACF services in communities, improve contact investigations in children and address TB in vulnerable groups.**

## **Objective 2: To achieve an incremental increase of 18% per year from 2021 up-to 90% in 2025 in DR-TB case notification compared with the level in 2020 by universal DST**

SIs Re:

- Ensure universal DST for all TB cases
- Ensure all DR TB case detected are enrolled on treatment

### How can FCHVs and Mother’s Group be engaged to get all those TB patients tested for their DST (eg. Genexpert)

### How can FCHVs and Mother’s Group be engaged to get make sure that those diagnosed with TB are enrolled in Treatment and adhere to the treatment till full course.

## **Objective 3: To strengthen and maintain an effective TB laboratory system**

Finalize and implement the National Lab Network plan

- Strengthening the current NTC mycobacterium laboratory and enable it to play the role of National TB Reference Lab (NTRL).
- Completion of the National Culture/LPA lab network of four laboratories under the National TB Reference Lab (NTRL).
- Establishment of a well-organized GeneXpert lab network and integration with the sample collection centers, microscopy centers and the culture/LPA labs.
- Re-organization of a properly quality assured smear microscopy network
- Involvement of Provincial public health lab for quality assurance of GeneXpert labs and microscopy centers.
- Establishment of a reliable, versatile, sample transportation system.
- Establishment of an effective and fast procurement and logistics management system for the lab network.
- Establishment of an Evaluation and updating system for the lab network.
- Establishment of a system for maintenance of equipment of the lab network.

### How can FCHVs and Mother’s Group engage in strengthening courier and referral system of sputum sample from community to MCs.

## **Objective 4: To develop and scale-up a comprehensive approach for engagement of private sector in TB care and prevention**

- Build understanding about patient-provider pathways, private sector dynamics and design appropriate service package with adequate funding
- Advocate for political commitment, action, and investment in PPM and partner with intermediaries and key stakeholders
- Establish a supportive policy and regulatory framework
- Harness the power of digital technologies for reporting and build accountability

### How can FCHVs and Mother’s Group engage to strengthen private sector (eg. Pharmacies, Traditional healers etc.) practice of good practices of TB program and to strengthen their coordination with NTP/ GoN System.

## **Objective 5: To maintain treatment success above 90% throughout 2025 in drug-susceptible TB and increase treatment success up to 85% by 2025 in rifampicin-resistant TB**

- Strengthen and scale up community-based DOTs throughout the country
- Scale up shorter regimens for the treatment of MDR/RR-TB
- Develop and strengthen a robust referral mechanism for continuation of treatment and outcome assessment for both DS-TB and DR-TB
- Develop and implement a treatment support scheme to address barrier of treatment adherence of drug-susceptible TB patients
- Increase access to quality DR-TB treatment
- Strengthen the quality of DR-TB services provided at health centers
- Increase access to psychosocial, nutritional and financial support for DR-TB patients and their family
- Strengthen aDSM for efficient identification and effective management of adverse reactions
- Ensure uninterrupted supply of quality assured DR TB drugs and other commodities through strengthened supply chain management

### TB treatment is required for minimum of 6 months or longer, to complete and needs to be taken in daily basis to maintain a good success rate. How can FCHVs and Mother’s Group engage in:

### supporting daily DOT by TB patients in community/home for those under TB treatment?

### Support in providing psychosocial, nutritional and other support.

### In identification and referral of side effects to TB patients in communities.

## **Objective 6: To enroll 80% of eligible childhood contact and 50% of eligible vulnerable population for treatment of TB infection and strengthen implementation of infection control measures**

- Increase coverage of preventive therapy throughout country and among all children contact and vulnerable groups
- Introduction and implementation of new preventive measures
- Strengthen and implement robust infection control measures

### TB found in children means that there is a nearby source of infection in family / close contacts. Similar TB in vulnerable population (e.g. Diabetes, HIV, past TB cases, marginalize and poor, elderly, smokers) need more focus to these populations. Children also need TBPT if contacts of index TB cases. How can FCHVs and Mother’s Group engage to make sure that

### Children of all index cases are screened and managed

### To identify, advocate, screen and manage TB in vulnerable population.

## **Objective 7: Address TB among PLHIV, diabetic, malnourished, smoker and other immunocompromised through a collaborative framework and multi-sectoral approach**

### Addressed in question to objective 6

## **Objective 8: To strengthen the health system and improve quality TB services under universal health coverage and ensure no affected family faces catastrophic costs due to TB by 2025**

- Define quality for TB service and scale up TB service delivery as a part of a basic health care service package within UHC
- Advocate for political commitment with an increased and sustained resource for TB services
- Advocate for health system strengthening including adequate human resource (number and skills) in line with the federal context
- Strengthen capacity of health workers of NTP
- Re-define partnership at all levels
- Ensure no affected family faces catastrophic costs due to TB
- Develop an appropriate strategy for TB response in a disaster setting, ensure implementation in coordination with HEDMO and allocate adequate resources.

### Universal health coverage (UHC) aims to provide promotive, preventive, curative, rehabilitative and palliative health services to all citizens. It is essential to ensure that TB care and prevention is included in the UHC framework in Nepal. This needs strong political Commitment with an increased and sustained resource for TB services at all levels. How can FCHVs and Mother’s group engage and support in this process at community level?

## **Objective 9: To strengthen and scale up CSS, Civil Society Network engagement and ACSM in TB care and prevention**

- To create enabling environments and advocacy for TB care and prevention (create vibrant community networks, linkages, partnerships, and coordination)
- To ensure adequate resources and capacity building for CSS and Civil Society Network for leadership, community activities, and service delivery
- Planning, monitoring & evaluation
- Develop and strengthen context specific ACSM activity
- Strengthen ACSM approaches at National and with neighboring country level

### How can FCHVs and Mother’s Group engage to create enabling environments and advocacy for TB care and prevention (create vibrant community networks, linkages, partnerships, and coordination)

### How can FCHVs and Mother’s Group be engaged in Planning, monitoring & evaluation (from bottom up levels)

### How can FCHVs and Mother’s Group support in developing and engaging in ACSM activity?

## Scale-up integrated surveillance system using appropriate digital platform and implement regulatory mechanism

# How can FCHVs and Mother’s Group engage for supervision of patients that are enrolled on treatment at the community level

# **FDG for CBDOT providers and volunteers.**

## **Objective 1: To achieve an incremental increase of 12% per year from 2021 up to 60% in 2025 in TB case notification compared with the level in 2020 by systematic screening of contacts and high-risk groups**

The objectives are to look to:

Strengthening and expansion of active case finding of TB in communities

- Strengthening and expansion of contact investigation
- Scaling up systematic screening among high risk and vulnerable groups
- To strengthen cross-country collaboration on TB in migrants to ensure proper referral and treatment
- Ensuring effective engagement of community for identification and referral of presumptive TB (To be addressed in CSS section)
- Strengthening TB notification by the private sector using an effective PPM model
- Strengthening TB referral mechanism by innovative approaches to prevent initial lost to follow-up of TB cases
- Strengthening diagnosis and management of childhood TB

### Can CBDOT providing volunteers be mobilized and engaged in ACF in community as well? If so how?

## **Objective 2: To achieve an incremental increase of 18% per year from 2021 up-to 90% in 2025 in DR-TB case notification compared with the level in 2020 by universal DST**

SIs Re:

- Ensure universal DST for all TB cases
- Ensure all DR TB case detected are enrolled on treatment

### How can CBDOT providers’ and volunteers engage in getting all TB patients tested (for DST) who are identified as TB in communities.

## **Objective 3: To strengthen and maintain an effective TB laboratory system**

Finalize and implement the National Lab Network plan

- Strengthening the current NTC mycobacterium laboratory and enable it to play the role of National TB Reference Lab (NTRL).
- Completion of the National Culture/LPA lab network of four laboratories under the National TB Reference Lab (NTRL).
- Establishment of a well-organized GeneXpert lab network and integration with the sample collection centers, microscopy centers and the culture/LPA labs.
- Re-organization of a properly quality assured smear microscopy network
- Involvement of Provincial public health lab for quality assurance of GeneXpert labs and microscopy centers.
- Establishment of a reliable, versatile, sample transportation system.
- Establishment of an effective and fast procurement and logistics management system for the lab network.
- Establishment of an Evaluation and updating system for the lab network.
- Establishment of a system for maintenance of equipment of the lab network.

### How can CBDOT providers and volunteers engage in collection and transportation of sputum from all TB patients if no follow up is made by them to HF.

### How can CBDOT providers and volunteers engage in collection of sputum and transport to MCs if preemptive TB are identified in community.

## **Objective 4: To develop and scale-up a comprehensive approach for engagement of private sector in TB care and prevention**

- Build understanding about patient-provider pathways, private sector dynamics and design appropriate service package with adequate funding
- Advocate for political commitment, action, and investment in PPM and partner with intermediaries and key stakeholders
- Establish a supportive policy and regulatory framework
- Harness the power of digital technologies for reporting and build accountability

### If private sector is managing TB cases, how can CBDOT volunteer engage to support in managing those cases in the community as well?

## **Objective 5: To maintain treatment success above 90% throughout 2025 in drug-susceptible TB and increase treatment success up to 85% by 2025 in rifampicin-resistant TB**

- Strengthen and scale up community-based DOTs throughout the country
- Scale up shorter regimens for the treatment of MDR/RR-TB
- Develop and strengthen a robust referral mechanism for continuation of treatment and outcome assessment for both DS-TB and DR-TB
- Develop and implement a treatment support scheme to address barrier of treatment adherence of drug-susceptible TB patients
- Increase access to quality DR-TB treatment
- Strengthen the quality of DR-TB services provided at health centers
- Increase access to psychosocial, nutritional and financial support for DR-TB patients and their family
- Strengthen aDSM for efficient identification and effective management of adverse reactions
- Ensure uninterrupted supply of quality assured DR TB drugs and other commodities through strengthened supply chain management

### What should be the criteria for a patient to be eligible for CBDOT management.

### What should be the criteria for selection of volunteers and how engage them including incentive management. How they the providers be also engaged for CBDOT for DRTB

### What should be the capacity building process for them to keep them engaged.

### What should be the working mechanism of CBDOT providing volunteers at community.

### What should be the follow up mechanism of CBDOT focal person at Health facility.

## **Objective 6: To enroll 80% of eligible childhood contact and 50% of eligible vulnerable population for treatment of TB infection and strengthen implementation of infection control measures**

- Increase coverage of preventive therapy throughout country and among all children contact and vulnerable groups
- Introduction and implementation of new preventive measures
- Strengthen and implement robust infection control measures

### How can CBDOTs provider be also engaged for management of childhood Contacts/vulnerable population and also in ensuring promotion of infection control measures at household level.

## **Objective 7: Address TB among PLHIV, diabetic, malnourished, smoker and other immunocompromised through a collaborative framework and multi-sectoral approach**

### Addressed in question to objective 6

## **Objective 8: To strengthen the health system and improve quality TB services under universal health coverage and ensure no affected family faces catastrophic costs due to TB by 2025**

- Define quality for TB service and scale up TB service delivery as a part of a basic health care service package within UHC
- Advocate for political commitment with an increased and sustained resource for TB services
- Advocate for health system strengthening including adequate human resource (number and skills) in line with the federal context
- Strengthen capacity of health workers of NTP
- Re-define partnership at all levels
- Ensure no affected family faces catastrophic costs due to TB
- Develop an appropriate strategy for TB response in a disaster setting, ensure implementation in coordination with HEDMO and allocate adequate resources.

### Not Applicable to CBDOT provider.

## **Objective 9: To strengthen and scale up CSS, Civil Society Network engagement and ACSM in TB care and prevention**

- To create enabling environments and advocacy for TB care and prevention (create vibrant community networks, linkages, partnerships, and coordination)
- To ensure adequate resources and capacity building for CSS and Civil Society Network for leadership, community activities, and service delivery
- Planning, monitoring & evaluation
- Develop and strengthen context specific ACSM activity
- Strengthen ACSM approaches at National and with neighboring country level

### Not Applicable for CBDOT

## Scale-up integrated surveillance system using appropriate digital platform and implement regulatory mechanism

### How can CBDOT reporting and recording system be made more robust?

**FDG with Youth Group.**

## **Objective 1: To achieve an incremental increase of 12% per year from 2021 up to 60% in 2025 in TB case notification compared with the level in 2020 by systematic screening of contacts and high-risk groups**

The objectives is to look to:

Strengthening and expansion of active case finding of TB in communities

- Strengthening and expansion of contact investigation
- Scaling up systematic screening among high risk and vulnerable groups
- To strengthen cross-country collaboration on TB in migrants to ensure proper referral and treatment
- Ensuring effective engagement of community for identification and referral of presumptive TB (To be addressed in CSS section)
- Strengthening TB notification by the private sector using an effective PPM model
- Strengthening TB referral mechanism by innovative approaches to prevent initial lost to follow-up of TB cases
- Strengthening diagnosis and management of childhood TB

### How can youth be used to expanding ACF services in communities, improve contact investigations in children and address TB in vulnerable groups.

## **Objective 2: To achieve an incremental increase of 18% per year from 2021 up-to 90% in 2025 in DR-TB case notification compared with the level in 2020 by universal DST**

SIs Re:

- Ensure universal DST for all TB cases
- Ensure all DR TB case detected are enrolled on treatment

### No Direct Role of Youth.

## **Objective 3: To strengthen and maintain an effective TB laboratory system**

### No Direct Role of Youth

## **Objective 4: To develop and scale-up a comprehensive approach for engagement of private sector in TB care and prevention**

### How can youth engage in improving quality TB services in private sectors?

### How can youth engage in improving better coordination between private sector and NTP system?

## **Objective 5: To maintain treatment success above 90% throughout 2025 in drug-susceptible TB and increase treatment success up to 85% by 2025 in rifampicin-resistant TB**

### TB treatment is required for minimum of 6 months to complete and needs to be taken in daily basis to maintain a good success rate. How can youth engage in supporting daily DOT by TB patients in community/home for those under TB treatment?

## **Objective 6: To enroll 80% of eligible childhood contact and 50% of eligible vulnerable population for treatment of TB infection and strengthen implementation of infection control measures**

### TB found in children means that there is a nearby source of infection in family / close contacts. Similar TB in vulnerable population (e.g. Diabetes, HIV, past TB cases, marginalize and poor, elderly, smokers) need more focus to these populations. How can youth be engaged in addressing these issues at the community level and policy level. Objective 7: Address TB among PLHIV, diabetic, malnourished, smoker and other immunocompromised through a collaborative framework and multi-sectoral approach

### Addressed in question to objective 6

## Objective 8: To strengthen the health system and improve quality TB services under universal health coverage and ensure no affected family faces catastrophic costs due to TB by 2025

### Universal health coverage (UHC) aims to provide promotive, preventive, curative, rehabilitative and palliative health services to all citizens. It is essential to ensure that TB care and prevention is included in the UHC framework in Nepal. This needs strong political Commitment with an increased and sustained resource for TB services. How can youth engage and support in this process?

## Objective 9: To strengthen and scale up CSS, Civil Society Network engagement and ACSM in TB care and prevention

### How can youth engage to create enabling environments and advocacy for TB care and prevention (create vibrant community networks, linkages, partnerships, and coordination)

### How can youth be engaged in Planning, monitoring & evaluation (from bottom up levels)

### How can youth support in developing and engaging in ACSM activity?

| Provinces | Districts | Dates ?/NGOs | KAP |
| --- | --- | --- | --- |
| Province-1 |  |  |  |
| Province-2 |  |  |  |
| Province-3 |  |  |  |
| Province-4 |  |  |  |
| Province-5 |  |  |  |
| Province-6 |  |  |  |
| Province-7 |  |  |  |

Note: Yellow shaded are the selected districts for FGD

**Plan for Focus Group Discussion**

| **S.N.** | **District** | **Partners for FGD** | **Date for FGD** | **Name of FGD Lead/contact number** | **DTLO/contact number** | **RTCO/contact number** | **Remarks** |
| --- | --- | --- | --- | --- | --- | --- | --- |
|  |  |  |  |  |  |  |  |
|  |  |  |  |  |  |  |  |
|  |  |  |  |  |  |  |  |
|  |  |  |  |  |  |  |  |
|  |  |  |  |  |  |  |  |
|  |  |  |  |  |  |  |  |
|  |  |  |  |  |  |  |  |
|  |  |  |  |  |  |  |  |

Geo-targeting key and vulnerable populations

The following factors, social determinants and gender issues played a role in the geo-targeting of key and vulnerable sub-populations and selection of sites for conducting FGDs:

1. PLHIV, MSM, sex workers and transgender
2. Indigenous population and tribal groups
3. Prisoners and those in rehabilitation centres (IDUs)
4. Internally displaced persons, migrants and refugees
5. Urban poor including homeless, street children and garbage collectors
6. Factory workers, construction site labourers and seasonal workers

Methodology and Tools

Focus group discussions if conducted well can create an accepting environment that puts participants at ease allowing them to thoughtfully answer questions in their own words and add meaning to their answers. The aim of the facilitator should be to generate a maximum number of different ideas and opinions from as many different people in the time allotted.

- Number of participants

Ideally, the number of participants should about 10 and the range is between 8-12. Too many participants in a group might create more confusion and will be less productive. Gender balance is needed and the age of the participants is also important.

- Collect demographic information of participants

It is important to collect demographic information from participants since age, gender, or other attributes are important for correlation with focus group findings. Design a short half page form that requires no more than two or three minutes to complete. Administer it before the FGD begins. A consent form duly signed maybe required. Participants may be identified by names of fruits or flowers to give them a sense of confidentiality. Badges with pictures of fruits/flowers may be handed over to the participants which will be pinned on their chest. While documenting the responses from the participants, the same names of fruits/flowers should be used to identify them.

- Duration of FGDs

The duration of FGDs should be between 45 to 90 minutes and generally it has been observed that beyond period, most groups are not productive.

- Design of the questionnaire
- Focus groups should be structured around a set of carefully predetermined questions – usually no more than 10 and allow the discussion to be free-flowing.
- Normally, questions are short and to the point, open-ended, generally focused on one dimension, without any ambiguity and without causing any embarrassment.
- The questions are designed in a way that they cannot be answered with a simple “yes” or “no” answer.
- In the end, there should be an exit question to find out if there is anything else the participants wish to say.

Sample Questionnaire

1. Please tell us what do you understand about TB, including the symptoms of TB?

(*to understand the patient’s* *awareness about TB*)

1. In your opinion, who are the people most likely to get TB?

(*to know whether they know the risk factors of TB*)

1. What are the reasons that make the people with TB symptoms to seek immediate medical aid?

(*to understand the reasons for seeking urgent help*)

1. What are the reasons for delay in seeking medical aid?

(*to understand their ignorance, fear, stigma or other concerns)*

1. Ask the respondents to rank the reasons, 1-5 in terms of importance.

(*to understand the strongest reason for delay in reporting to health facility*)

1. What needs to change to make people seek urgent help?

*(to understand what barriers needs to be addressed by the NTP)*

1. If a person gets symptoms of TB, who is the first person he will approach to seek help?

(*to understand the health-seeking behaviour of a TB suspect*)

1. Do you know that TB diagnosis and treatment is free in the government health center?

If yes, who told you? Or from where you got the information?

(*to understand the impact of ACSM activities at the community level)*

1. Did you come to the HC daily to receive TB treatment for the entire period?

How many days' drug supply you got?

(*to monitor DOT practice in the HC)*

1. Are you satisfied with the TB services provided by the HC and how will you rate it on a scale of 1-5?

Use your fingers (1 is the worst, 3 is OK and 5 is best!

(*to understand the gaps in the TB health care delivery system*)

1. What are your suggestions to improve the services?

(*to get a “bottom-up” approach and practical feedback from the community level*)

1. Did the HC staff advice you to bring your close family members for testing after you were diagnosed with TB?

(*to understand the knowledge of HC staff on the importance of household contact investigation)*

1. During the treatment, did you have any problems?

(*to monitor treatment side-effects and the consequences*)

1. After the treatment was completed, did you feel better?

(*to highlight the benefits of DOTS and its effectiveness*)

Analysis of the data

There are two ways of analyzing the data collected from a FGD.

Using an Excel format:

1. Enter all responses in an Excel data sheet and group them to pre-arranged categories.

2. Chose the best categories for organizing the data, assign a number or letter to each category.

3. Then assign the number/letter of the category that best fits to each response on the sheet.

4. Group responses using the Excel sheet by the categories that have assigned to them.

5. If some responses do not fit a certain category, re-categorize or add another category.

6. Arrange categories from those with the largest number of responses to those with the least.

7. Repeat for each group.

As an alternative to the spreadsheet method described here, you can also use a manual approach to analyzing focus group data.

Using a Word format:

1. Make a copy of the responses from participants after it has been cleaned and labeled.

2. Cut responses into separate strips.

3. Categorize by sticking responses onto separate sheets of paper labeled with broad headings.

4. Re-categorize as indicated until you are satisfied with your groupings.

5. Complete the analysis in a Word document.

Final report of the FGD

Findings can be written up in a narrative format that includes:

- An executive summary,
- Background section,
- Methods used,
- Major findings,
- Conclusions, and
- Recommendations.

**Global Fund investment in building a Resilient and Sustainable System for Health (RSSH)**

A Joint Meeting of Focal Points from National TB Centre, National AIDS Centre and National Malaria Centre and the Writing Teams of the respective programmes will be held on 15/16 June 2017 to discuss the components of the RSSH. Some of highlighted sections below will be focused for initial discussion and others will be taken up after consultations with HIV/AIDS and Malaria Programs, to strengthen the Integrated Service Delivery strategy and package of activities aligned to the “Patient-Centred Care” approach.

**(1) Strengthen community responses and systems**

- CBOs can be strengthened to independently monitor, document and analyze the performance of health services to provide feedback to service providers;
- Communities & affected populations can conduct dialogue, advocacy & foster consensus at local/national levels to address discrimination, gender inequality and sustainable financing;
- Social mobilization, building community linkages, collaboration and coordination
- Capacity building of community sector groups, organizations and networks in a range of areas is necessary for them to fulfill their roles in service provision, social mobilization, monitoring and advocacy.
  - Strengthen the Health Facility Operations Management Committee
  - Strengthen the capacity of FCHVs to conduct outreach activities

**(2) Support RMNCAH**

- Developing a supportive policy and programmatic environment that enables the delivery of packages of integrated services for women, newborns, children and adolescents;
  - To support screening of women (30-60 years) for cervical cancer
  - To support screening of children with malnutrition
- Highly synergistic RMNCAH integrated services with justification (e.g., prevention and treatment of anemia, breastfeeding, mental health, malnutrition and cervical cancer screening and treatment);
- Integrating diagnostic services for different diseases within the same facility helps avoid duplication of investments in infrastructure and laboratory supporting systems, such as specimen transport, supply chain management and information systems.
  - To support specimen transport system
  - To support logistic management and information system

**(3) Strengthen in-country PSM systems**

- Strengthen the capacity of the national regulatory authorities that oversee the implementation of health products regulation overall, with a focus on often neglected areas including rational use and pharmacovigilance activities;
- Build the capacity of the procurement and supply chain workforce in quality assurance, procurement, storage and distribution;
- Develop improved information systems to track product stocks, disbursements, distribution and to analyze use rates enabling prediction of stock replenishment needs.

**(4) Leverage critical investments in HRH**

- HRH policies, governance and workforce planning/management;
- Pre-service education to address imbalances (e.g. increase the representation of women, ethnic minorities and rural practitioners in the health workforce);
- In-service training (e.g., updating health workers on new procedures and guidelines);
  - Develop SOP on Infection Prevention & Control
  - Conduct training of health workers on Infection Prevention & Control
  - Conduct training of all lab technicians on the use of diagnostic equipment (microscopes, Xpert) including basic maintenance and calibration.
- The Global Fund will consider funding interventions to improve retention and motivation, especially in rural and remote parts of the country.

**(5) Strengthen data systems & capacity for data analysis/use**

- Establishment, maintenance and strengthening of national HMIS, district health information system, mobile platforms for community level data collection and reporting;
- Training staff at all levels to use data to make informed management and program decisions and monitor program progress;
- Program reviews and evaluations including analysis, interpretation and use of programmatic and epidemiological data;
  - Joint TB Programme Review
  - Mid-term evaluation of the NTP
  - Operational Research
- National Health Accounts and sub-accounts;
- Civil registration and vital statistics system.

**(6) Strengthen and align to robust national health strategies & national disease-specific strategic plans**

- Activities that contribute to planning, developing and reviewing national health sector strategies, health systems-related strategies and sub-strategies;
- Activities at the local, district, regional and national levels aimed at: integrated planning, programming, budgeting and financing health and disease control programs;
- HRH-related costs, such as capacity building for policy makers.

(7) **Strengthen financial management and oversight**

- Public financial management strengthening:
  - Financing country action plans for public financial management;
  - Enhancing internal controls;
  - HRH-related activities, such as capacity building of auditing bodies and implementers.
- Routine financial management improvement:
  - Risk, assurance and treasury management directly at the grant level;
  - Introduction of tools and process development;
  - Capacity building directly related to Principal Recipient and sub-recipient grant implementers;
