# Supplementary material for: Community and stakeholder engagement in national priority setting and participatory research for HIV, Tuberculosis, and Malaria programs in Nepal
Source: Res Involv Engagem. 2026 May 22;12:69. doi: 10.1186/s40900-026-00907-3 (PMC13198034; doi:10.1186/s40900-026-00907-3)
Supplement: Supplementary file 4 — Supplementary material 4 [file 40900_2026_907_MOESM4_ESM.docx]

# **Guidelines for the Country Dialogue on** **Malaria for the Revision of the National Malaria Strategic Plan (2014–2025)** **and for** **Funding Request Development to the Global Fund** **Grant Cycle 6 (GC6)**

**Background**

Country dialogue is an open, inclusive, and continuous process that centers communities affected by malaria alongside implementers and decision-makers. It serves to:

- **Revise/refresh the NMSP (2014–2025),** including an elimination road map and transition actions, and
- **Identify & agree priorities** for the upcoming Global Fund funding request (and alignment with other partners).

CCM Nepal convenes a **Malaria Task Team** (under the CCM Task Team arrangement) with EDCD/MoHP, provincial/local authorities, CSOs, private sector, networks, and development partners. The Task Team:

- Forms **disease-specific sub-teams;**
- Drafts **ToRs** for national/international consultants;
- Plans/schedules consultations; and
- Coordinates a **costed writing plan** and budget/resource mapping.

## **Country Dialogue Process (Overall Flow)**

**Sequencing**

1. **Community & local consultations (endemic palikas in priority districts)**
2. **Provincial consultations (all 7 provinces)**
3. **Gap analysis & donor/partner meeting**
4. **Federal-level country dialogue (synthesis & prioritization)**
5. **Writing & thematic reviews**
6. **Validation & final endorsement**

**Priority geographies** (as per your base list; may be refined by latest micro-stratification/EDCD): Udayapur, Morang, Sankhuwasabha, Dhanusha, Saptari, Chitwan, Kavrepalanchok, Nawalparasi East, Kaski, Kapilbastu, Rupandehi, Bardiya, Mugu, Dailekh, Dadeldhura, Kanchanpur, Kailali.

**Stakeholders Engagement Plan**

To ensure inclusivity throughout the cycle:

1. **Prepare a standard EDCD presentation (achievements, challenges, revised goals/targets).**
2. **Use standard feedback forms (by thematic area) for provincial/federal rounds.**
3. **Provide a common report template so all teams return comparable outputs.**

## **Community & Local-Level Consultations**

**Objectives**

- **Surface local service gaps, barriers (including HRG), and opportunities for elimination.**
- **Validate micro-stratification and tailor interventions for hard-to-reach, migrants, forest/brick-factory/construction workers, uniformed services, pregnant women/children <5, and disaster-affected populations.**
- **Define local co-financing and roles for palikas (e.g., LLIN top-ups, outbreak response, vector-control logistics).**

**Participants (illustrative)**

Palika leadership/health units; local leaders/security; health office malaria focal person; public & private providers; FCHVs; affected persons; CSOs; women’s/youth groups.

**Methods & Tools**

- FGDs & KIIs using standardized guides (Annex A/B) covering: knowledge, prevention (LLIN/IRS/LSM), diagnosis (RDT/microscopy), treatment (incl. PQ adherence), community testing, surveillance/MDIS, HMIS/DHIS2, quality control, and SWOT.
- Strive for gender balance (≈50% women where feasible) and KP inclusion.
- Outputs (≤10 days): short reports (issues, solutions, priorities, indicators) with attendance, photos, and participant selection logs.

**Provincial Consultations (7 Provinces)**

**Objectives**

- Convert local findings into provincial priorities aligned to NMSP pillars and elimination targets.
- Map partner/service coverage (public/private), HR/logistics/lab capacity, and surveillance performance.
- Propose high-impact interventions and co-financing prospects for the funding request.

**Participants**

MoSD; Provincial Health Directorate; health offices from endemic districts; provincial

lab/training/logistics centers; district/provincial hospitals; CSOs; private sector; EDPs. Outputs (≤2 weeks each): provincial recommendation memo (priorities, investments, risks/mitigation).

## **Federal-Level Country Dialogue**

**Purpose**

Synthesize recommendations; finalize **national malaria priorities** for the funding request and NMSP update.

**Core agenda (per GF guidance)**

- **Elimination focus: foci investigation & response; interrupt transmission; lab quality; universal access to case management.**
- **Vector control: LLIN/IRS per micro-stratification; LSM where appropriate; quality/acceptability.**
- **Surveillance as a core intervention: 24-hour notification; MDIS functionality; classification (indigenous/imported); outbreak response timelines and HR.**
- **Community testing & treatment: policy enablers, trained cadres, RDT/medicine availability, data tools, referral/voucher mechanisms.**
- **Private sector engagement: notification, QA of RDTs/drugs, reporting to HMIS/DHIS2.**
- **Cross-border & imported malaria: joint mechanisms, migrant pathways, case tracking.**
- **Human rights & gender: access for migrants/hard-to-reach; grievance & protection.**
- **Data systems & quality: DHIS2 tailoring, supportive supervision, data use for action.
  Outputs: prioritized investment list; co-financing map; items feeding the writing matrix.**

**Gap Analysis and Donor Meeting**

**EDCD compiles a preliminary gap/costing analysis (NMSP & elimination roadmap), noting reduced GF envelope risks and GoN allocations; revises after donor meeting. A multi-partner session (EDPs, PR/SRs, WHO, etc.) aligns investments and identifies GF-appropriate priorities vs. GoN/other funding.**

**Writing Process & Thematic Team Meetings**

- **Lead writer: international consultant (with WHO/national consultant support) + Writing Team (EDCD/Task Team/PR-SRs/CSOs/private sector).**
- **Thematic sub-teams meet as needed on: supply chain; community testing & treatment; surveillance/MDIS & DHIS2; microscopy/RDT QA/QC; asymptomatic/sub-microscopic malaria; private-sector engagement; vector control/LLIN & IRS; cross-border; HRG/HRG-gender; labs/QC.**
- **Maintain a Writing Tracker & Evidence Matrix (maps each priority to evidence, NMSP target, and GF question).**

**Communications & Feedback**

**Use CCM website/social media + hotline/email to solicit and log inputs nationwide; route compiled feedback into provincial/federal dialogues and the writing tracker.**

**Validation & Endorsement**

- **Validation workshop confirms upward integration (community→province→federal) and checks HRG/gender, private sector, surveillance, and cross-border content.**
- **Endorsement meeting (CCM) finalizes submission; share final materials with CCM members, PRs/SRs, and dialogue participants for transparency.**

**Annex A: Criteria for Focus Group Discussions FGD) at the community Level**

1) Objectives of the Focus Groups Discussions (FGD) to be organized in Thirteen Endemic Local Palikas of Thirteen districts.

- To identify the strengths, weaknesses, opportunities and threats of the existing malaria interventions in the community in order to document the current gaps that need to be addressed to further improve the program.
- To understand and document the perception and needs of the target beneficiaries, as well as their acceptance towards the interventions.
- To identify what needs to be improved in the current malaria program, the challenges to be taken into account and yet overcome, and the potential clarification needed regarding the roles of all the stakeholders. Such information will provide evidence/basis for future modifications of the malaria interventions and activities in order to make them more complementary, inclusive, effective and efficient.
- To make recommendations based on community consultation.

2) Criteria for selection of participants:

- 50% Male / 50% Female at community level when possible except for FCHVs
- Female laboratory trainees and nurses should be encouraged to participate
- Health Workers / Malaria Infected/Affected Population
- Communities Stakeholders,
- Public sector / Private sector
- Children (who attended or not malaria school programs)
- Risk groups (migrant, forest, brick factories workers, etc.)

3) Topics/Components to be discussed:

- Knowledge of Malaria
- Prevention of Malaria
- Diagnostic and Management of Malaria
- Treatment of Malaria
- Reporting system
- Quality control
- Perceptions on LLINs and IRS, Use of LLINs in the Communities
- Community Level Testing
- Surveillance system

**SWOT Analysis:**

- Strengths of the current activities / way of addressing malaria
- Weaknesses of the current activities / way of addressing malaria
- Opportunities for improving/expanding activities, for new activities/interventions
- Threats that can affect the future activities / way of addressing malaria

**Number of Participants:** 15 max per group

**Duration:** 60-90 minutes

**Introductory Remarks**

Namaste, my name is *........... (moderator*) and my colleagues are*………… (note takers and officials).* We are conducting a focus group discussion on malaria in this Community. The information we are collecting will help in advising donors and programme implementers on better ways of designing malaria interventions. You have been selected to participate in this discussion because we feel your views will help in understanding the real issues in malaria in this community/district. We therefore, kindly request you to share your honest experiences/opinions on these issues. Participation in this discussion is voluntary. You are free not to contribute to any issues you feel uncomfortable with. However, we wish to assure you that the information you give us shall be kept confidential and will only be used for purposes of this review/analysis. The information will not be linked to any participant. Our discussion will last approximately 45-60 minutes during which everybody will have an opportunity to contribute. So please, if you agree to participate you will be required to **speak one at time** and feel free to express your views freely even if you feel that they are different from what other participants think.

I also wish to kindly request you to allow me tape-record this discussion so that I can capture everything we discuss.

Do you have any questions or comments before we proceed?

**Instruction to the Moderator:**

- In case of any questions, please try to address them before proceeding.
- In case participants refuse to be tape-recorded, ignore the recorder and proceed with the discussion as the note-taker writes down verbatim as much as she/he can.

**INFORMATION**

| **District** |  |
| --- | --- |
| **Municipality/ Rural Municipality** |  |
| **Total Number of participants:**  **High risk ward:**  **Moderate risk ward:**  **Low risk ward:**  **No risk ward:** |  |
| **Participant details to be attached separately: name/occupation** |  |
| **Highest educational qualification of the group** |  |
| **Least educational qualification of the group** |  |
| **Language of discussion** |  |

**ANNEX B: Questionnaire for focus group discussion, key informant interview.**

|  | **TOPIC** | **MAJOR QUESTIONS** |
| --- | --- | --- |
| **1** | **Malaria in the district** | **Q:** Warm up: Is Malaria an issue for you/ for your district? Why? |
| **2** | **Knowledge of Malaria** | **Q:** What can you tell us about malaria in this district?  (Probe for perception of malaria in relation to other health problems)  **Q:** How serious is the problem of malaria in this district?  **Q:** What are the causes of malaria?  **Q:** How do people manage malaria or when they get fever?  **Q:** Any specific wards /population/gender/age groups more affected.?  **Q:** What human-rights, gender and age-related barriers exist that affect the people's use of these services? |
| **3** | **Prevention of malaria** | **Q:** How are people in this district protected from getting malaria? (Probe for bed nets, IRS, education program, breeding site destruction/reduction)?  **Q:** Where do you get LLIN/ITN in this district? (Probe for the cost if any)  **Q:** When was the last time the LLIN was received? How many, how many persons in the household?  **Q:** Inquire about the LLIN use?  **Q:** What are the general feelings of people in this district about LLIN and IRS? (Probe for acceptability, availability, affordability, accessibility, longevity and desirability of LLIN and IRS)  **Q:** Are there obstacles/issues with using LLIN as efficient prevention?  **Q**: How can we improve the coverage and use of LLIN in the community?  **Q:** How often IRS spraying is done? Have IRS ever been conducted in your house?  **Q:** Any malaria outbreak/cases reported in your community/ward/palika?  **Q:** Do you think the increasing access to diagnosis and treatment by HCWs in hard to reach, remote community will be helpful? |
| **5** | **Diagnosis of malaria** | **Q:** What is used for malaria diagnosis: RDT or microscopy? Are the health staff/workers able to diagnose malaria using RDT or malaria? If no, how do they refer/manage patients?  **Q:** Are there any challenges to diagnose malaria? (Probe for RDT stock-out, lab tech availability, quality of RDT)  **Q:** Where do people go to visit for malaria care- diagnosis and treatment?  **Q:** Are you aware of the voucher system or other means to refer patient from community to health facility? Explore  Q: Do you think malaria RDT testing at the community will be helpful to find more cases?  **Q:** Do you think there are malaria cases who do not go to the health facility for diagnosis and care? |
| **6** | **Treatment of malaria** | **Q:** How is malaria treated in this district health facility? (Probe for different types of treatment and reasons, assess competency of health workers)  **Q:** How do you ensure that people take the correct and full treatment? Is there any follow-up? If not, what are the obstacles?  **Q:** Do you encounter any challenge in providing treatment (patient adherence on primaquine for 14 days)  Q: Are there any report of the adverse event noted by the patient while on malaria treatment (primaquine, chloroquine) |
| **7** | **Surveillance and response** | **Q:** How is malaria case reported once diagnosed in the health facility?  Q: If MDIS used, what are the issues with MDIS reporting?  **Q:** What should be done to ensure that cases are notified within 24hrs?  **Q:** Are there any issues with recording and reporting of malaria cases using HMIS/DHIS system? Do you encounter any challenge to timely report malaria disease information to HMIS/DHIS2?  **Q:** Do private facilities report or refer cases to the DPHO? Why not?  **Q:** What do you propose to get this done? How do we encourage private sector to report?  **Q:** How do you respond to a malaria cases? Ask about how, indigenous/imported classification is done, who verifies the final classification.  **Q:** Have you been involved in malaria case response (outbreak response), If YES_ how was it done, timelines, HR involved, community participation, support from provincial, central level.  **Q:** Anyone ever conducted or supervised IRS operation? Did he receive any training? |
| **8** | **Quality control** | **Q:** Are you aware of the quality control system in place for microscopic slides confirmation?  **Q:** How can we improve the quality control system for microscopic slides confirmation?  **Q:** How do you ensure that RDT and drug provided in private facilities are compliant? What do you propose? |
| **9** | **Open topics if time permits**  **SWOT** | **Q:** What are the strengths of the current activities / way of addressing malaria. Probe systematically from prevention (vector control), diagnosis and treatment, surveillance and outbreak response.  **Q:** What are the weaknesses of the current activities / way of addressing malaria.  **Q:** Are there opportunities for improving/expanding activities, for new activities/interventions (introduce new activities and ask for feedback:  **Q:** Are there threats that can affect the future activities / way of addressing malaria : |

**Annex C: Federal/ Provincial consultation**

National consultant for the GF proposal shall lead and facilitate the national consultation in coordination with Malaria Task Team

- Sharing of compiled report/findings/recommendations from community/Provincial discussions and SWOT analysis,
- GoN contribution/commitment in commodities procurement, quality assurance of commodities, diagnosis and treatment, or new interventions to increase coverage/investigation/surveillance at the community level, etc,
- Selection of elimination interventions (within the NMSP Elimination Framework) to be covered by the global fund grant vs government or other donors.

**Selection of participants**

Selection of the participants for Federal/Provincial Country Dialogue: CCM Nepal Members representing from their respective constituencies will coordinate with their networks to select the participants through inclusive and democratic process. For government participants CCM Nepal Chair and EDCD Director will provide nominations for the participants. CCM Nepal member representing from EDP constituencies will select the participants from EDP’s and International NGO in consultation with EDCD. WHO, PR, SRs, LFA, and other relevant stakeholder will be invited as suggested by Malaria Task Team.
