# Supplementary material for: Community and stakeholder engagement in national priority setting and participatory research for HIV, Tuberculosis, and Malaria programs in Nepal
Source: Res Involv Engagem. 2026 May 22;12:69. doi: 10.1186/s40900-026-00907-3 (PMC13198034; doi:10.1186/s40900-026-00907-3)
Supplement: Supplementary file 5 — Supplementary material 5 [file 40900_2026_907_MOESM5_ESM.docx]

**Supplementary File 2**

**National Consultation Process Stakeholder Engagement and Sampling Strategy**

**Introduction**

This supplementary document describes the purposive and snowball sampling strategy used in Nepal’s national consultation process for HIV, TB, and Malaria programs. The consultation process was designed to be highly inclusive and iterative, linking stakeholder engagement directly to (i) the development of updated National Strategic Plans (NSPs) for the three diseases, (ii) the preparation of Global Fund funding applications for 2021–2024, and (iii) the prioritization of activities under USAID-funded PEPFAR.

The narrative below highlights how a broad range of stakeholders with special emphasis on historically marginalized groups and key/vulnerable populations were engaged through multiple rounds of dialogue. It also illustrates how the process continued beyond the point of traditional data saturation, using repeated inclusive engagements for validation and consensus-building so that priorities in NSPs and funding applications were both evidence-informed and nationally owned.

**Sampling Strategy: Purposive and Snowball**

**Purposive Sampling**

From the outset, the Country Coordinating Mechanism (CCM) and health authorities identified the stakeholder categories to be included. Purposive sampling ensured representation from the most information-rich constituencies across sectors and levels. Stakeholders were selected to cover:

- Government: national (MoHP, Divisions, Centers), provincial (social development ministries/health directorates), and local (municipal health offices, public facilities).
- Civil society & community-based organizations (CBOs): including networks of key populations and affected communities.
- Development partners & donors: multilateral, bilateral, and technical agencies.
- Implementing partners: Principal Recipients (PRs) and Sub-Recipients (SRs) of the Global Fund and other implementers.
- Private sector: private hospitals/clinics, pharmacies, professional associations.
- Academia & public health institutes: research and training bodies contributing evidence.
- CCM members and leadership: for oversight, harmonization, and endorsement.

**Key and vulnerable populations (three diseases):**

- HIV: PLHIV, FSW, MSM, TG, PWID, migrants and spouses, and incarcerated populations.
- TB: TB survivors; DS-TB and DR-TB patients; marginalized caste/ethnic groups (e.g., Mushar community); elderly/geriatric populations; prisoners; refugees; factory workers; under-served urban slums and remote rural settlements.
- Malaria: seasonal migrants and mobile populations; forest-goers and agricultural laborers; communities in malaria-endemic geographies (notably the Terai belt and forested/border districts with India); refugees in malaria-prone camps; pregnant women in endemic areas.

This intentional recruitment guaranteed that the consultations included those most affected and most knowledgeable about service gaps, access barriers, human-rights considerations, and contextual feasibility.

**Inclusion/Exclusion (operational summary):**

- Included: stakeholders with direct roles in or influence over program design, delivery, oversight, demand-generation, or advocacy for HIV/TB/Malaria; individuals with lived experience (patients/survivors); community leaders and peer educators; provincial/local officials responsible for execution.
- Excluded: actors with no material linkage to the three programs; participants unable to provide informed input (e.g., lack of exposure to services/policies), unless present as caregivers/guardians or community proxies in structured group settings.

**Snowball Sampling**

A snowball approach was run in parallel to widen outreach and reach hidden, remote, or stigmatized constituencies. Initial participants and focal organizations (e.g., national key population networks, provincial civil society coalitions, FCHV clusters) were encouraged to nominate additional stakeholders. Examples include:

- Key population networks connecting to peer leaders across all seven provinces for HIV consultations.
- TB contacts identifying DR-TB patient groups, prison health focal points, and factory worker cohorts.
- Malaria focal points linking to seasonal migrant hubs, forest worker cooperatives, and Terai border communities.

This chain-referral created an expanding pool of informants, improving regional, gender, age, and vulnerability diversity beyond the original sampling frame. The purposive + snowball combination provided both breadth (coverage of predefined critical groups) and depth (granular voices surfaced through referrals).

**Recruitment & Consent:**

- Invitations were issued via official letters, email/phone, and network announcements.
- For virtual engagements (COVID-19 period), verbal/recorded consent was taken at the start of calls; for in-person, written sign-in with consent language was used.
- Participation was voluntary, with ability to opt out; privacy was protected by aggregating inputs and omitting personally identifying details in reports.

**Integration with National Strategic Plan Development (Umbrella Process)**

A core design choice was to make the NSP revision/development the umbrella for all engagement. Early resolutions (late 2019) embedded NSP review into the same platform that discussed funding.

**Consequently, most consultations served dual purposes:**

1. Reviewing existing NSPs (HIV NSP 2016–2021; National TB Strategic Plan 2016–2021; National Malaria Strategic Plan 2014–2025) to identify gaps, equity issues, and targets to carry forward or recalibrate; and
2. Co-creating new strategic directions including service packages, health-systems enablers (e.g., RSSH, data systems, human rights and gender), and sub-national operational adjustments.

**Key features:**

- Sequential layering: community insights (FGDs, KP dialogues) → provincial synthesis/feasibility checks → national thematic deep-dives (e.g., human rights/gender, PPM for TB, private sector for diagnostics) → national prioritization & validation.
- Virtual continuity during COVID-19: Zoom/Meet, phone trees, messaging apps; multilingual facilitation; simple participation guides to reduce digital barriers.
- Direct policy translation: outputs were mapped into results frameworks, NSP objectives, milestones/targets, and monitoring indicators.

By the end, all three NSPs were grounded in extensive stakeholder input, with clear lines of sight from community statements to strategy text and indicators.

**Integration with Global Fund Funding Application (2021–2024)**

Within the NSP-led process, development of Global Fund proposals was embedded as an aligned output.

**Governance and operations included:**

- Core Task Team (Feb 2020) and disease-specific task teams with diverse composition (government program directors, CSOs, KP reps, development partners, PR/SR implementers).
- Consultative activities across levels: community meetings, FGDs, provincial workshops, and national technical consultations to specify interventions, coverage targets, and budgets.
- COVID-19 adaptations: standardized guides for virtual consultation, flexible timing to accommodate connectivity, and iterative circulation of draft matrices for asynchronous feedback.

**From input to proposal content:**

- Community and provincial inputs were triangulated with routine program data, surveys, and costing analyses to draft modular intervention packages (e.g., case-finding for TB, prevention and differentiated care for HIV, vector control and surveillance for malaria).
- Prioritization workshop (Aug 2020): multi-stakeholder review to rank interventions by impact, equity, feasibility, and funding gap.
- Validation meeting (Aug 2020): consensus confirmation on interventions, budgets, and performance frameworks.
- CCM endorsement (Aug 2020): formal approval prior to submission.

The proposals were recognized as nationally owned, transparently prioritized, and responsive to community-voiced needs.

**Integration with PEPFAR HIV Support Prioritization (USAID)**

The process also aligned PEPFAR HIV investments with national priorities under the NSP umbrella:

- PEPFAR/USAID participated in task teams and provincial/national consultations, ensuring visibility of ongoing support and required complementarities.
- Funding alignment meetings (July 2020): partners agreed on division of labor to avoid overlap and close coverage gaps. Illustratively, GF supported PLHIV, PWID, and migrants; PEPFAR concentrated on FSW and MSM/TG programming; another partner supported prison HIV services.

Result: a shared roadmap where PEPFAR’s Country Operational Plan complemented GF grants and the NSP’s equity/impact agenda.

**Breadth of Inclusion & Focus on Marginalized Groups**

- Government: Federal program managers and policy units; provincial health leaders; municipal health offices providing feasibility and resource insights.
- Civil society: National/local NGOs and CBOs; advocacy and rights groups; faith-based actors.
- Key/vulnerable populations: PLHIV, TB survivors, FSW, MSM, TG, PWID, migrants, prisoners; for malaria, Terai/border communities, seasonal migrants, forest-goers, and pregnant women in endemic areas.
- Frontline/community workers: FCHVs, peer navigators, outreach workers.
- Private sector: hospitals/clinics, labs, pharmacies; employers for TB screening/PPM.
- Development partners: WHO, UNAIDS, USAID/PEPFAR, UN agencies, and the Global Fund country team (support/observer roles).
- Academia/research: methods review, evidence synthesis, and indicator alignment.

Not tokenistic: Many marginalized participants engaged in multiple rounds (early needs identification, priority-setting, validation), some serving on task teams, building ownership and trust.

**Quality Assurance, Bias Mitigation & Ethics**

- Facilitation & standardization: common agendas, prompts, and note-taking templates ensured comparability across sessions; co-facilitation with KP/community leaders improved trust and candor.
- Language & access: local language facilitation; telephone-based participation for low-bandwidth areas; small stipends/airtime where appropriate (per policy).
- Confidentiality: group norms set at the outset; data recorded in aggregate; sensitive disclosures anonymized.
- Bias mitigation: purposive lists deliberately included divergent voices (e.g., rural/urban, female/male/other gender identities, younger/older age bands). Snowball referrals were checked to avoid homophily (same-group clustering).
- Triangulation: qualitative inputs cross-checked with program data, epidemiologic trends, costing, and prior evaluations to reduce single-source bias.
- Ethical posture: the process followed government consultation norms; sessions began with informed consent scripts; participation was voluntary; no personal identifiers appear in outputs.

Saturation beyond “enough”: once themes repeated, additional rounds were used to validate, refine, and prioritize deliberately exceeding saturation to ensure consensus and accuracy before integration into NSPs and funding requests.

**From Consultation to Decisions (How inputs were used)**

- NSPs: Inputs mapped to strategic objectives, intermediate results, interventions, and equity/HRG safeguards; indicators and targets adjusted for sub-populations (e.g., DR-TB, MSM/TG, mobile populations).
- Global Fund proposals: Prioritized intervention lists and coverage targets converted into modular budgets, performance frameworks, and implementation arrangements (PR/SR roles, sub-national roll-out).
- PEPFAR alignment: Complementarity documented in partner matrices so that no key population or geography remained unfunded.

**Summary Tables**

**Table 1. Categories of Participants Consulted**

| Category | Description of Involvement |
| --- | --- |
| Government Officials | MoHP (federal), provincial health/social development ministries, municipal health offices; leadership, data, and policy inputs at every tier. |
| Community Members | PLHIV, TB survivors, malaria-affected families, community leaders; shared lived experiences and access barriers. |
| Civil Society (CSOs/CBOs) | National/local NGOs and advocacy groups; surfaced on-the-ground challenges and innovations. |
| Key Population Networks | PLHIV networks; PWID (e.g., Recovering Nepal); FSW organizations; LGBTIQ+ federations; migrants’ networks. |
| Development Partners | USAID/PEPFAR, WHO, UNAIDS, UNDP, others; technical inputs and funding alignment. |
| Implementing Partners | Global Fund PRs/SRs; academic/public health institutes; insights on feasibility and performance. |
| Private Sector | Private hospitals/clinics, labs, pharmacies, employer groups; TB PPM, diagnostics, and referral pathways. |
| CCM Members/Leadership | Oversight of inclusivity, transparency; final endorsement of proposals. |

**Table 2. Key Stakeholder Groups & Populations (with malaria geographies)**

| Stakeholder/Population | Representation & Inclusion |
| --- | --- |
| PLHIV | National PLHIV networks; inputs on ART access, stigma reduction, differentiated service delivery. |
| TB Survivors / Patients (DS/DR-TB) | Groups across provinces; adherence, stigma, infection control, social support needs. |
| FSW | Community organizations; prevention, STI services, legal/rights barriers. |
| MSM/TG | LGBTQ+ federations and youth KP consultations; stigma-free services, prevention, mental health, TG-specific needs. |
| Migrants & Spouses | Pre-departure/pre-return services, cross-border linkages, continuity of ART/PrEP. |
| Prisoners | HIV/TB screening and treatment continuity; infection control in closed settings. |
| PWID | Harm reduction (OST, needle/syringe), HIV/HCV co-infection, continuity of care. |
| Elderly (TB) | Geriatric screening/case-holding; co-morbidities. |
| Marginalized caste/ethnic groups | e.g., Mushar communities; equity-focused outreach and nutritional support. |
| Malaria-affected & mobile populations | Terai belt, forested/border districts, seasonal migrants, forest-goers, pregnant women in endemic zones; nets, IRS, testing/treatment, surveillance. |
| FCHVs & Mothers’ Groups | Community education, contact tracing, adherence support, vector control uptake. |

**Table 3. Timeline of Major Consultations & Decision Points (2019–2020)**

| Date(s) | Consultation / Event | Key Outcome / Decision |
| --- | --- | --- |
| Dec 2019 | CCM meetings (108th; Exec Committee) | Launch of national dialogue; NSP review embedded; August 2020 submission window targeted; Core Task Team to be formed. |
| Jan 2020 | Provincial civil society & KP consultations (all provinces) | Grassroots recommendations for NSP updates; initial funding priorities; engagement strengthened across provinces. |
| Jan–Feb 2020 | NSP preparatory meetings and thematic set-up | Thematic groups/consultants mobilized (HIV/TB/Malaria); data/evidence assembly plans agreed. |
| Feb 2020 | Community FGDs (TB/Malaria); CRG workshop (Malaria, 26 Feb) | Rich qualitative data on gaps/barriers; CRG concepts introduced; participants primed for broader virtual engagement. |
| 12 Feb 2020 | CCM Executive Committee | Core Task Team formed to coordinate dialogue and inputs. |
| Mar–Apr 2020 | Task Team formation; virtual consultation guides | Disease-specific task teams constituted; COVID-19 pivot to remote engagement. |
| Apr–May 2020 | Virtual community consultations (esp. HIV) | Province-specific KP recommendations captured despite lockdowns. |
| May–Jun 2020 | Provincial government consultations | Provincial feasibility and ownership confirmed; sub-national priorities integrated. |
| Jun–Jul 2020 | National thematic consultations (RSSH, HRG, PPM, private sector, youth KP) | Cross-cutting priorities refined; systems and equity actions strengthened. |
| Jul 2020 | Funding alignment (HIV) meetings (21 & 23 Jul) | GF/PEPFAR division of labor agreed; overlap minimized; gaps filled (e.g., prisons). |
| 2 Aug 2020 | National prioritization workshop | Ranked national priorities for inclusion in proposals/NSPs. |
| 20 Aug 2020 | Validation meeting | Final review and consensus on strategy/interventions/budgets. |
| 24 Aug 2020 | CCM endorsement (113th) | Formal approval for GF submissions; accountability reinforced. |

**Iterative Process & “Beyond Saturation” Rationale**

The process moved through successive rounds—community → provincial → national/thematic → prioritization → validation—each building on the former. Once themes began repeating, the team intentionally continued engagement to validate, refine, and secure consensus rather than stopping at conventional saturation. This improved:

- Accuracy: cross-checking with data and costing.
- Legitimacy: broad-based endorsement of difficult trade-offs.
- Equity: assurance that marginalized voices were not diluted in the final translation to strategy and budgets.

**Limitations & Mitigations (for transparency)**

- Digital divide (virtual period): mitigated with phone-in options, smaller group timings, local facilitation, and post-session callbacks.
- Selection bias risk: offset by snowball expansion, geographic rotation, and peer-led recruitment.
- Social desirability bias: minimized via peer facilitation, anonymized note-taking, and separate safe-space sessions for sensitive topics (e.g., SOGIESC, drug use, sex work).
- Time constraints: addressed by rolling submissions of written feedback and rapid synthesis memos to keep drafts moving.

**Conclusion**

By centering the NSP revision and embedding Global Fund proposal development and PEPFAR alignment within it, Nepal executed a nationwide, inclusive, and iterative consultation that amplified marginalized voices, harmonized funding streams, and translated inputs into policy and budgets. The purposive + snowball approach, coupled with validation beyond saturation, produced credible, consensus-driven strategies and funding requests with strong national ownership.
