# Supplementary material for: Community and stakeholder engagement in national priority setting and participatory research for HIV, Tuberculosis, and Malaria programs in Nepal
Source: Res Involv Engagem. 2026 May 22;12:69. doi: 10.1186/s40900-026-00907-3 (PMC13198034; doi:10.1186/s40900-026-00907-3)
Supplement: Supplementary file 6 — Supplementary material 6 [file 40900_2026_907_MOESM6_ESM.docx]

**GUIDANCE FOR VIRTUAL COUNTRY DIALOGUE PROCESS FOR NATIONAL STRATEGIC PLAN (NSP) DEVELOPMENT/REVISION AND GLOBAL FUND GRANT CYCLE 6 FUNDING APPLICATION**

1. **Introduction**

This guidance is developed to support all stakeholders including government agencies, civil society organizations (CSOs), community and key population (KP) networks, and technical and development partners in effectively engaging in the Country Dialogue Process for the development and/or revision of National Strategic Plans (NSPs) and the Global Fund Grant Cycle 6 funding application.

Given the evolving context of public health emergencies and mobility restrictions (e.g., COVID-19), the Country Coordinating Mechanism (CCM) Nepal decided to conduct the process virtually and/or in hybrid mode to ensure inclusivity, safety, and timely submission within the Global Fund window for Grant Cycle 6.

1. **Role of the CCM Secretariat**

The CCM Secretariat will:

- Lead and coordinate the entire country dialogue process.
- Provide technical and logistical support to all constituencies (government, CSOs, KP networks, private sector, and partners).
- Ensure timely communication, documentation, and adherence to Global Fund and national guidance.
- Circulate official templates, facilitation tools, and reporting formats.
- Manage resource disbursement and ensure transparent budget utilization for constituency consultations.
- Compile all consultation reports for submission to the Disease-Specific Task Teams and the CCM Nepal Executive Committee.

Each constituency (e.g., government programs, KP networks, technical partners) will assign a focal person to coordinate with the CCM Secretariat.

1. **Process Overview**

The **Country Dialogue Process** will follow multiple stages:

1. **Community and Key Population Consultations**
   - Led by national and sub-national community networks and organizations representing people living with or affected by the three diseases.
   - Aim: gather priorities, challenges, and proposed interventions for inclusion in NSPs and funding requests.
   - Supported technically and financially by the CCM Secretariat and Task Teams.
2. **Stakeholder and Partner Consultations**
   - Include government, technical partners, development partners (EDPs), civil society, and private sector representatives.
   - Aim: review programmatic progress, funding gaps, and opportunities for alignment with the national strategies and donor priorities.
3. **Provincial/Regional Consultations**
   - Organized in all seven provinces with participation from health directorates, provincial ministries, municipal representatives, and CSOs.
   - Aim: validate local priorities and align provincial perspectives with national NSP frameworks.
4. **Federal-Level Dialogue**
   - Brings together national, provincial, and community recommendations.
   - Aim: finalize priority areas, co-financing commitments, and investment strategies for Global Fund and other donors.
5. **Conducting Virtual Consultations**
6. **Virtual Modality**

Depending on the context, consultations will be held using Zoom, Microsoft Teams, Skype,

WhatsApp, Viber, Messenger, or telephone conferencing. Where in-person meetings are unavoidable, hybrid modalities will ensure inclusion while observing public-health precautions.

1. **Facilitation**

Each consultation will:

- Follow a structured agenda and reporting format shared by the CCM Secretariat.
- Be facilitated by designated Task Team members or trained moderators.
- Include a note-taker and, when feasible, a timekeeper.
- Maintain inclusivity and gender balance in participation.

1. **Ground Rules**

To ensure effective engagement:

- Begin each meeting with clear objectives and participant introductions.
- Respect turn-taking and speaking time; keep microphones muted when not speaking.
- Encourage diverse voices, particularly from marginalized groups.
- Summarize key points at the end and confirm consensus on recommendations.
- Seek informed consent before recording any session or sharing screenshots.

1. **Documentation, Recording, and Reporting**

Each focal person or facilitator must submit the following within five working days of the consultation:

- Meeting invitation or agenda.
- Participant list with gender, organization, and constituency.
- Screenshots, photos, or signed attendance (as applicable).
- Summary report capturing:
  - Key issues, gaps, and priorities discussed.
  - Recommendations aligned with NSP objectives.
  - Challenges and follow-up actions.

All reports must be emailed to the CCM Secretariat for compilation and archiving.

1. **Logistical Support and Budgeting**

- The CCM Secretariat will provide logistical support (communication, data packages, facilitator honoraria, documentation costs).
- Constituencies should submit a consultation plan with expected participants, modality, and budget.
- Financial support covers essential expenses only, no allowances beyond actual costs.

1. **Timeline for Consultations**

| **Activity** | **Lead Responsibility** | **Timeline** |
| --- | --- | --- |
| Circulation of Virtual Consultation Guide | CCM Secretariat | 10 April 2020 |
| Submission of Consultation Plans by Constituencies | CSOs, KPs, Government, Partners | 15 April 2020 |
| Completion of Civil-Society & KP Consultations | Constituencies | 25 April 2020 |
| Submission of Reports to CCM Secretariat | All Constituencies | 30 April 2020 |
| Government & Thematic Consultations | Task Teams | May–June 2020 |
| Federal-Level Country Dialogue | CCM Secretariat & Task Teams | June 2020 |
| NSP Revision and Funding Application Drafting | Writing Team & Consultants | June–July 2020 |

*(Note: The timeline will be updated for Grant Cycle 6 as per Global Fund’s call for proposals.)*

1. **Application Writing Process**

- Task Teams (HIV, TB, Malaria, and RSSH) including representatives from Government, CSOs, PRs, INGOs, EDPs, and KP networks will jointly work with national and international consultants.
- Writing Teams will integrate the outcomes of consultations into the NSP Revisions and Funding Requests.
- Thematic working groups (PSM, Prevention, Treatment, SI, Gender, Human Rights, RSSH, etc.) will convene as needed.

1. **Validation Workshop**

Before submission:

- A Validation Workshop will be organized to ensure that priorities and recommendations from all levels (community, provincial, and federal) are reflected in the draft NSP and funding request.
- Participants will review, validate, and endorse the consolidated draft for finalization.

1. **Endorsement Process**

Following validation:

- CCM will organize an Endorsement Meeting with all key stakeholders.
- The final NSP and funding application will be shared with CCM members, Principal Recipients, and dialogue participants for transparency.
- Once submitted to the Global Fund, CCM will disseminate the final version to all constituencies for reference and accountability.

**Annex I: Facilitation Guidance for Virtual Consultations**

**Key Principles for Virtual Facilitation:**

1. Assign clear roles (facilitator, note-taker, timekeeper).
2. Circulate agendas and materials in advance.
3. Use simple language and encourage participation across all constituencies.
4. Maintain inclusivity and sensitivity to connectivity constraints.
5. Summarize and debrief at the end of each session.
6. Record sessions (with consent) and capture screenshots for documentation.
7. Adapt facilitation style based on technology used and group familiarity.

**Annex II: Use of Virtual Applications**

| **Platform** | **Functionality** | **Key Tips** |
| --- | --- | --- |
| **Zoom** | Video/Audio conferencing; screen sharing | Free up to 40 minutes; can record (with consent). |
| **Skype** | Voice/video calls; screen share | Free; recordable; can host multiple participants. |
| **WhatsApp** | Voice/video call; group text | Max 4 participants on call; can share media. |
| **Viber** | Voice/video call; group text | Up to 5 participants; record using external apps. |
| **Email** | Formal communication | Use for asynchronous input and documentation. |
| **SMS/Phone** | Direct feedback from participants with limited internet | Capture text logs/screenshots for evidence. |

All applications should be used per participant comfort and accessibility.

**Annex III: Evidence Requirements**

Each consultation (virtual or hybrid) must maintain verifiable records:

- Invitations and meeting links.
- Screenshots/photos of discussions.
- Signed or digital attendance sheets.
- Summary reports.
- Financial utilization statements (if applicable).

1. **Conclusion**

This guidance aims to institutionalize a structured, inclusive, and flexible virtual/hybrid approach to the Country Dialogue Process for developing and revising NSPs and preparing the Global Fund Grant Cycle 6 funding requests.

By adhering to these steps, CCM Nepal and its stakeholders ensure:

- **Meaningful participation** of all constituencies.
- **Transparency and accountability** in decision-making.
- **Alignment** with national strategies and Global Fund principles of country ownership.
