# Supplementary material for: Community and stakeholder engagement in national priority setting and participatory research for HIV, Tuberculosis, and Malaria programs in Nepal
Source: Res Involv Engagem. 2026 May 22;12:69. doi: 10.1186/s40900-026-00907-3 (PMC13198034; doi:10.1186/s40900-026-00907-3)
Supplement: Supplementary file 7 — Supplementary material 7 [file 40900_2026_907_MOESM7_ESM.docx]

**Supplementary File 3**

**Qualitative Analysis Frameworks, Matrices, and Tables**

## Purpose and scope

This supplementary file documents how qualitative evidence from Nepal’s nationwide consultations for HIV, TB, and malaria was transformed into actionable strategy and financing decisions. Audio from community discussions, consultation meetings, and key informant interviews was transcribed verbatim, translated where needed, and organized with metadata. Thirty rich and representative transcripts were coded in NVivo 12 using a hybrid inductive plus deductive approach, while the remaining transcripts were reviewed to elaborate and stress test findings. Summary matrices and framework charts were developed to help identify recurring patterns across the data and to compare signals across provinces, stakeholder levels, and population groups.

Six cross cutting themes consistently emerged: access and equity, stigma and discrimination, community engagement, health system gaps, integration and coordination, and sustainability and ownership. These synthesized findings were brought into participatory prioritization sessions, disease specific validation meetings, and finally endorsed by the CCM, ensuring that National Strategic Plans and the aligned Global Fund and USAID PEPFAR packages reflected community voice, subnational feasibility, and system realities. Reliability safeguards, ethical protections, and decision traceability crosswalks strengthened credibility and transparency. The included framework chart and matrices serve as reproducible tools that link qualitative insights to NSP chapters, partner roles, budgets, and monitoring indicators.

**Key note**: **Summary matrices and framework charts were developed to help identify recurring patterns across the data.**

## End to end analytic pipeline framework

## **Table B1. Analytic pipeline from raw data to policy and financing decisions**

| **Stage** | **Inputs** | **Methods and tools** | **Primary outputs** | **How outputs were used** |
| --- | --- | --- | --- | --- |
| 1. Ingestion | Audio from community discussions, key informant interviews, multi stakeholder meetings; field notes; agendas | Verbatim transcription, bilingual translation checks, metadata tagging | Clean transcript pack with IDs by province, population, and method | Forms the analyzable corpus and preserves traceability |
| 2. Selection | Entire corpus across 7 provinces and 72 municipalities | Maximum variation sampling for a depth sample of 30 transcripts | Thirty information rich transcripts for NVivo 12 coding; remaining transcripts flagged for synthesis | Balances depth and breadth; prevents selection bias |
| 3. Coding | Clean transcripts with metadata | Hybrid coding in NVivo 12 using inductive plus deductive nodes; double coding on 20 percent of the depth sample | Stable codebook, coded dataset, intercoder log | Creates a reliable thematic index of stakeholder signals |
| 4. Frame-working | Coded segments by theme, location, and stakeholder | Framework method; summary matrices; framework charts | Cross case matrices and charts by theme, geography, and subgroup | Surfaces recurring patterns and contrasts across the dataset |
| 5. Synthesis | Matrix summaries, quotes, pattern notes | Thematic memos; convergence and divergence testing | Thematic findings aligned to NSP objectives and indicators | Turns qualitative signals into actionable strategy text |
| 6. Decision | Prioritization decks, draft lists of interventions | Participatory ranking, validation meetings, CCM endorsement | Ranked priorities, budgets, partner roles, PAAR lists | Locks decisions and aligns Global Fund and PEPFAR with NSPs |

## Data management, transcription, and selection

- **Data sources:** Community Group Discussions, community consultation meetings, and key informant interviews across all seven provinces and 72 municipalities.
- **Transcription and translation:** Audio recorded sessions were transcribed verbatim. When conducted in Nepali or other local languages, transcripts were translated into English by bilingual researchers.
- **NVivo 12 set:** Thirty transcripts judged rich and representative by level and stakeholder group were imported into NVivo 12 for thematic coding. Remaining transcripts were reviewed in full to elaborate and stress test the coded results.

## Codebook and node architecture

## **Table D1. Master codebook with definitions, inclusion or exclusion rules, and example indicators**

| **Code domain** | **Operational definition** | **Include when** | **Exclude when** | **Example indicator or signal** | **Typical NVivo node label** |
| --- | --- | --- | --- | --- | --- |
| Access and equity | Barriers to access or continuity due to geography, cost, hours, language, disability, mobility | Mentions of travel time, transport cost, user fees, clinic hours, migrant contexts | Purely clinical detail without barrier context | Percent facilities with after-hours services in target wards | 01_Access_Equity |
| Stigma and discrimination | Stigmatizing or discriminatory practices deterring testing or treatment | Descriptions of shaming, refusal of service, confidentiality breaches | General dissatisfaction not tied to stigma or rights | Providers completing stigma reduction training | 02_Stigma_Rights_Gender |
| Community engagement | Roles of FCHVs, peer navigators, mothers groups, PLHIV and TB survivor networks | Community led outreach, CLM, peer support for adherence | One off IEC events without engagement structure | Wards with active peer navigators | 03_Community_Engagement |
| Health system gaps | Staff, supply chain, laboratory, data, and supervision bottlenecks | Stock outs, turnover, lab capacity issues, data timeliness problems | Individual clinical outcomes unlinked to system gaps | Months without stock out of RDTs or anti TB drugs | 04_System_Gaps_RSSH |
| Integration and coordination | Cross program linkages and referrals, PPM, prison and migration coordination | TB HIV co testing, sputum transport, cross border referral, prison intake | Internal clinic workflow unrelated to linkages | TB patients offered HIV test at DOTS sites | 05_Integration_Coordination |
| Sustainability and ownership | Domestic co financing, local budgeting, institutionalizing community systems | Palika budgets for outreach, government shares for commodities | Short term pilots without transition plan | Local budgets for CLT and ACF | 06_Sustainability_Ownership |

## **Table D2. Node hierarchy snapshot**

| **Parent node** | **Selected child nodes** |
| --- | --- |
| 01_Access_Equity | Distance_Transport, Cost_UserFees, Clinic_Hours, Mobile_Populations, Language_Disability |
| 02_Stigma_Rights_Gender | Facility_Stigma, Community_Stigma, KP_Discrimination, Privacy_Confidentiality, Gender_Barriers |
| 03_Community_Engagement | FCHV_Roles, Peer_Navigation, Mothers_Groups, CLM, Youth_Engagement |
| 04_System_Gaps_RSSH | Stockouts_PSM, Lab_Capacity, HRH_Turnover, Supervision, Data_Quality |
| 05_Integration_Coordination | TBHIV_Collab, PPM_Private, Prison_Services, Migration_CrossBorder, Referral_Pathways |
| 06_Sustainability_Ownership | GoN_CoFinance, Palika_Budgeting, Transition_Plans, Costing_Roadmaps |
|  |  |

## Reliability and rigor

## **Table E1. Inter-coder reliability and quality safeguards**

| **Step** | **Target** | **Evidence of completion** | **Action if below target** |
| --- | --- | --- | --- |
| Double coding | 20 percent of the depth sample | Parallel coding on 6 transcripts with reconciliation notes | Expand double coded set and retrain analysts |
| Agreement threshold | Stable definitions and qualitative convergence | No rule changes after the third reconciliation round | Freeze codebook; document residual ambiguities |
| Translation fidelity | Spot check 10 percent of translations | Reviewer approvals logged; corrections annotated | Back translation on a sample; glossary update |
| Data protection | Zero breaches | Access logs for audio and transcripts | Immediate lock down and notification protocol |

## Thematic findings that informed NSPs and aligned financing

1. **Access and equity**: distance, cost, hours, and mobility barriers for remote communities, migrants, urban poor, and marginalized groups.
2. **Stigma and discrimination**: facility and social stigma reduce testing and adherence, particularly for PLHIV, TB survivors, FSWs, MSM, TG, PWID, prisoners, and migrants.
3. **Community engagement**: FCHVs, peer navigators, mother’s groups, and local NGOs extend reach and improve retention; require structured roles and resourcing.
4. **Health system gaps**: recurring stock-outs, staff constraints, laboratory capacity challenges, and fragmented data flows.
5. **Integration and coordination**: TB HIV collaboration, PPM, prison intake screening, and cross border referrals.
6. **Sustainability and ownership**: domestic co financing trajectories and local budgeting, institutionalized community systems, and partner alignment to the NSPs.

## Prioritization, validation, and CCM endorsement

## Following NVivo coding and framework synthesis, outputs were taken into structured decision forums:

- **Prioritization:** HIV on 2 August 2020; TB on 9 August 2020; malaria in early August 2020.
- **Validation:** TB on 19 August 2020; malaria on 19 August 2020; HIV on 20 August 2020.
- **CCM endorsement:** 113th CCM Nepal meeting on 24 August 2020.

Key HIV refinements included clarification of migrant and prison coverage, stronger attention to women and young key populations, integration of mental health screening in community led testing, solutions for viral load constraints through GeneXpert and PCR platforms, and PMTCT data quality actions. TB validation confirmed expansion of ACF and micro planning including migrants and prisons, scaling GeneXpert, AI assisted CXR, e HMIS improvements, and clearer survivor and private sector roles. Malaria validation confirmed the community testing expansion pathway through VMW and FCHV, domestic shares for LLINs, RDTs, and microscopy, HR and lab strengthening, and stronger linkages with academia and private sector.

## Master framework chart aggregating HIV, TB, and malaria insights

**Here, c**olumns are the six master themes. Each cell contains a concise analytic summary and, where relevant, a short anonymized quote or a decision captured during national events.

**Table H1. Framework chart**

| **Transcript or subgroup** | **Access and equity** | **Stigma and discrimination** | **Community engagement** | **Health system gaps** | **Integration and coordination** | **Sustainability** |
| --- | --- | --- | --- | --- | --- | --- |
| FGD, remote hill municipality | Travel 4 to 8 hours; high transport cost | Neighborhood TB stigma deters testing | FCHVs identify chronic cough and escort | Stock outs of RDTs and anti TB drugs | Referral to district hospital inconsistent | Palika ready to co-finance weekend outreach |
| KII, provincial TB officer | Urban poor and mobile workers underdiagnosed | MDR TB stigma affects adherence and tracing | Peer supporters effective for follow up | Turnover and limited microscopy and GX | Interest in TB HIV co located screening | Budget line needed for sputum transport |
| FGD, key population network, border district | Migrants miss testing at departure and return | Sex work related stigma limits clinic attendance | Peer navigators lift uptake at night | Bed net replacement gaps at worksites | Weak fever to confirmatory referral | Municipality offers logistics for outreach |
| KII, private pharmacy owner | First contact for cough or fever is pharmacy | HIV disclosure fears deter referrals | Discreet referral corners feasible | No RDT supply for private outlets | PPM could link pharmacies to DOTS and RDTs | Sustainable with small incentives and tools |
| FGD, prison health staff | Intake and transfer delays for diagnosis | Stigma among inmates and guards | Peer educators workable inside facilities | Drug continuity breaks on transfer | TB HIV integrated screening at intake | Needs MoHA and MoHP joint SOP and budget |
| Youth virtual forum | Youth friendly hours lacking | Bullying and discrimination deter service use | Digital outreach and peer linkage | Test kit stock outs seen in lockdown | Link prevention with SRH and mental health | Annual budget for digital engagement |
| Malaria community meeting, Koshi Province | Remote hamlets beyond 2 hour walk | Fear of blame for imported malaria | Mothers groups schedule LLIN reminders | RDT stock outs in monsoon months | Cross border referral gaps for febrile migrants | District to co fund VMW travel costs |
| National civil society leader | Local feedback reaches decision tables | Facility stigma openly flagged | Community dialogues now standard | Community stock out alerts escalated | Shared lab and logistics platforms | Public financing commitments published |
| Policymaker, planning commission | Equity lens for scarce resources | Anti-stigma messaging to be mainstreamed | Constituency consultations to remain annual | Need routine DQA for data quality | Align TB HIV malaria with PSM and HMIS | Domestic co financing and phased transition |
| HIV validation meeting, 20 Aug | Clarify migrant and prison coverage | Focus on women and young KPs | Integrate mental health into CLT | Use GX and PCR for VL constraints | Coordinate TB HIV in prisons and monitoring | Funding package confirmed as NSP instrument |
| Malaria validation meeting, 19 Aug | Sustain and expand VMW and FCHV testing | Messaging to reduce blame on imported cases | Increase community engagement indicators | Reinforce labs and POE data systems | Engage private sector and academia | Government shares defined for LLINs, RDTs, microscopy |
| TB validation meeting, 19 Aug | Expand ACF, micro planning, migrants and prisons | Engage survivor groups and remove barriers | Clarify CSO roles; mobilize ORWs and FCHVs | Scale GX, AI CXR, and iHMIS updates | Define PPM and strengthen TB HIV collaboration | Co-financing and PAAR confirm GF as NSP supplement |

## Summary matrices

### I1. Cross level summary matrix

**By stakeholder level and theme emphasis**

| **Level or group** | **Access and equity** | **Stigma and discrimination** | **Community engagement** | **Health system gaps** | **Integration and coordination** | **Sustainability** |
| --- | --- | --- | --- | --- | --- | --- |
| Community and key populations | Distance, cost, hours limit reach; migrants missed | Facility and social stigma in PLHIV, TB, FSW, MSM, TG | FCHV and peer models preferred; need resources | Stock-outs and referral breaks visible at community level | Desire for one stop screening and clear referral slips | Palikas can co finance when roles and tools are defined |
| Providers and managers | Peripheral staff thin; lab access uneven | Disclosure fears complicate adherence support | Want structured peer programs with supervision | Need PSM tools, DQA, mentorship, and GX maintenance | TB HIV bidirectional testing and sputum transport | Budget clarity and transition trajectories requested |
| Provincial and local officials | Remote wards and slums underserved | Need provider stigma reduction packages | Endorse CLM with joint reviews | HR and procurement delays constrain plans | Cross border and prison coordination needed | Annual local health budget earmarks feasible |
| National programs and partners | Equity targeting needed for scarce funds | Rights and gender actions mainstreamed | CLM to inform routine reviews | RSSH for labs, HR, data, and supply chains | PPM and joint monitoring mechanisms | Domestic co financing and phased cost shifts agreed |

### I2. Provincial bottleneck matrix

**Table I2. By province or region**

| **Province or region** | **Geographic access** | **Commodities** | **Lab capacity** | **Human resources** | **Referral and data** |
| --- | --- | --- | --- | --- | --- |
| Koshi province border belt | \| Remote hamlets beyond 2 hours; highly mobile migrants; after-hours demand near points of entry \| \| --- \| | \| Remote hamlets beyond 2 hours; highly mobile migrants; after-hours demand near points of entry \| \| --- \| | \| Remote hamlets beyond 2 hours; highly mobile migrants; after-hours demand near points of entry \| \| --- \| | \| Remote hamlets beyond 2 hours; highly mobile migrants; after-hours demand near points of entry \| \| --- \| | \| Remote hamlets beyond 2 hours; highly mobile migrants; after-hours demand near points of entry \| \| --- \| |
| Madhesh border belt | \| Dense night mobility; KP prefer after-hours access; urban slum pockets underserved \| \| --- \| | \| Dense night mobility; KP prefer after-hours access; urban slum pockets underserved \| \| --- \| | \| Dense night mobility; KP prefer after-hours access; urban slum pockets underserved \| \| --- \| | \| Dense night mobility; KP prefer after-hours access; urban slum pockets underserved \| \| --- \| | \| Dense night mobility; KP prefer after-hours access; urban slum pockets underserved \| \| --- \| |
| Bagmati urban poor pockets | \| Travel cost and lost wages; clinic hours conflict with shift work \| \| --- \| | \| Travel cost and lost wages; clinic hours conflict with shift work \| \| --- \| | \| Travel cost and lost wages; clinic hours conflict with shift work \| \| --- \| | \| Travel cost and lost wages; clinic hours conflict with shift work \| \| --- \| | \| Travel cost and lost wages; clinic hours conflict with shift work \| \| --- \| |
| Gandaki hills | \| Long travel and transport cost; seasonal road closures; elderly and disability access gaps \| \| --- \| | \| Long travel and transport cost; seasonal road closures; elderly and disability access gaps \| \| --- \| | \| Long travel and transport cost; seasonal road closures; elderly and disability access gaps \| \| --- \| | \| Long travel and transport cost; seasonal road closures; elderly and disability access gaps \| \| --- \| | \| Long travel and transport cost; seasonal road closures; elderly and disability access gaps \| \| --- \| |
| Lumbini plains | \| Seasonal migrant peaks at kilns and farms not routinely reached \| \| --- \| | \| Seasonal migrant peaks at kilns and farms not routinely reached \| \| --- \| | \| Seasonal migrant peaks at kilns and farms not routinely reached \| \| --- \| | \| Seasonal migrant peaks at kilns and farms not routinely reached \| \| --- \| | \| Seasonal migrant peaks at kilns and farms not routinely reached \| \| --- \| |
| Karnali mountains | \| Very remote access; weather isolation; catastrophic transport cost \| \| --- \| | \| Very remote access; weather isolation; catastrophic transport cost \| \| --- \| | \| Very remote access; weather isolation; catastrophic transport cost \| \| --- \| | \| Very remote access; weather isolation; catastrophic transport cost \| \| --- \| | \| Very remote access; weather isolation; catastrophic transport cost \| \| --- \| |
| Sudurpashchim far west | \| Cross-border seasonality; forest goers and fishers miss routine services \| \| --- \| | \| Cross-border seasonality; forest goers and fishers miss routine services \| \| --- \| | \| Cross-border seasonality; forest goers and fishers miss routine services \| \| --- \| | \| Cross-border seasonality; forest goers and fishers miss routine services \| \| --- \| | \| Cross-border seasonality; forest goers and fishers miss routine services \| \| --- \| |

### I3. Population pathway matrix

**Table I3. By population group and service pathway**

| **Population group** | **Entry point** | **Key barriers** | **Engagement model** | **Integration need** | **Sustainability lever** |
| --- | --- | --- | --- | --- | --- |
| PLHIV | OPD, KP clinics, peers | Stigma, privacy concerns, clinic hours | Peer navigation, community led testing | VL via GX and PCR, GBV referral | Palika budget for peers and CLM |
| TB survivors and presumptive TB | DOTS, pharmacies, FCHVs | Travel cost, lost wages, MDR stigma | FCHV cough screening and sputum transport | Bidirectional TB HIV testing | Provincial budget for sputum courier and oxygen |
| FSW | Night outreach, community clinics | Discrimination, safety, policing | Peer led night outreach, safe space clinics | Link to SRH, HIV, legal aid | PEPFAR GF division of labor, local MOUs |
| MSM and TG | KP clinics, peers | Stigma, documentation issues | Peer linkage, youth friendly hours | Mental health linkage within HIV | Budget for youth and TG inclusive services |
| PWID | OST sites, peers | Criminalization fears | OST plus CLM and outreach | HIV HCV services integrated | Transition OST costs to domestic shares |
| Migrants and spouses | POE desks, return villages | Missed testing windows | CLT at border and villages | Cross border referral SOP | Local budget for travel vouchers |
| Prison inmates | Intake checks | Transfer breaks, stigma | Peer educators in prisons | TB HIV integrated screening | MoHA MoHP joint SOP and budget lines |
| Malaria vulnerable groups | FCHVs, VMWs, farms | Distance, net replacement | VMW plus mothers groups | Private sector for RDTs and fever desks | Government shares for LLINs and RDTs year by year |

## Decision traceability crosswalk

**Table J1. From theme to NSP chapter and aligned financing**

| **Theme signal from analysis** | **NSP priority or action** | **Global Fund module or activity** | **USAID PEPFAR complementary focus** | **Lead implementers and partners** | **Monitoring indicator** |
| --- | --- | --- | --- | --- | --- |
| Long travel and cost block access in remote wards | Mobile and weekend outreach for HIV, TB, malaria | Community systems and outreach | Peer navigation and differentiated service delivery | Palikas, national programs, CSOs | Outreach sessions in remote wards |
| Stigma and privacy concerns in facilities | Provider stigma reduction and confidentiality SOPs | Removing human rights and gender barriers | KP friendly service packages | NCASC, NTC, EDCD, CSOs | Providers trained and observed compliance |
| Weak TB HIV linkages | Bidirectional testing and integrated registers | TB HIV collaboration | Integrated adherence support | NTC, NCASC, facilities | Percent of TB patients tested for HIV |
| Private sector as first contact | PPM model for TB and fever testing | Engage all care providers | Private reporting and linkage | NTC, EDCD, pharmacy associations | Private notifications captured |
| Prison and detention gaps | Intake screening and continuity protocols | TB HIV prisons package | Peer educators and referral pilots | MoHA, DoHS, prison health | New entrants screened within 48 hours |
| Sustainability and domestic shares | Year by year co financing and transition | Co-financing schedules and annexes | Complement with PEPFAR where strategic | MoF, MoHP, partners | Percent of costs covered by government |

## Illustrative NVivo queries used to build matrices

**Table K1. NVivo query types and how they fed the matrices**

| **Query type** | **Purpose** | **Example output used in matrices** |
| --- | --- | --- |
| Matrix coding query | Compare 6 themes by 8 stakeholder types | Heat map to populate I1 cross level matrix |
| Crosstab by province | Identify where stock-outs and lab delays co occur | Province specific entries in I2 |
| Text search cluster | Find language around privacy and stigma | Quote bank for framework chart H1 |
| Case classification summary | Pull metrics by population group | Inputs to I3 pathway rows |

## Governance and ethics framework

**Table L1. Consent, confidentiality, and data security**

| **Domain** | **Practice** | **Evidence** |
| --- | --- | --- |
| Consent | Verbal or written consent recorded for each session | Signed sheets or audio consent noted in transcript header |
| Confidentiality | Anonymize quotes, generalize identifiers | Quotes tagged only by type, level, and subgroup |
| Data security | Role based access, version control | Access logs and file hashes maintained |
| Member checking | Validate interpretations in validation meetings | Meeting notes with changes accepted and recorded |

## Final narrative alignment

The frameworks and tables above show how qualitative inputs were translated into NSP ready content and then expressed through aligned financing under the Global Fund and USAID PEPFAR. The framework chart H1 and summary matrices I1 to I3 were the backbone of synthesis and decision, ensuring that what communities said is visible in what the country plans to do, how it will be funded, and how success will be monitored.

## Annex 1. Expanded framework chart excerpt for direct copy into a Word appendix

| **Transcript or subgroup** | **Access and equity** | **Stigma and discrimination** | **Community engagement** | **Health system gaps** | **Integration and coordination** | **Sustainability** |
| --- | --- | --- | --- | --- | --- | --- |
| Factory workers FGD, Lumbini | Shift work prevents clinic visits | TB stigma among coworkers | Peer supporters available at work sites | No sputum transport from factory clinic | Referral slips not recognized at district lab | Employer ready to co- fund screening days |
| Elderly FGD, Gandaki | Limited mobility and caregiver time | Fear of blame for cough | FCHVs can schedule home screening | No home based sample collection | Link with NCD and TB screening in OPD | Palika to budget home visits quarterly |
| Refugee camp KII, Province 1 | Permit restrictions reduce access | Stigma around HIV testing | Peer educators inside camp effective | Periodic stock-outs of HIV test kits | Need camp to facility referral MOUs | NGO partner to co finance transport |
| Mothers group, malaria village | Distance to IRS teams | Blame after imported cases | Group can coordinate LLIN use | IRS timing misses harvest season | Share LLIN replacement schedule with ward | Palika to allocate for community mobilizers |
| Pharmacist, Bagmati slum | First stop for fever and cough | HIV stigma deters clinic referral | Private referral card feasible | No RDTs for private sector | PPM enrollment requested | Small incentive to report and refer |
| DOTS nurse, Karnali | Patients walk long distances | MDR stigma impairs DOTS | Volunteers can take DOTS near homes | Oxygen not available during exacerbations | TB HIV joint clinic day proposed | Province to fund oxygen concentrators |
| Youth MSM TG group | Hours and ID issues block access | Bullying and discrimination | Digital outreach and peer linkage | Test kits stocked out during lockdown | SRH and mental health integration | Annual budget for youth engagement |
| Prison inmate peer group | Intake checks uneven | Peer stigma deters disclosure | Peer educator system piloted | Drug continuity breaks on transfer | Integrate TB HIV at intake with SOP | Budget lines under MoHA and MoHP |
| Returnee migrants FGD | Missed testing windows at POE | Fear of losing job on diagnosis | CLT at entry and return villages | Data not shared across districts | Cross border referral desk proposed | Travel vouchers via local budget |
| National PLHIV women network | Clinic hour rigidity hurts retention | Double stigma with GBV | Community mentors improve retention | Viral load access outside hubs limited | GBV referral integrated with HIV care | Build CLM dashboards with simple tools |
| CSO leader national | Local feedback informs national plans | Stigma flagged in service points | Dialogues adopted as routine practice | Supply chain alerts from communities | Shared logistics platforms across programs | Public financing commitments published |
| Provincial malaria officer | Remote hot spots persist | Social blame after reintroduction | VMWs plus mothers groups work | RDTs thin in peak months | Coordinate with private clinics | Government shares for LLINs and RDTs increase yearly |
| TB survivor group | Travel and lost wages deter follow up | Stigma in family and workplace | Survivor mentors improve adherence | GX hub far, sample delays | Link DOTS to sputum courier | Social protection reduces catastrophic cost |
| KP coalition | Irregular hours limit uptake | Facility discrimination persists | Peer led micro plans effective | Provider sensitivity training needed | Clarify partner division by KP | Local budgets for peer stipends |
| Municipal leader, Gandaki | Budget constraints but strong intent | Public messaging to reduce stigma | Ward committees include PLHIV and survivors | Procurement delays slow response | Align with national and donor investments | Council resolution to earmark health funds |
| Technical partner team | Coverage mapping reduces gaps | Rights based approaches standard | CLM structures proposed nationwide | Commodity pipeline visibility weak | Resources reprioritized jointly | Joint financing roadmaps reduce duplication |

## Annex 2. Copy ready matrix templates

### T1. Province matrix (populated example)

| **Province or region** | **Access gaps** | **Commodity status** | **Lab status** | **HR status** | **Referral data status** | **Actions agreed** |
| --- | --- | --- | --- | --- | --- | --- |
| Koshi province | Remote hamlets beyond 2 hours; highly mobile migrants; after-hours demand near points of entry | Monsoon RDT gaps; LLIN replacement delays in riverine wards; TB drug buffer thin in 2 municipalities | Microscopy functional; GeneXpert limited to hubs; sample transport irregular | Early turnover of peripheral staff; limited refresher training | POE tallies not linked to eHMIS; cross-border referral logs non-standard | Pre-position RDTs and LLINs pre-monsoon; contract courier for sputum and VL; standard POE referral form; monthly corridor review; expand GeneXpert catchment (HIV VL and TB) in line with NSP lab directions. |
| Madhesh border belt | Dense night mobility; KP prefer after-hours access; urban slum pockets underserved | HIV test kits adequate; ART refill continuity gaps during migration; stock alerts slow | GeneXpert uptime affected by maintenance; VL shipment to PCR hubs irregular | Peer navigators active but unfunded; counselor shortage for KP-friendly services | No SOPs for prison and migrant referral; TB–HIV bidirectional register incomplete | Fund peer navigators and weekend clinics; formalize ART refill at border clinics; sign prison and migrant SOPs; vendor maintenance for GeneXpert; SMS stock alerts to province store per NSP PSM guidance. |
| Bagmati urban poor pockets | Travel cost and lost wages; clinic hours conflict with shift work | TB drug stock alerts irregular; HIV commodities adequate; malaria commodities needed for fever triage | Labs close but long queues; GeneXpert capacity good with scheduling issues | Staff stretched at high-volume sites; supervision gaps | PPM and private notifications weak; pharmacy referrals not captured | Extend hours and Saturday clinics; set buffer stock triggers; fast-track lab triage; enroll pharmacies and private clinics in PPM with simple reporting app aligned to TB NSP roles for private secto |
| Gandaki hills | Long travel and transport cost; seasonal road closures; elderly and disability access gaps | Seasonal LLIN stockouts in remote wards; winter RDT gaps | Microscopy adequate; GeneXpert at provincial hospital far for mountain municipalities | FCHV workload high; limited outreach allowances | Referral slips inconsistent across municipalities and district hospital | Quarterly mobile integrated camps; align LLIN delivery with dry-season roads; FCHV micro-grants; standardized referral slips; tele-consult triage for presumptive TB in line with NSP community systems and program management. |
| Lumbini plains | Seasonal migrant peaks at kilns and farms not routinely reached | RDTs usually adequate; occasional HIV kit redistribution; TB buffers moderate | Labs fair; QA and proficiency testing need strengthening | Volunteers active but untrained in ACF micro-planning | Inter-district referral not standardized; paper trail lost when migrants move | Run migrant screening bursts at worksites; quarterly lab QA/PT; train volunteers in ACF micro-plans; inter-district referral SOP with simple digital handover per NSP surveillance and M&E. |
| Karnali mountains | Very remote access; weather isolation; catastrophic transport cost | Winter stockouts of TB drugs and RDTs due to road closure; no LLIN buffer | Lab coverage patchy; GeneXpert absent in several districts; courier ad hoc | Few skilled staff; rotation gaps; limited biomedical support | Data delays from remote wards; monthly aggregation only | Combined outreach treks for TB–HIV–malaria; pre-position winter stocks via airlift; fixed courier days; CXR and sputum transport scale-up; SMS rapid reporting from remote posts, consistent with subnational roles in TB NSP and surveillance in Malaria NSP. |
| Sudurpashchim far west | Cross-border seasonality; forest goers and fishers miss routine services | LLIN replacement uneven; malaria RDT gaps in peak months; TB commodities stable | Labs OK in hubs; microscopy coverage fair; EQA feedback slow | Trained staff present but supportive supervision infrequent | Cross-border data sharing ad hoc; no shared dashboard with adjacent districts in India | Synchronize LLIN replacement with migration calendar; surge supervision; cross-border data-sharing note; monthly line-list exchange, aligned to check posts and cross-border collaboration in Malaria NSP. |
| National cross-border corridor cluster | Multiple POE points with inconsistent screening and referral; language barriers | POE kits available but replenishment slow; IEC not localized | On-site testing limited; district labs handle confirmation | POE staffing rotates without formal handover | POE data not mapped to facility line lists; duplicates in counts | Corridor micro-plans; bilingual IEC; POE to facility QR referral handover; weekly reconciliation between POE and facility lists per case-based surveillance objective |
| Refugee and special settlements | Movement restrictions; documentation barriers; limited KP-friendly access | HIV test kit shortages periodic; malaria commodities adequate; TB drug continuity at transfer risk | Camp clinics functional; confirmatory capacity limited | Peer educators present; refresher training intermittent | Referral MOUs partial; feedback to camp clinics slow | Renew two-way MOUs; designate district focal person; scheduled medication handover on transfers; finance peer refresher trainings in line with community systems and program management chapters. |

### Template T2. Population pathway (populated example)

| **Population** | **Entry point** | **Barriers** | **Engagement model** | **Integration needs** | **Financing alignment** | **KPIs** |
| --- | --- | --- | --- | --- | --- | --- |
| People living with HIV | OPD, KP clinics, peer networks | Stigma and privacy; rigid clinic hours; transport cost | Peer navigation; community-led testing; multi-month dispensing and flexible pickup | Viral load via GeneXpert and PCR hubs; GBV screening and referral; TB screening every visit | GF supports CLT and lab networking; PEPFAR supports KP and peers; GoN co-finances commodities | VL coverage and suppression; 12-month retention; TB screening at each visit in line with NHSP indicators. |
| Presumptive TB and TB survivors | DOTS, pharmacies, FCHVs | Lost wages and travel cost; MDR stigma; delayed diagnosis | FCHV cough screening; sputum collection and transport; survivor mentors | Bidirectional TB–HIV testing; oxygen at district level; social protection linkage | GF supports ACF, courier, GeneXpert; GoN funds oxygen and DOTS; partners support survivor groups | Time to diagnosis; HIV test among TB patients; treatment success; catastrophic cost index per TB NSP. |
| Female sex workers | Night outreach, drop-in centers, KP clinics | Discrimination and safety; policing concerns | Peer-led night outreach; safe-space clinics; discreet testing and linkage | SRH and HIV integration; legal aid referral; violence mitigation protocols | PEPFAR leads KP packages; GF complements outreach and CLM; municipalities sign MOUs | FSW reached; testing uptake; linkage to prevention or ART; violence cases addressed per CRG priorities. |
| MSM and TG | KP clinics, peers, social venues | Stigma and ID issues; youth barriers | Peer linkage; youth friendly hours; digital outreach | Mental health inside HIV services; hormone linkage for TG | PEPFAR supports KP and youth; GF funds stigma reduction and CLM; GoN prepares facility readiness | MSM TG testing coverage; time to ART start; satisfaction with KP-friendly services aligned to NHSP |
| People who inject drugs | OST sites, harm reduction | Criminalization fears; instability; overdose risk | OST plus outreach; peer led CLM | Integrated HIV and HCV testing and care; overdose prevention; TB screening in OST | PEPFAR and GF coordinate OST and harm reduction; GoN transitions OST costs | OST coverage; HIV HCV testing uptake; needle-syringe distribution; OST retention within NHSP framework. |
| Migrants and spouses | POE desks, return villages, transport hubs | Missed testing windows; cost; frequent moves | Community-led testing at border and return villages; travel vouchers | Cross-border referral with feedback loop; malaria fever desks; TB continuity | GF funds CLT and POE systems; GoN funds vouchers; partners support cross-border linkage | Percent screened; successful cross-border referrals; treatment initiation within 7 days, in line with Malaria NSP and TB NSP mobility focus. |
| Prison inmates | Intake checks; prison clinic | Transfer interruptions; stigma; poor privacy | Peer educators in prisons; routine intake and periodic mass screening | TB–HIV integrated screening; ART and DOT continuity; MDR isolation protocols | GF supports prison package; GoN and MoHA fund SOPs and continuity; partners train peers | New entrants screened within 48 hours; continuity on transfer; ART interruptions per 100 inmates per NSP governance roles. |
| Adolescent girls and young women | Schools, youth clinics, community groups | Limited youth-friendly hours; fear of disclosure | Youth peer educators; school-based SRH and HIV awareness; hotline and chat | Link HIV prevention with SRH, GBV support, mental health | PEPFAR supports youth where applicable; GF supports IEC and CLM; GoN funds youth corners | Number reached with comprehensive package; testing uptake; GBV referrals completed per NHSP. |
| Elderly and geriatric populations | OPD, home visits via FCHVs | Mobility limits; comorbidities; low TB suspicion | Home-based screening and sputum pickup; caregiver engagement | TB screening integrated with NCD visits; palliative support linkage | GF supports community screening; GoN funds home-visit allowances; partners provide assistive aids | Elderly screened for TB annually; treatment initiation time; follow-up completion consistent with TB NSP. |
| Marginalized caste or ethnic groups including Mushar | Community meetings, FCHVs | Extreme poverty; social exclusion; low health literacy | Peer mobilizers from same community; targeted outreach and incentives | Combined TB–HIV–malaria screening; nutrition and social protection link | GF supports outreach; GoN funds social protection linkages; CSOs implement | Outreach coverage; testing uptake; initiation and completion rates within equity focus of NSPs. |
| Refugees and camp residents | Camp clinics, UN partner referrals | Documentation and movement restrictions; language barriers | Peer educators; bilingual IEC; escorted referral to public facilities | HIV, TB, malaria referral MOUs; feedback loop to camp clinic | Partners support camp services; GF complements testing; GoN provides confirmatory care | Number referred and received; turnaround for confirmation; treatment start rate aligned to program management chapters. |
| Forest goers and agricultural workers | Worksite outreach, FCHVs, village malaria workers | Distance; seasonality; low net use at peak work | Fever desks at farms; net-use reminders; mobile testing | Private clinics and pharmacies linked for RDT and TB triage | GF funds VMWs and RDTs; GoN funds LLINs and microscopy; private sector collaborates | Fever cases tested within 24 hours; LLIN use rate; positivity in hot spots per Malaria NSP objectives. |
| Private sector first-contact clients | Pharmacies, private GP clinics | Cost; confidentiality; weak referral pathways | PPM enrollment; discreet referral cards; simple app reporting | TB–HIV co-testing pathways; malaria RDT availability in accredited outlets | GF funds PPM systems; GoN accredits outlets; associations co-implement | Private notifications captured; referrals completed; time from first contact to diagnosis per TB NSP PPM. |
