# Supplementary material for: Community and stakeholder engagement in national priority setting and participatory research for HIV, Tuberculosis, and Malaria programs in Nepal
Source: Res Involv Engagem. 2026 May 22;12:69. doi: 10.1186/s40900-026-00907-3 (PMC13198034; doi:10.1186/s40900-026-00907-3)
Supplement: Supplementary file 8 — Supplementary material 8 [file 40900_2026_907_MOESM8_ESM.docx]

**Supplementary File 4**

**Hybrid Coding Framework and NSP-Aligned Thematic Analysis Methods**

**Purpose and positioning**

This supplementary document details the analytic approach used to translate qualitative inputs from national consultations into strategic, NSP-aligned themes. The primary objective was to support the development and revision of Nepal’s National Strategic Plans for HIV, TB, and Malaria, while ensuring that Global Fund and USAID/PEPFAR priorities complemented, rather than substituted, the NSPs. The HIV NSP emphasizes broad consultation and systems strengthening, including resilient and sustainable systems for health and community systems strengthening within a decentralized program architecture, which provided the core policy frame for our analysis. The TB NSP was itself informed by extensive country dialogue, with clearly delineated roles for federal, provincial, and local governments, which we mirrored in our coding structure. The Malaria NSP’s five strategic objective areas (surveillance, transmission reduction, diagnosis and treatment, advocacy and behavior change, and program management) anchored relevant deductive codes.

**Data corpus and preparation**

Audio-recorded focus group discussions, community consultation meetings, and key informant interviews were transcribed verbatim. Sessions conducted in Nepali or other local languages were translated into English. Thirty rich and representative transcripts (selected to capture diversity by level and stakeholder type) were imported into NVivo 12 for formal thematic analysis; remaining transcripts were reviewed to add contextual details and confirm saturation. Summary matrices and framework charts were developed to help identify recurring patterns across the data.

**Hybrid coding approach**

We applied a hybrid coding strategy that combined (1) *deductive* codes derived from NSP priority domains and Global Fund modules and (2) *inductive* codes that emerged from the corpus (for example, decentralization mechanics, digital equity, and municipal co-financing). Country-dialogue documentation confirmed that strategy review and Global Fund application drafting proceeded in tandem, which reinforced our decision to map all findings first to NSP objectives, then to complementary partner investments.

**Deductive code families and NSP alignment**

We anchored deductive codes to NSP pillars, then cross-walked them to Global Fund modules to preserve funding interoperability where relevant.

- HIV NSP pillars: prevention (including key populations), testing and treatment, differentiated service delivery, community systems and human rights, decentralized stewardship and data use.
- TB NSP pillars: intensified case finding and high-quality treatment (DS-TB and DR-TB), laboratory network quality, public-private mix, subnational stewardship and financing roles.
- Malaria NSP pillars: strategic information and surveillance (including cross-border and check posts), vector control, diagnosis and treatment, social mobilization and BCC, and program management.

**Inductive code families (examples)**

Decentralization operations, municipal planning and budget alignment, digital equity and offline data flows, commodity security and last-mile logistics, adolescent and young-key-population needs, cross-border mobility, private sector engagement, and sustainability and transition planning.

**Coding team and reliability**

Three researchers independently coded a 20 percent subsample for inter-coder reliability, reconciled discrepancies in consensus meetings, refined operational definitions, and then applied the final framework to the full NVivo set. Audit trails, code memos, and framework charts were used to ensure transparency and replicability.

**Deductive modules mapped to NSPs (cross-program)**

Table S4-A. Deductive module to NSP cross-walk

| **Deductive module (GF taxonomy)** | **HIV NSP anchor** | **TB NSP anchor** | **Malaria NSP anchor** | **Illustrative program KPI** |
| --- | --- | --- | --- | --- |
| Prevention: key populations | KP prevention, human rights and community systems; decentralized delivery and data use. | PPM outreach to urban poor, prisoners, factory workers; contact investigation. | Social mobilization, BCC in endemic areas; border check posts. | Coverage among FSW, MSM/TG, PWID; contacts screened; LLIN use in high-risk strata. |
| Treatment and care | Differentiated ART and adherence support via community systems. | DS-TB and DR-TB regimen quality; lab linkages; subnational roles. | Case management, diagnosis and treatment package. | Viral suppression; treatment success; test positivity and time-to-treatment. |
| RSSH (systems) | Community systems strengthening and decentralized stewardship. | HRH, data, governance at provincial and local levels. | HMIS, M&E, and program management strengthening. | Functional EPI/lab networks; timely reporting; stock-out days reduced. |
| Community, Rights, Gender | Anti-stigma, CRG, legal literacy, community-led monitoring. | Stigma reduction to improve adherence and case finding. | Community mobilization and BCC to sustain elimination. | Rights-related barrier indices; client satisfaction; participation metrics. |

**Sample coding framework and codebook excerpt**

Table S4-B. Codebook excerpt (deductive + inductive)

| **Code** | **Definition** | **Include when** | **Exclude when** | **NSP linkage** |
| --- | --- | --- | --- | --- |
| KP prevention | Design and delivery of HIV prevention for FSW, MSM/TG, PWID, migrants, prisoners, AGYW | Stakeholders discuss outreach, safe spaces, combo prevention, legal barriers | General health promotion not specific to KPs | HIV NSP prevention and CRG pillars in decentralized settings. |
| TB case finding | Community and facility case detection, contact tracing, PPM | Mentions of cough screening, private clinics, prisons, DR-TB | Treatment adherence without detection focus | TB NSP case finding and PPM roles across levels. |
| Malaria surveillance | HMIS, EWARS, check posts, case notification | Reporting flows, ABER, district-level indicators | Generic HMIS not tied to malaria | Malaria NSP objectives 1 and M&E indicators. |
| Decentralization | Provincial and municipal roles in planning, budgeting, and oversight | Any reference to federal-provincial-local role clarity or budget alignment | Central-only issues | TB NSP role clarity; HIV NSP decentralized program management. |
| Digital equity | Connectivity, offline tools, phone-based outreach, data timeliness | Solutions for low bandwidth, phone trees, blended virtual-in-person | High-end health IT not used in routine settings | Supports NSP data and monitoring functions across programs. |

**Example of the framework chart used during analysis**

Rows are transcripts or stakeholder subgroups. Columns are cross-disease themes aligned to NSP pillars. Each cell contains a short analytic summary plus one or two illustrative quotations.

Table S4-C. Framework chart (illustrative)

| **Transcript or subgroup** | **Access and equity** | **Stigma and discrimination** | **Community engagement** | **Health system gaps** | **Integration and coordination** | **Sustainability** |
| --- | --- | --- | --- | --- | --- | --- |
| FGD, remote hill municipality | Long travel time and cost for follow-up among seasonal migrants; request for village-based testing days | TB stigma noted from neighbors affecting care-seeking | FCHVs identify chronic cough and encourage testing | RDT and anti-TB drug stockouts reported in last quarter | Referral to district hospital inconsistent; need clearer counter-referral | Local government is willing to budget for outreach if guided by NSP priorities |
| KII, provincial TB officer | Urban poor under-diagnosed; prison intakes missed for TB screening | MDR-TB stigma affects adherence | Peer supporters helpful for follow-up and tracing | Staff turnover and limited lab capacity at hub sites | Interest in co-locating TB and HIV screening in OPDs | Province proposes co-financing for microscopy maintenance with PR support |

**Deductive and inductive coding applied to transcript data**

Table S4-D. Application examples

| **Quote snippet** | **Primary code(s)** | **Secondary code(s)** | **NSP anchor** | **Program implication** |
| --- | --- | --- | --- | --- |
| “We can reach MSM and TG online but follow-up needs a safe drop-in space.” | KP prevention | Digital equity, CRG | HIV NSP prevention and community systems | Blend virtual outreach with drop-in service packages; align GF and PEPFAR scopes to avoid overlap. |
| “Factory workers cough for weeks but do not come to DOTS centers.” | TB case finding | PPM, stigma | TB NSP case finding and PPM | Expand workplace screening, employer referral MOUs, and sputum transport networks. |
| “Border posts see febrile returnees, but data is delayed.” | Malaria surveillance | Cross-border, digital equity | Malaria NSP Objective 1 and check posts | Strengthen notification within 24 hours, pilot web-based reporting and FCHV reporting. |

**From coded themes to NSP, then to complementary financing**

All thematic outputs were first mapped to NSP objectives and only then to complementary financing streams. For example, malaria case management and vector control priorities sit within the Malaria NSP objectives and can be resourced through a mix of domestic co-financing and GF modules that were validated during 19–20 August 2020 meetings, which also summarized GF allocations and government co-financing commitments. Country materials explicitly recognized that NSP review and GF application development occurred simultaneously within the same dialogue platform, preserving national ownership and transparency.

Table S4-E. Traceability crosswalk

| **Coded insight** | **NSP objective link** | **Complementary financing link** | **Implementation action** |
| --- | --- | --- | --- |
| KP prevention gaps for FSW and MSM/TG in urban centers | HIV NSP prevention, CRG, community systems in decentralized settings. | Align PEPFAR HIV focus with GF prevention modules to avoid duplication; document division of labor in task-team minutes | Scale drop-in centers, peer outreach, legal literacy, and client monitoring |
| Missed TB detection in prisons and factories | TB NSP case finding, PPM, lab and referral roles across levels. | Use GF PPM and lab modules to catalyze provincial plans; municipal budget lines for periodic screening | Prison intake screening SOPs; factory MOUs; sputum transport and GeneXpert connectivity |
| Delayed malaria notifications at border points | Malaria NSP Objective 1 (surveillance) and program management. | Combine domestic HMIS upgrades with GF RSSH investments validated at August meeting | 24-hour notification KPI, EWARS refreshers, FCHV reporting pilots, cross-border coordination |

**Analytic matrices and products**

To synthesize findings, we used NVivo matrix queries and exported results into summary matrices and framework charts. These included a province-by-province gap matrix and population-pathway tables that can be expanded into annexes if reviewers request subnational or KP-specific detail.

## Template T1. Province matrix, populated

| **Province or region** | **Access gaps** | **Commodity status** | **Lab status** | **HR status** | **Referral data status** | **Actions agreed** |
| --- | --- | --- | --- | --- | --- | --- |
| Koshi Province border belt | Remote hamlets beyond 2 hours; highly mobile migrants; night service needs near POE | Monsoon RDT gaps; LLIN replacement delays in riverine wards; TB drug buffers thin in 2 municipalities | Microscopy functional; GeneXpert limited to hubs; sample transport irregular | Early turnover of peripheral staff; limited refresher training | POE tallies not linked to eHMIS; cross-border referral logs non-standard | Pre-position RDTs and LLINs pre-monsoon; contract courier for sputum and VL; standardize POE referral form; monthly corridor review; expand GX catchment radius |
| Madhesh border belt | Dense night mobility; KP prefer after-hours access; urban slum pockets underserved | HIV test kits adequate; ART refill continuity gaps during migration; stock alerts slow | GX uptime affected by maintenance; VL shipment to PCR hubs irregular | Peer navigators active but unfunded; shortage of counselors for KP-friendly services | No SOPs for prison and migrant referral; TB-HIV bidirectional register incomplete | Fund peer navigators and weekend clinics; formalize ART refill at border clinics; sign prison and migrant SOPs; vendor maintenance for GX; SMS stock alerts to province store |
| Bagmati urban poor pockets | Travel cost and lost wages; clinic hours conflict with shift work | TB drug stock alerts irregular; HIV commodities adequate; malaria commodities needed for fever triage | Labs close but long queues; GX capacity good with scheduling issues | Staff stretched at high-volume sites; supervision needs | PPM and private notifications weak; pharmacy referrals not captured | Extend hours and Saturday clinics; set buffer stock triggers; fast-track lab triage; enroll pharmacies and private clinics in PPM with simple reporting app |
| Gandaki hills | Long travel and transport cost; seasonal road closures; elderly and disability access gaps | Seasonal LLIN stockouts in remote wards; winter RDT gaps | Microscopy adequate; GX at provincial hospital far for mountain municipalities | FCHV workload high; limited outreach allowances | Referral slips inconsistent across municipalities and district hospital | Quarterly mobile integrated camps; align LLIN delivery with dry-season roads; FCHV micro-grants; standardize referral slips; add tele-consult triage for presumptive TB |
| Lumbini plains | Seasonal migrant peaks at kilns and farms not routinely reached | RDTs usually adequate; occasional HIV kit redistribution needed; TB buffers moderate | Labs fair; QA and PT participation needs strengthening | Volunteers active but untrained in ACF micro-planning | Inter-district referral not standardized; paper trail lost when migrants move | Run migrant screening bursts at worksites; quarterly lab QA/PT; train volunteers in ACF micro-plans; inter-district referral SOP with WhatsApp handover |
| Karnali mountains | Very remote access; weather isolation; catastrophic transport cost for patients | Winter stockouts of TB drugs and RDTs due to road closure; no LLIN buffer | Lab coverage patchy; GX absent in several districts; courier ad hoc | Few skilled staff; rotation gaps; limited biomedical support | Data delays from remote wards; monthly aggregation only | Combined outreach treks for TB-HIV-malaria; pre-position winter stocks via airlift; fixed courier days; portable digital X-ray pilot; SMS rapid reporting from remote posts |
| Sudurpashchim far west | Cross-border seasonality; forest goers and fishers miss routine services | LLIN replacement uneven; malaria RDT gaps in peak months; TB commodities stable | Labs OK in hubs; microscopy coverage fair; EQA feedback slow | Trained staff present but supportive supervision infrequent | Cross-border data sharing ad hoc; no shared dashboard with adjacent districts | Synchronize LLIN replacement with migration calendar; surge supervision; sign cross-border data-sharing note; simple line-list exchange each month |
| National cross-border corridor cluster | Multiple POE points with inconsistent screening and referral; language barriers | POE kits available but replenishment slow; IEC not localized | On-site testing limited; district labs handle confirmation | POE staffing rotates without formal handover | POE data not mapped to facility line lists; duplicates in counts | Establish corridor micro-plans; bilingual IEC; POE to facility QR referral handover; weekly reconciliation between POE and facility lists |
| Refugee and special settlements | Movement restrictions; documentation barriers; limited KP-friendly access | HIV test kit shortages periodic; malaria commodities adequate; TB drug continuity at transfer risk | Camp clinics functional; confirmatory capacity limited | Peer educators present; refresher training intermittent | Referral MOUs partial; feedback to camp clinics slow | Renew two-way MOUs; designate district focal person; scheduled medication handover on transfers; finance peer refresher trainings |

## Template T2. Population pathway, populated

| **Population** | **Entry point** | **Barriers** | **Engagement model** | **Integration needs** | **Financing alignment** | **KPIs** |
| --- | --- | --- | --- | --- | --- | --- |
| People living with HIV | OPD, KP clinics, peer networks | Stigma and privacy concerns; rigid clinic hours; transport cost | Peer navigation; community-led testing; multi-month dispensing and flexible pickup | Viral load testing via GeneXpert and PCR hubs; GBV screening and referral; TB screening each visit | GF funds CLT and lab networking; PEPFAR supports KP and peers; GoN co-finances commodities | VL coverage and suppression; 12-month retention; TB screening at each visit |
| Presumptive TB and TB survivors | DOTS, pharmacies, FCHVs | Lost wages and travel cost; MDR stigma; delayed diagnosis | FCHV cough screening; sputum collection and transport; survivor mentors | Bidirectional TB-HIV testing; oxygen at district level; social protection linkage | GF supports ACF, courier, GX; GoN funds oxygen and DOTS; partners support survivor groups | Time to diagnosis; TB patients with HIV test; catastrophic cost index; treatment success |
| Female sex workers | Night outreach, drop-in centers, KP clinics | Discrimination and safety risks; policing concerns | Peer-led night outreach; safe-space clinics; discreet testing and linkage | SRH and HIV integration; legal aid referral; violence mitigation protocols | PEPFAR leads KP packages; GF complements outreach and CLM; local MOUs with municipalities | FSW reached; testing uptake; linkage to prevention or ART; violence reports addressed |
| MSM and TG | KP clinics, peers, social venues | Stigma; documentation issues; youth barriers | Peer linkage; youth-friendly hours; online booking and outreach | Mental health within HIV services; hormone therapy linkage for TG where available | PEPFAR supports KP and youth; GF funds stigma reduction and CLM; GoN prepares facility readiness | MSM TG testing coverage; time to ART start; satisfaction with KP-friendly services |
| People who inject drugs | OST sites, harm reduction outreach | Criminalization fears; instability; overdose risk | OST plus harm reduction outreach; peer-led CLM | Integrated HIV and HCV testing and care; overdose prevention; TB screening in OST | PEPFAR and GF coordinate OST and harm reduction; GoN transitions OST costs | OST coverage; HIV HCV testing uptake; needle-syringe distribution; OST retention |
| Migrants and spouses | POE desks, return villages, transport hubs | Missed testing windows; cost; frequent moves | Community-led testing at border and return villages; travel vouchers | Cross-border referral with feedback loop; malaria fever desks; TB continuity | GF funds CLT and POE systems; GoN funds vouchers; partners support cross-border linkage | Percent screened; successful cross-border referrals; treatment initiation within 7 days |
| Prison inmates | Intake health checks, prison clinic | Transfer interruptions; stigma; poor privacy | Peer educators in prisons; routine intake and periodic mass screening | TB-HIV integrated screening; ART and DOT continuity; MDR isolation protocols | GF supports prison package; GoN and MoHA fund SOPs and continuity; partners train peers | New entrants screened within 48 hours; continuity on transfer; ART interruptions per 100 inmates |
| Adolescent girls and young women | Schools, youth clinics, community groups | Limited youth-friendly hours; fear of disclosure | Youth peer educators; school-based SRH and HIV awareness; hotline and chat | Link HIV prevention with SRH, GBV support, mental health | PEPFAR supports youth where applicable; GF supports IEC and CLM; GoN funds youth corners | Number reached with comprehensive package; testing uptake; GBV referrals completed |
| Elderly and geriatric populations | OPD, home visits via FCHVs | Mobility limits; comorbidities; low TB suspicion | Home-based screening and sputum pickup; caregiver engagement | TB screening integrated with NCD visits; palliative support linkage | GF supports community screening; GoN funds home-visit allowances; partners provide assistive aids | Elderly screened for TB annually; treatment initiation time; follow-up completion |
| Marginalized caste or ethnic groups including Mushar | Community meetings, FCHVs | Extreme poverty; social exclusion; low health literacy | Peer mobilizers from same community; targeted outreach and incentives | Combined TB-HIV-malaria screening; nutrition and social protection link | GF supports outreach; GoN funds social protection linkages; CSOs implement | Outreach coverage in target settlements; testing uptake; initiation and completion rates |
| Refugees and camp residents | Camp clinics, UN partner referrals | Documentation and movement restrictions; language barriers | Peer educators; bilingual IEC; escorted referral to public facilities | HIV, TB, malaria referral MOUs; feedback loop to camp clinic | Partners support camp services; GF complements testing; GoN provides confirmatory care | Number referred and received; turnaround for confirmation; treatment start rate |
| Forest goers and agricultural workers | Worksite outreach, FCHVs, VMWs | Distance; seasonality; low net use during peak work | Fever desks at farms; net-use reminders; mobile testing | Private clinics and pharmacies linked for RDT and TB triage | GF funds VMWs and RDTs; GoN funds LLINs and microscopy; private sector collaborates | Fever cases tested within 24 hours; LLIN use rate; positivity in hot spots |
| Private sector first-contact clients | Pharmacies, private GP clinics | Cost; confidentiality; lack of referral pathways | PPM enrollment; discreet referral cards; simple app reporting | TB-HIV co-testing pathways; malaria RDT availability in accredited outlets | GF funds PPM systems; GoN accredits outlets; associations co-implement | Private notifications captured; referrals completed; time from first contact to diagnosis |

**Quality assurance, bias mitigation, and ethics**

- Triangulation: Consultation narratives were triangulated with program data and NSP targets and roles. HIV NSP consultation emphasis and systems focus provided the normative reference.
- Subnational balance: Codes incorporated federal, provincial, and local roles consistent with TB NSP governance delineation; malaria surveillance codes reflected check-post and border operations described in the Malaria NSP.
- Consultative validity: Prioritization and validation meetings in August 2020 were used to confirm interpretations before final mapping to NSP results frameworks, including GF module alignments and government co-financing lines.

**Limitations**

The qualitative corpus reflects a period that included COVID-19 restrictions, which may have shaped emphasis on digital access and continuity solutions. Although saturation was exceeded through iterative rounds, some very remote or hidden populations may still be under-represented in verbatim quotes. These limitations were mitigated by repeated stakeholder validation and explicit alignment to NSP objectives and indicators.

**Summary**

This hybrid coding approach ensured that findings from national consultations were first and foremost mapped to the HIV, TB, and Malaria NSPs, with Global Fund and USAID/PEPFAR positioned as complementary financing and delivery mechanisms. Deductive codes grounded the analysis in NSP pillars and GF modules; inductive codes captured operational realities of decentralization, equity, and sustainability. Matrix outputs and framework charts provided traceable lines from raw data to NSP objectives, indicators, and practical implementation actions, strengthening the credibility and national ownership of the final strategies.
